# Instant Detection of Synthetic Cannabinoids on Physical Matrices, Implemented on a Low-Cost, Ultraportable Device

**DOI:** 10.1021/acs.analchem.3c01844

**Published:** 2023-08-29

**Authors:** Gyles E. Cozier, Rachael C. Andrews, Anca Frinculescu, Ranjeet Kumar, Benedict May, Tom Tooth, Peter Collins, Andrew Costello, Tom S. F. Haines, Tom P. Freeman, Ian S. Blagbrough, Jennifer Scott, Trevor Shine, Oliver B. Sutcliffe, Stephen M. Husbands, Jonathan Leach, Richard W. Bowman, Christopher R. Pudney

**Affiliations:** ^†^Department of Life Sciences, ^‡^Department of Chemistry, and ^§^Centre for Sustainable and Circular Technologies, University of Bath, Bath BA2 7AY, U.K.; ∥ Department of Analytical, Environmental and Forensic Sciences, King’s College London, 150 Stamford Street, London SE1 9NH, U.K.; ⊥ TICTAC Communications Ltd., Room 1.159 Jenner Wing, St. George’s University of London, Cranmer Terrace, London SW17 0RE, U.K.; # HMP Bristol, 19 Cambridge Road, Horfield, Bristol BS7 8PS, U.K.; ∇ Avon and Somerset Police, Valley Road, Bristol BS20 8JJ, U.K.; ○ MANchester DRug Analysis & Knowledge Exchange (MANDRAKE), Department of Natural Sciences, Manchester Metropolitan University, Manchester M1 5GD, U.K.; ◆ Greater Manchester Police, Openshaw Complex, Lawton Street, Openshaw, Manchester M11 2NS, U.K.; ^¶^Department of Computer Science and ^⋈^Department of Psychology, University of Bath, Bath BA2 7AY, U.K.; ⧓ Centre for Academic Primary Care, Bristol Medical School, Bristol BS8 2PN, U.K.; ⧖ Institute of Chemical Sciences, Heriot-Watt University, Edinburgh EH14 4AS, U.K.; ● School of Physics and Astronomy, University of Glasgow, Glasgow G12 8QQ, U.K.; ¤ Centre for Therapeutic Innovation, University of Bath, Bath BA2 7AY, U.K.

## Abstract

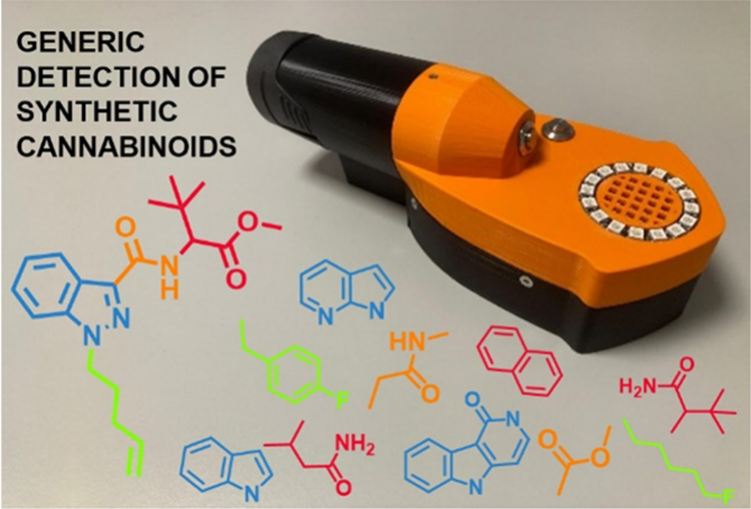

Synthetic cannabinoids (SCs) make up a class of novel psychoactive substances (NPS), used predominantly in prisons and homeless communities in the U.K. SCs can have severe side effects, including psychosis, stroke, and seizures, with numerous reported deaths associated with their use. The chemical diversity of SCs presents the major challenge to their detection since approaches relying on specific molecular recognition become outdated almost immediately. Ideally one would have a generic approach to detecting SCs in portable settings. The problem of SC detection is more challenging still because the majority of SCs enter the prison estate adsorbed onto physical matrices such as paper, fabric, or herb materials. That is, regardless of the detection modality used, the necessary extraction step reduces the effectiveness and ability to rapidly screen materials on-site. Herein, we demonstrate a truly instant generic test for SCs, tested against real-world drug seizures. The test is based on two advances. First, we identify a spectrally silent region in the emission spectrum of most physical matrices. Second, the finding that background signals (including from autofluorescence) can be accurately predicted is based on tracking the fraction of absorbed light from the irradiation source. Finally, we demonstrate that the intrinsic fluorescence of a large range of physical substrates can be leveraged to track the presence of other drugs of interest, including the most recent iterations of benzodiazepines and opioids. We demonstrate the implementation of our presumptive test in a portable, pocket-sized device that will find immediate utility in prisons and law enforcement agencies around the world.

Synthetic cannabinoids (SCs; Spice; K2) are a structurally diverse class of novel psychoactive substances (NPS). The major body of evidence suggests that these are cannabinoid receptor agonists, hence the common acronym synthetic cannabinoid receptor agonists (SCRAs), but there is mounting evidence for interaction with other receptors and enzymes.^1−3^ We therefore prefer the term SC. Over 235 SCs are monitored by the European Monitoring Centre for Drugs and Drug Addiction (EMCDDA),^4^ and evidence has shown that the evolution of their structure over the past 10 years tracks with trends in global legislation to prevent their use.^5^ SCs are defined as having “tail”, “core”, “linker”, and “linked group” moieties, each of which is synthetically interchangeable, while often retaining the agonism of cannabinoid receptors (CB1 and CB2).

SCs are the dominant NPS and one of the most used drugs within the prison estate.^6^ The majority of prison residents in England have used SCs and to a higher rate than other NPS.^6,7^ As full CB1 agonists, the side effects of SCs are debilitating and can include psychosis, stroke, and seizures, and they are associated with aggression.^8,9^ Indeed, 67% of prison staff claim prisoner use of NPS has had a deep impact on their work, with 91% having witnessed aggression at least once and 53% experiencing direct harm.^6^ Enhanced detection was identified as the key mechanism to alleviate the operational challenges of NPS use in U.K. prisons.^6^

SC-soaked personal mail is a well-established mechanism of entry of SCs into prisons and can be effectively ameliorated through screening of personal mail and photocopying.^10^ However, in the U.K., the “Rule 39” mail sent from legal professionals to prison residents contains confidential personal information and is passed directly to residents, posing a significant detection challenge.^11^ Moreover, alternative drug entry routes have increased, including soaking SCs into fabric, “street” herb materials, “throw-overs”, and staff corruption, and as yet unidentified matrices are expected to grow in prevalence.^10^

Typically, lab-based identification is achieved through hyphenated techniques such as gas chromatography mass spectrometry (GC-MS) or liquid chromatography mass spectrometry (LC-MS).^12^ In the field, portable, rapid, and of low technical complexity solutions are favored. For example, devices that utilize ion mobility spectrometry (IMS), Raman spectroscopy, and near-infrared (IR) (NIR) spectroscopy are commercially available and have been successfully implemented in many prisons and drug checking sites globally.^11,13^ However, these approaches are often challenged by samples containing mixtures of SCs, particularly when present on complex matrices as described above, which can lead to false negatives in detection.^14,15^ Moreover, concentration range sensitivity on these devices (both low and high) can present a challenge.^16,17^ Where the approach relies on a library of spectral data, accurate detection is necessarily tied to the pace of change of the drug of interest as well as the rate of identification, qualification, and software update.^11,18^

We have previously demonstrated that SCs can be accurately detected using fluorescence spectral fingerprints (FSFs)—enumerated excitation emission matrices.^19,20^ The FSFs are discriminatory of SCs both generically and as structural classes. An example is shown in Figure 1A. The FSF approach has the best utility where the SC is present in a complex matrix, e.g., saliva, where spectral deconvolution from a complex background is needed (e.g., salivary protein mission arising from intrinsic tryptophan residues). Such detection would be beneficial for point-of-care analysis to assess patients who are nonresponsive but are suspected of SC use.

**Figure 1:**
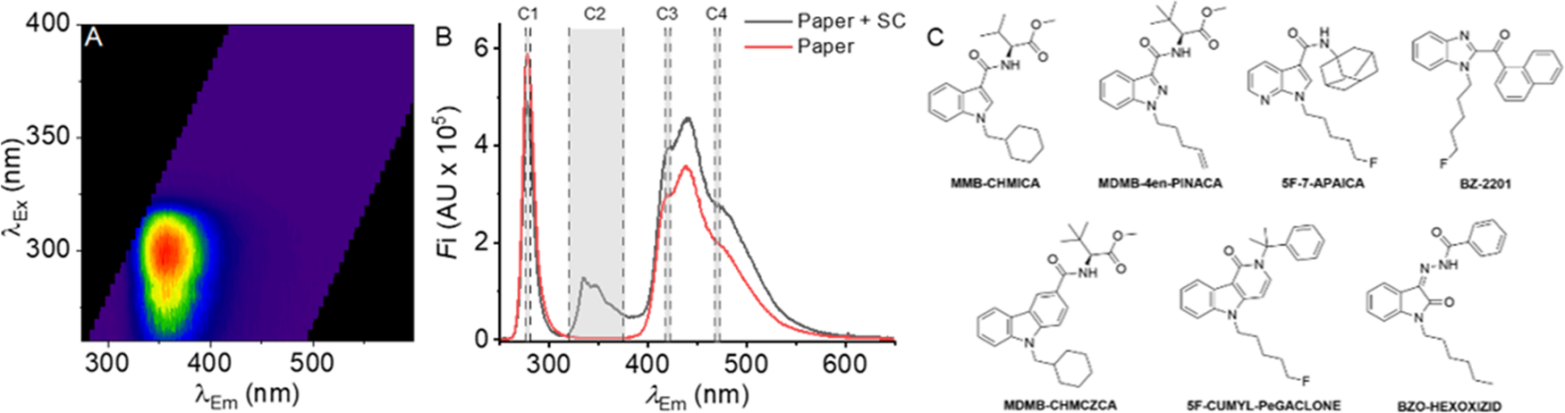
(A), Fluorescence spectral fingerprint (FSF) of SC extracted from a seized herbal sample (5F-MDMB-PINACA). (B) Example spectra showing spectral signatures on white paper in the presence (black; MDMB-4en-PINACA) and absence (red) of SCs. C1 – C4 correspond to the spectral windows captured by each of the band-pass filters in the device described below. Excitation for the spectra was at 265 nm. (C) Range of “core” moieties that give rise to SC fluorescence.

From Figure 1A, the typical SC FSF has a major emission band centered at ∼330–365 nm. This is the case for the vast majority of SCs surveyed across diverse structural classes (Figure S1), including the most recent iterations of seized SCs, including “tail-less” molecules such as MDMB-5Br-INACA, recently detected in Scottish prisons.^21^ The emission wavelength is broadly defined by the nature of the core moiety, e.g., typically indole- or indazole-based. However, more recent variants show a similar emission profile, i.e., broadly centered at ∼330–365 nm. The notable exception are OXIZIDs, a recent generation of SCs with an oxoindole core,^21,22^ where the emission band is red-shifted, and we discuss this below. Examples of SCs showing the range of “core” moieties are shown in Figure 1B. That is, the vast majority of SCs we have observed across all major structural classes exhibit a similar structured emission band with a strong signal at ∼350 nm. Potentially, generic discrimination of SCs might not require a full FSF but instead observation of the major emission band using a single excitation wavelength. We hypothesize that with a sufficiently intense irradiation source, SC fluorescence might be observable deconvolved from the autofluorescence of the physical substrate onto which it is adsorbed, without requiring a full FSF to be collected. Herein, we detail the confirmation of this hypothesis and its implementation into an ultraportable, hand-held device, allowing instant detection of SCs on a broad range of materials. We further illustrate the potential of this approach to detect other drugs of abuse.

## Results and Discussion

### SC Detection in a Spectrally Silent Region of Typical Physical Matrices

Solid matrices, including paper, fabric, and herb material, are spectrally silent in the region ∼350 nm, when irradiated with a UV–C light source. Figure 1B shows an example of spectra acquired via excitation using a 265 nm light-emitting diode (LED) for a seized paper sample in the presence and absence of an SC. These data are collected via direct irradiation of the sample (no extraction of the sample required), with spectral acquisition via a fiber optic attached to a spectrometer. Typically, the irradiation occurs ∼2 cm from the sample, with light collection at the tip of the fiber ∼2 cm from the sample. Integration times were ∼20 s. From Figure 1B, the spectra can be categorized into several spectral regions (C1–4), and the combined data for the integrated fluorescence intensities in each region for a range of materials (>200 samples) are shown and discussed in subsequent analyses below.

The C1 region (265 nm, 5 nm bandwidth) corresponds to reflected light from the C1 LED. The reflected light retains the spectral characteristics of the incident source, including the center wavelength and bandwidth. The magnitude of detected light in this spectral region will vary according to the intensity of the source, the absorption of the incident light by the sample matrix (including the analyte), and specular reflection.

The C2 region (∼325–375 nm) corresponds to a spectrally silent region (and is illustrated for multiple solid matrices below). This spectral region is an absolute minimum of the acquired spectrum when it is irradiated at UV–C wavelengths. To note, we do not anticipate that this region is spectrally silent at all excitation wavelengths. The total emission in this region will be determined by the background signal (primarily arising from the irradiation source; C1), the quantum yield of the SC, its concentration, and quenching by the matrix material including via fluorescence resonance transfer (FRET) to endogenous molecules (see below). At concentrations ≲1 mg cm^–2^, on paper, we find that the intensity attributable to the SC concentration is effectively linear. At higher concentrations, we find that the signal saturates as one anticipates from the classical inner filter effect.

The C3–C4 region (∼375–600 nm) corresponds to a spectral feature that we anticipate arises from optical brightening agents (OBAs, e.g., stilbenes) within the material that tend to emit in this specific spectral region.^23^ However, lignin and cellulose emission has also been reported in a similar spectral region.^24,25^ Potentially, the OBAs enhance natural fluorescence arising from the lignin/cellulosic material. At least for the range of samples we have studied, we find that a large emission band in the C3–C4 region is correlated with optically bright materials, e.g., being hardly present in brown paper/black fabric (Figure S4B). That is, any material treated with such agents is likely to display a similar emission profile. Materials that are not treated with such agents may display diffuse emission bands that can be attributed to endogenous fluorophores or scattering. In practice, we find that matrices such as brown papers and untreated fabrics have exceptionally low emission in this spectral region so as to be effectively spectrally silent. Surprisingly, we find that where there is a significant signal in this spectral region, the ratio of C3/C4 shoulders of this spectral feature is almost invariant regardless of the material type (Figure 1C). For the range of materials we have studied, the C3/C4 ratio has an average and standard deviation of 1.4 and 0.3, respectively.

Given that the C2 region is spectrally silent for essentially all paper, fabric, and herb materials we have surveyed, we hypothesized that detection of SCs would be possible by sensitively monitoring emission in this region, subtracted from the background. We tested our hypothesis using seized, suspected SC materials, obtained from the Avon and Somerset Police between 2017 and 2021, adsorbed onto different physical matrices. To that end, 30 seized samples (ASP 1–30) of plant materials were extracted into methanol and analyzed using a combination of LC-MS, nuclear magnetic resonance (NMR), and thin-layer chromatography (TLC). The identity of SC compounds in these samples are reported in Table S1. Out of these samples, 6 samples contained no SC. TLC was used for the initial comparison and as an indication of the number of SC compounds present. Running NMR analysis in tandem with LC-MS detection was invaluable for identifying compounds with virtually indistinguishable mass spectra, e.g., 5F-PB-22 and 5F-MDMB-PICA.^26^ Using these data as a library, further 4 plant materials and 7 paper seized samples were analyzed with NMR (Table S1), which identified 3 plant and 2 paper samples that contained SC compounds.

**Figure 2:**
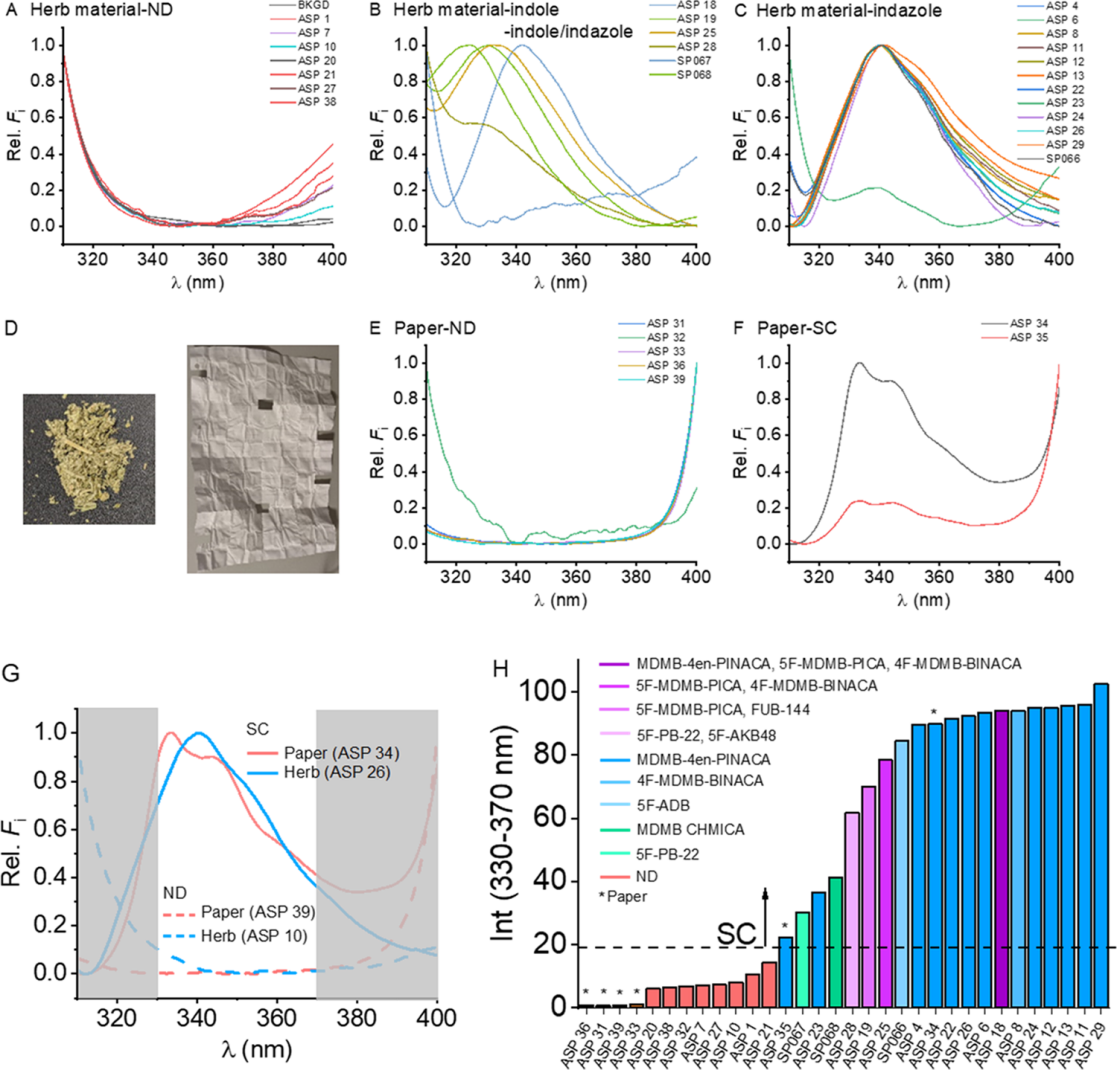
Direct spectral acquisition of samples seized by Avon and Somerset Police, suspected to have SCs present. (A–C) Herb samples and (E, F) paper samples, as exemplified in panel (D). (G) Highlighted spectrally silent C2 region (as in Figure 1) with examples from the panels above paper and herb spectra with and without SC. (H) Integral of the spectral C2 region, ranked by magnitude and annotated with the detected material in the inset. Excitation for spectra was at 265 nm. Rel. *F*_i_ = relative fluorescence intensity; ND represents no detection (no drugs of abuse).

Figure 2 shows the spectra acquired from these samples. The excitation source is centered at ∼265 nm to prevent the spectral overlap of the excitation source into the spectral C2 region, and we discuss this in more detail below. These samples include herblike samples consisting of inert plant materials soaked/sprayed with SCs (Figure 2A–C) and SCs soaked/sprayed onto paper (Figure 2E,F), primarily destined for illicit entry into prisons via the normal postal system. Figure 2D shows exemplars of these materials, although they vary significantly in presentation. Figure 2G shows example processed spectra in which SCs were detected on the physical matrix with no processing. Figure 2H shows the integral of the C2 region for each of the samples tested. The coloration reflects the detection for each sample, named in the inset where ND = nothing detected (no SC).

From Figure 2H, in the presence of SCs, integrating the signal of the spectral C2 region yields a signal that is distinguishable from a “negative” background in all cases. Our data are consistent with European SC seizure data from 2020, where MDMB-4en PINACA is the dominant SC detected (∼50% of cases).^4^ We note that in two of the “negative” samples, we detect cannabis via both GC-MS and NMR. While there are several reports of cannabis giving a measurable fluorescence signal, at least for the excitation wavelengths used here, there is no detectable signal.^27,28^ Moreover, while our NMR data suggest the major compounds present in the seized material are SCs, there are also detectable amounts of other drugs of abuse via LC-MS including MDMA, heroin, cocaine, and cannabis, though we anticipate that some of these may be present due to cross-contamination. As we expect from the structures, our data do not show a signal arising from these molecules (at least at the concentrations present) nor do we expect any significant quenching effect from such molecules.

### Instant, Ultraportable Device with Dynamic Background Scaling

**Figure 3:**
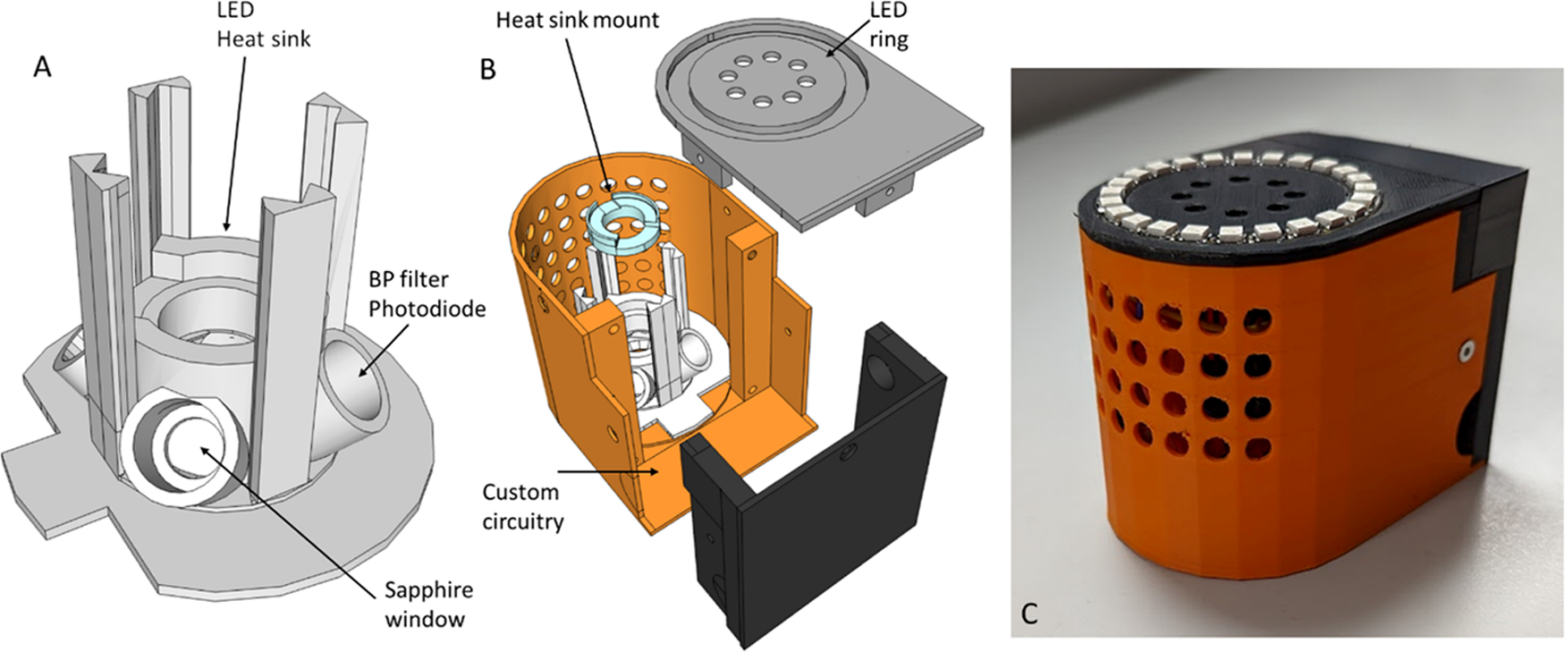
Schematic of the SC detection device. Panels (A, B) show a three-dimensional (3D) printed housing for an assembly comprising the following: Band-pass (BP) filters corresponding to each spectral region (C1–4; Figure 1B) are paired with a powered photodiode (PD) optimized for that spectral region. A high-powered (HP) LED with a heat sink is mounted centrally (45 °C) relative to the BP-PD pairs. The optical elements are protected via a sapphire window aligned with the optical path and focal length of the PDs. The assembly is protected by an exterior shell with integrated vents aligned with the heat sink of the LED. User signal reporting is via an integrated LED ring on the top of the device. PD and LED controls are via a microcontroller and an analog to digital converter (ADC), internal to the device. Panel (C) shows the external case, and further views of the fully constructed device are given in Figure S2.

The data in Figure 2 suggest that the integration of spectral data could be a means to identify SCs on complex matrices without any processing of the sample. We have therefore developed a device capable of high sensitivity detection within each of the spectral regions of interest (Figure 2). The device is shown in Figure 3. The device consists of an array of photodiodes (PDs) with wavelength selection via a band-pass (BP) filter. Irradiation is achieved via a high-power LED coupled to a heat sink, with a nominal output power of ∼50 mW and a full width at half-maximum (fwhm) of ∼12 nm. The BPs used are centered at 265, 350, 420, and 470, with fwhm’s of 5, 50, 5, and 5 nm, respectively, as shown in Figure 1B, and they are correspondingly designated C1–C4. The PDs are amplified and with detection maxima optimized for the spectral region of interest, as described in the Materials and Methods section. Data for each of C1–C4 are collected via an analog to digital converter (ADC) and passed to a microcontroller. The data are returned as a raw signal, essentially a voltage. The data are then numerically manipulated (described below) to give a visual report of the presence and absence of SCs through lights on the exterior of the device (Figure 3C). Also see Figure S2 for further views of the device, including a battery-operated version. The data recorded from each of the four channels (C1–C4) do not show significant variation with respect to time (decrease <5% over 2 h for all channels) as shown in Figure S3. We posit that the small amount of variance is related to internal heating from the heat sink. We note that in practice, the LED is not “on” all of the time that the device is powered, with an automatic cutoff when the device is placed in its side. However, these data demonstrate that the data are consistent and reproducible with the device.

Typically, SCs have excitation maxima at 280–320 nm (Figures 1A and S1). However, we find that when SCs are adsorbed onto paper, the excitation spectrum is significantly flattened compared to that in methanol (Figure S4; MDMB-4en-PINACA). For the example in Figure S4F, excitation at 265 nm gives ∼50% of the relative emission as excitation at the excitation maximum. This compares to ∼30% for the same SC in methanol. That is, in a practical sense, there is only a small advantage in exciting samples at ∼300–310 nm, which would otherwise cause a large overlapping signal in the C2 region (Figure 1B), where we anticipate the SC emission to arise. Instead, these data demonstrate that one can utilize a very high-power (∼50 mW) excitation source that is spectrally separate from the emission band of interest, thus achieving the largest emission signal balanced against the smallest background. For this reason, we opted to use an excitation source at 265 nm, which is the lowest wavelength/highest power LED available at the time of writing.

From Figure 2H, the magnitude of the SC signal may be varied. Potentially, one can apply an empirical threshold based on the observation of a large number of samples with known backgrounds. However, such an approach is very likely to lead to a significant number of false negatives and false positives arising from low concentration/quantum yield SCs or high background matrices, respectively. Ideally, one would *a priori* know the background signal and the expected magnitude of signal change for an SC on any given physical matrix.

**Figure 4:**
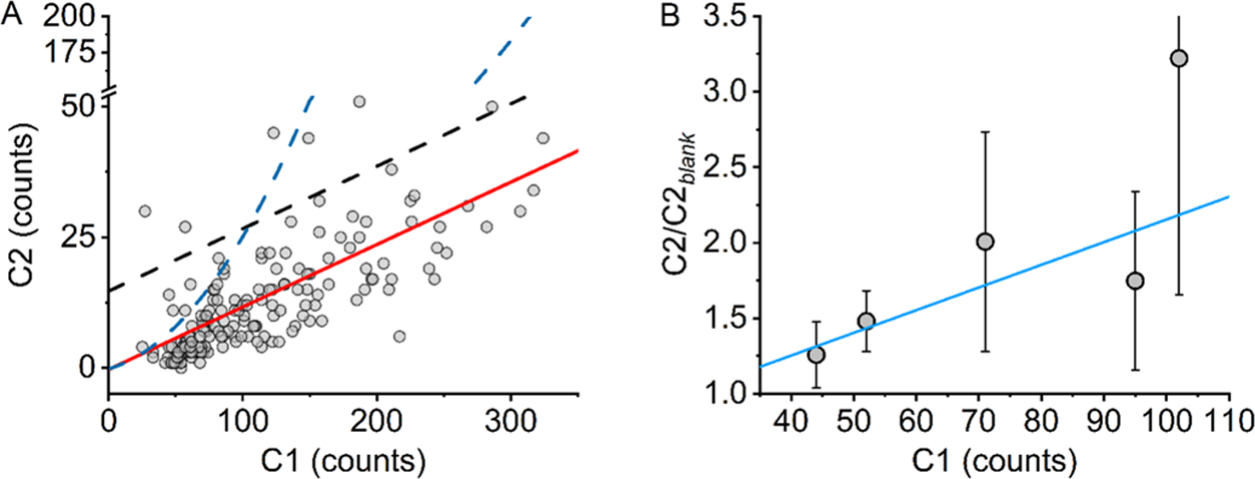
Predictive model for quantifying the background signal arising from the abroad range of physical matrices collected on the device described in Figure 3. (A) Relationship between the magnitude of C1 and C2. The solid fitted red line is a simple exponential function fitted to the data. The black dashed line shows the red trend line scaled to give a threshold with a ∼5% false positive rate. The blue dashed is the threshold based on how the C2 signal of SCs varies on matrices as shown in panel B. (B) How the C2 signal varies for a range of SCs on a selection of white and brown papers. Plot of the average ratio of the measured C2 magnitude of paper impregnated with SCs (as in the main text at ∼10 μg cm^–2^) and the value of that paper alone (C2_blank_) versus the measured C1 value of the paper.

Figure 4A shows the plot of the values of C1 versus C2 for a range of physical matrices including paper (a range of colored paper with a range of inks, including printed, crayon, pencil, etc., and paint), fabric (cotton, a range of colors and textures), and herb materials. From Figure 4A, as the magnitude of C1 increases, C2 increases. These data can be fit using a linear relationship. This relationship is shown as the fitted solid red line in Figure 4A. That is, there is a strong positive correlation relationship (Pearson’s *r* = 0.72) between the magnitudes of C1 (the reflected light from the irradiating LED) and C2 (the background arising from the LED and any autofluorescence of the material). The presence of this relationship seems logical since as a material becomes more absorptive, the background signal arising from the irradiation will decrease and *vice versa*. We anticipate that the trend in Figure 4A is composed of a sum of correlative relationships for individual materials/conditions. However, the simplified trend shown in Figure 4A gives a useful framework, as we describe below.

The consistent trend followed in Figure 4A provides a means to calculate the predicted background at C2 (C2_pred_) from a collected reading of C1. That is, the red fitted line in Figure 4A reflects C2_pred_. The fitted model shown in Figure 4A (red line) gives an average ratio of C2/C2_pred_ = 1.0 and a standard deviation of 0.8. These data demonstrate an accurate, predictive model for the background signal arising in the spectral region where SC fluorescence emission is detected. From Figure 4A, using this C2_pred_ solely to assess a cutoff for the presence of SCs will lead to a large fraction of false positives. A simple route to tackling this is to arbitrarily scale C2_pred_ to give an acceptable false positive rate. The black dashed line in Figure 4A shows an example of such a scaling, giving a threshold with a ∼5% false positive rate.

Next, we assessed how the signal of C2 varied for a range of SCs, at similar concentrations, on a range of different matrices with increasing C1 signals. The matrices include a range of white and brown papers (5 in total) for each of 5 SCs: MDMB-4en-PINACA, ADB-P7AICA, MMB-CHMICA, MDMB-4en-PICA, and 5F-AKB-48. Given that we know the quantum yield of SCs will vary, we measure the ratio of the measured magnitude of C2/C2_blank_, where C2_blank_ is the C2 signal of the matrix with no SC added. That is, a value above 1 is attributable to the SC alone. Figure 4B shows the relationship between the average magnitude of this ratio and the measured value of C1. From Figure 4B, there is an evident increase in the ratio of the SC signal for the same molecules as the magnitude of C1 increases on different matrices.

The trend in Figure 4B can be quantified using a simple linear function (blue fitted line in Figure 4B). That is, the greater the light absorption of the material (smaller C1), the smaller the increase in signal at C2 for the same concentration of the analyte. This indicates that detection could be more sensitive on more highly reflective materials, such as white paper, that are commonly used for drug entry routes into prisons. We can convolve this numerical relationship with the predicted background in Figure 4A to give a threshold for detection of the analyte, shown as the dashed blue line in Figure 4A. Signals above this threshold trigger the device alarm (lights). This function then accounts for both the change in the predicted background and the anticipated increase in the magnitude of C2 at various C1 values. This approach gives a similar false positive rate compared to the arbitrary scaling (dashed black line in Figure 4A) of ∼6%.

Our data then provide two key pieces of information. First, there is a quantifiable relationship that relates the magnitudes of C1 and C2; we can accurately predict the background signal of C2 using a simple function. Second, scaling the C2_pred_ values to produce a threshold for SC detection can be tailored to balance sensitivity and specificity. Combining these thus provides a numerical model for the detection of SCs on a huge range of diverse background materials and is scalable with SC concentration. Signals at C2 above the threshold line are then termed as “positive” for the presence of SCs. We note that this curve can be scaled to remove false positives with a concomitant decrease in concentration sensitivity. From these data used to develop the model, scaling the threshold to give rise to a false positive rate of ∼5% (Figure 4A) gives a detection limit of ∼10 μg cm^–2^. We note that a recent study placed the lower limit of “real” SC concentrations on physical matrices at ∼50 μg cm^–2^ and so our model appears to balance detection sensitivity and false positive rate adequately for real samples.^29^

Figure 3 shows the design of a device that integrates both the optical detection methodology and the numerical analysis, as described above. Using this device, we have assessed detection on a very large range of matrices including paper (white, brown, blue, pink, printed, and unprinted), fabric (cotton and synthetic at a range of colors and thicknesses), and herb materials (as described above). We show a video of the device working with a range of seized materials in Movie S1.

In an effort to assess the sensitivity and specificity of this approach and device with real-world data, we conducted a trial of a large number of paper (letters, cards, etc.) samples seized from prisons that were analyzed by TICTAC Communications Ltd. using the device described above. The presence of SCs and other molecules of interest was established via GC-MS. Clearly, the detection sensitivity of the device will be vastly less than GC-MS, and so the device will not be competitive in this sense. Therefore, to give a realistic assessment of the device, we removed samples that only showed trace amounts of SCs detected by GC-MS and envelopes that had contained positive SC materials. This resulted in 181 samples tested with the device (Table S2). From these data, we found that a ∼6% false positive threshold set for the device gave a sensitivity of 73% and a specificity of 94%, whereas, with a ∼12% false positive threshold, the device had 78% sensitivity and 88% specificity. This highlights how the thresholding to detection can be increased or decreased to balance false positives against sensitivity, as desired. The degree of difference this makes to sensitivity will depend on the number of borderline samples, those containing lower concentrations or lower quantum yield SCs, that are present. Given we are measuring sensitivity against GC-MS, which has at least a 3 orders of magnitude better detection threshold, ∼ng versus ∼μg—the sensitivity value is likely to be a significant underestimate. That the sensitivity value is so high therefore reflects a very competitive detection modality. These data therefore point to an extremely effective tool in the rapid screening of materials for the presumptive presence of SCs.

### Potential for the Detection of Nonfluorescent Molecules on Physical Matrices

**Figure 5:**
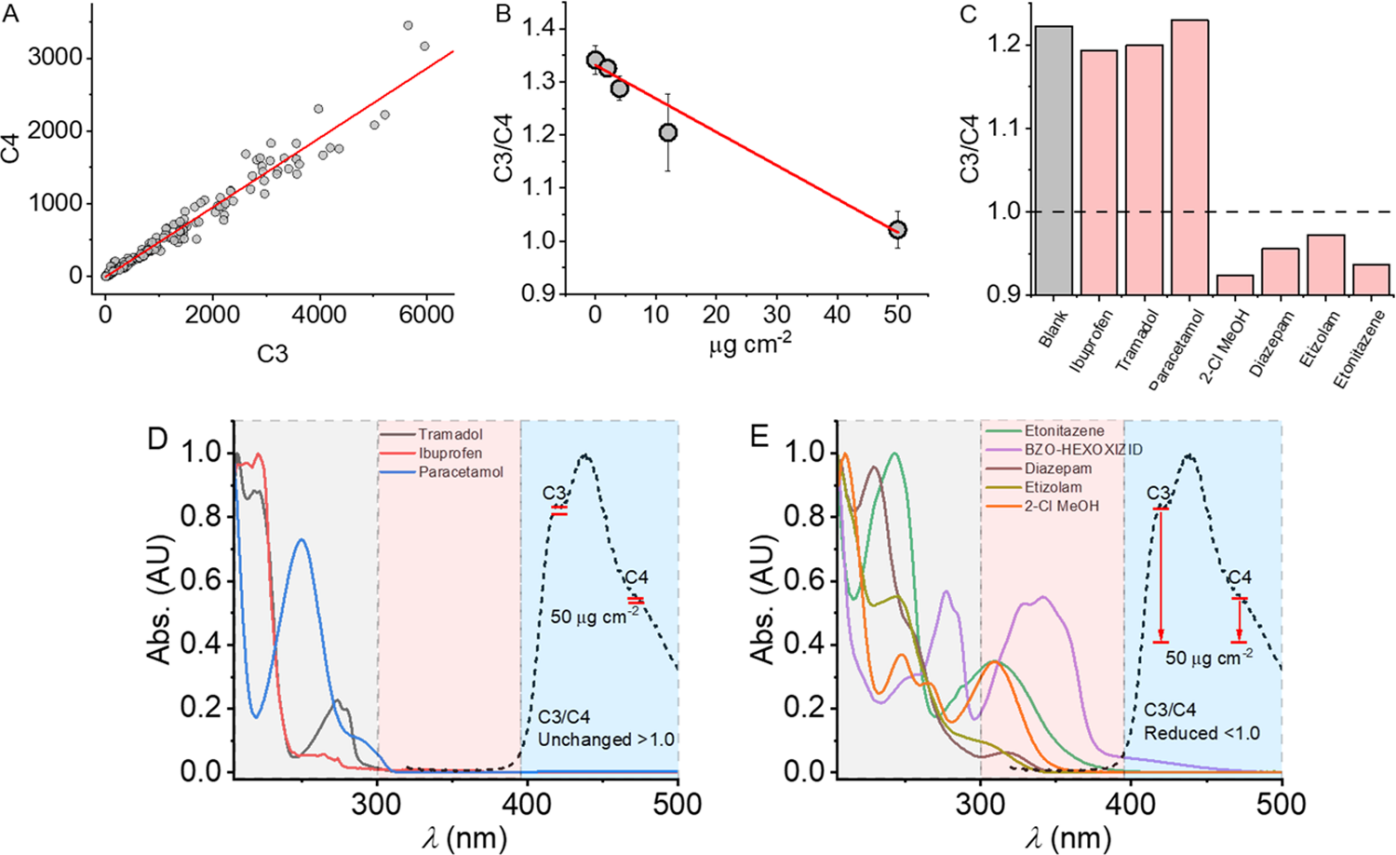
Identification of a range of illegal drugs via quenching of C3 relative to C4, with data collected on the device described in Figure 3A–C. (A) Change in C3/C4 with respect to increasing concentration of BZO-HEXOXIZID, BZO-POXIZID, and 5F-BZO-POXIZID (average and standard deviation shown). The solid red line is the fit to a simple linear function and is to aid the eye only. (B) C3/C4 value for a range of molecules (E) at 50 μg cm^–2^ on white paper. (C, D) Absorption spectra (solid lines) for specific molecules (E) and example emission spectrum from white paper (black dashed line; relative emission). The gray-colored region represents absorption arising primarily from benzene ring systems and other aromatic species. The red-colored region represents absorption features attributable to extended conjugated systems. The blue-colored region represents the bulk of emission putatively attributable to OBAs as discussed above. The red solid lines indicate the average change in emission at the designated spectral features in the presence of 50 μg cm^–2^ of the analytes in the respective panel.

For a small subset of SCs, specifically, the ligands for the OXIZIDs, we find that the relative quantum yield is much lower than for the majority of SCs we have tested. Unlike other SCs, these OXIZIDs are highly absorptive in the visible region, with a peak at ∼350 nm (see discussion below; Figure 5). Given the potential overlap of the absorption peak tail with the C3/C4 spectral region (Figure 2), we were motivated to consider if the presence of these molecules might affect the magnitude of C3 and possibly C4 via FRET or a similar mechanism. Given that the C3/C4 ratio is so highly conserved among a broad range of materials (Figure 1C), we anticipated that variation in this signal might act as a proxy from the presence of the OXIZIDs. From Figure 5A, we find that as the concentration of three different OXIZID SCs (BZO-HEXOXIZID, BZO-POXIZID, and 5F-BZO-POXIZID) are increased on white paper, there is indeed a decrease in the C3/C4 ratio. The data show an approximately linear trend with respect to concentration, with lower C3/C4 correlated with a high analyte concentration.

If our hypothesis is valid—that analytes with absorption spectra overlapping the putative OBA emission band can be detected as a change in the OBA emission spectra—notionally other molecules might be detectable in a similar way. To that end, we have measured the change in C3/C4 on paper for a range of relevant analytes. These include those that have a simple benzene ring (paracetamol, tramadol, and ibuprofen) and those with a more complex conjugated system (diazepam, etizolam, etonitazene, and a mimic of “tail-less” SCs, compound 1; Figure S5). Figure 5B shows the resultant data. We find that only those analytes with measurable absorption peaks at ∼300–400 nm give a meaningful decrease in the C3/C4 value. The absorption spectra for these compounds are shown in Figure 5C,5D. We note that these absorption spectra are not collected on the described device but using a benchtop absorption spectrometer. Moreover, it is interesting to note that even where common absorptive compounds are added to paper (e.g., printed/pen ink; Figure 2C), we do not observe a similar shift in the C3/C4 ratio. Given the values reported in Figure 2C and our data in Figure 5B, we anticipate that using this approach has a useful limit of detection of ∼50 μg cm^–2^, where C3/C4 < ∼1.0 (at least with our specific optical geometry and setup; Figure 3).

Figure 5D,E summarizes the rationale for our findings; analytes that affect the OBA emission can be detected as a simple shift in the C3/C4 value and that these correlate with many drugs of abuse and not other common drugs. That is, the detection setup shown in Figure 3 is capable of detecting not only SCs but also a very large range of illicit substances present on paper, without false positives arising from generic aromatic (benzene-based) moieties. Given that the detection is based on shifts in putative OBA emission, we anticipate that the detection will be similarly possible on fabric or other materials treated with OBAs.

## Conclusions

SCs are a critical concern in the U.K. prison system and in many prisons globally. Indeed, a recent study found that SCs were linked to nearly half of male non-natural deaths in prisons in England and Wales.^30^ The mass per dose is so low (milligrams) that SCs can be effectively smuggled by adsorbing onto an innocuous physical matrix, such as paper. Similarly, benzodiazepines have grown dramatically in their use in prisons, overtaking SCs as the major NPS in some cases. Detecting these drugs on complex matrices is imperative to stemming the flow into prisons and affecting the revenue stream that funds organized crime.

In this study, we have shown that many materials (paper, fabric, herb) give a consistent emission spectrum when excited with a ultraviolet (UV) source (265 nm). The spectra include an optically silent region in which SCs emit. In addition, the magnitude of the background in this region can be predicted with a high degree of accuracy based on the intensity of the reflected excitation light alone. This enables assignment of a background signal based on the absorption of the irradiation light, giving the ability to fine-tune the detection of SCs depending on the desired specificity or false positive rate required. Finally, emission arising from putative OBA fluorescence is remarkably consistent, and variation of this spectral feature can be used to detect the presence of low-quantum-yield SCs, such as OXIZIDs, and other more complex cross-conjugated compounds, including benzodiazepines.

We demonstrate that these advances can be implemented in a low-cost hand-held device with essentially instant detection. We note the future potential for enhanced chemometric approaches with the same hardware solution including the potential for machine learning to discriminate complex signals. The device will find immediate utility in relevant operational settings that include prisons but also for border security and within community programs to decrease the flow of SCs. Moreover, our finding that the detection modality can also be used to detect other NPS suggests a scalable application across different settings where different illegal drugs are present.

## Materials and Methods

### Sample Preparation

100 mg of plant material or ∼1–5 cm^2^ of paper samples (depending on the amount of sample available) were extracted into 2 or 4 mL of methanol, respectively. The mixture was sonicated for 30 min in a water bath (25 °C) and then centrifuged for 1 h at 12,100*g* to remove solids. The filtrate was collected, and the pellet was discarded.

### Thin-Layer Chromatography (TLC)

A selection of sample extracts was spotted onto a wide TLC plate (Figure S6). The TLC was repeated for two solvent systems: hexane/diethyl ether, 2:1 and toluene/ditheyl ether, 9:1. TLC spots were compared against the other samples for potential matches.

### Nuclear Magnetic Resonance (NMR)

Using the assumption that the concentration of SC in each sample is approximately 1–30 mg/g plant material or 0.05–1.17 mg/cm^2^ paper sample, the assumption was made that there is approximately 1 mg of SC in 1 mL of methanol extract. The methanol was removed under reduced pressure, and the sample was redispersed in the chosen NMR solvent. NMR spectra were recorded on 500 MHz Agilent ProPulse and Bruker AVANCE III 500 MHz spectrometers with 96-position sample changers. ^1^H and ^13^C NMR data were determined at 500 MHz in CDCl_3_, DMSO-*d*_6_, and CD_3_OD unless otherwise specified, and chemical shifts are reported downfield from TMS (Figures S7–S47). Coupling constants, J, are reported in Hz. Spectra were compared to SC NMR data in the literature. Where needed, structural elucidation was completed with two-dimensional (2D) NMR correlation spectroscopy (COSY), heteronuclear single quantum coherence (HSQC), heteronuclear 2 bond correlation (H2BC), heteronuclear multiple bond correlation (HMBC), and HSQC-total correlation spectroscopy (HSQC-TOCSY).

### Chromatographic Separation and Detection (LC-MS)

LC-MS analyses (Figure S48) were performed using an Agilent QTOF 6545 with a Jetstream electrospray ionization (ESI) spray source coupled to an Agilent 1260 Infinity II Quat pump high-performance liquid chromatography (HPLC) instrument with a 1260 autosampler, a column oven compartment, and a variable wavelength detector (VWD). The MS was operated in separate injections in either positive or negative ionization mode with the gas temperature at 250 °C, the drying gas at 11 L/min, and the nebulizer gas at 35 psi (2.41 bar). The sheath gas temperature and flow were set to 300 °C and 12 L/min, respectively. The MS was calibrated using reference calibrant introduced from the independent ESI reference sprayer. The VCap, Fragmentor, and Skimmer were set to 3500, 160, and 45 V, respectively. The MS was operated in all-ion mode with 3 collision energy scan segments at 0, 20, and 40 eV. Chromatographic separation of a 5 μL sample injection was performed on an InfinityLab Poroshell 120 EC-C18 (3.0 mm × 50 mm, 2.7 μm) column using H20 (Merck, LC-MS grade) with 0.1% formic acid (FA, Fluka) v/v and acetonitrile (ACN, Sigma-Aldrich) with 0.1% FA v/v as mobile phases A and B, respectively. The column was operated at a flow rate of 0.5 mL/min at 50 °C starting with 30% mobile phase B, as follows (Table 1);

**Table 1:** LC-MS Chromatographic Separation

time (min)	mobile phase B (%)
0.0	30
0.6	30
3.0	100
5.5	100
5.6	30
7.6	30

The VWD was set to detect at 254 and 320 nm wavelengths at a frequency of 2.5 Hz. Data processing was automated in Qual 10 with molecular feature extraction set to the largest 20 compounds for [M + H]^+^, [M – H]^−^, and [M + HCOO]^−^ ions. The results were also searched against an NPS database (containing 1110 compound entries) with a forward score of 25, a reverse score of 70, and mass tolerances within 5 ppm of the reference library matches. Qualified ions had coelution scores of ≥90, retention time tolerances of ±0.10, and a minimum S/N of ≥5.00.

### Fluorescence and Absorption Spectroscopy

Fluorescence emission spectra were collected using an Edinburgh Instruments FS5 spectrophotometer. Typically, excitation and emission slit widths were set at 1.5 nm, and the measurements were thermostated to 20 °C using a Peltier. Absorption measurements were collected using an Agilent Technologies Cary 60 UV–visible (UV–vis) spectrophotometer, thermostated to 20 °C by using a Peltier. In all cases, a quartz cell was used to collect spectral data.

### Synthetic Cannabinoid Detection Device

The device design is described in the main text (Figures 3 and S2). Band-pass filters (Edmund Optics), amplified photodiodes (Scitec Instruments Ltd.), and LEDs and optical elements (Thorlabs) are as described in the main text. The 3D printed housing was produced using an Ultimaker S3 printer using ABS plastic. The circuitries for driving the LED, photodiodes, Arduino (Arduino nano RP2040), and LED ring (Adafruit) are custom-made. Sensitivity is calculated as true positives/(true positives + false negatives). Specificity is calculated as true negatives/(true negatives + false positives).

## Supporting Information Available

Movie showing the ultraportable device detection of synthetic cannabinoids on variety of physical matrices (Movie S1) (MP4)
Example FSFs for the range of “core” moieties that give rise to SC fluorescence (Figure S1); further details of device design (Figure S2); fluctuation in the signal for each recorded change with respect to time (Figure S3); fluorescence spectral data for an example SC (MDMB-4en-PINACA) on paper and in solution (Figure S4); structures of analytes shown in Figure 5 (Figure S5); TLC Data of extractions from samples ASP 1–20 (Figure S6); labeled ^1^H NMR of samples analyzed (Figures S7–S47); extracted mass spectra from LC-MS analysis (Figures S48–S70); summary results for the confirmatory analysis using the combined TLC, NMR, and LC-MS workflow (Table S1); and results of trial of 181 paper (letters, cards, etc.) samples seized from prisons highlighting the effect of changing detection threshold (Table S2) (PDF)

## Notes

The material in the manuscript is relevant to UK patent application number 2219561.4.
