# Supplementary material for: Instant Detection of Synthetic Cannabinoids on Physical Matrices, Implemented on a Low-Cost, Ultraportable Device
Source: Anal Chem. 2023 Aug 29;95(37):13829–37. doi: 10.1021/acs.analchem.3c01844 (PMC10515102; doi:10.1021/acs.analchem.3c01844)
Supplement: Supplementary file 2 — ac3c01844_si_002.pdf [file ac3c01844_si_002.pdf]

Supporting Information

Instant detection of synthetic cannabinoids on physical matrices, implemented on a low-cost, ultra-portable device.

Gyles E. Cozier,[1‡] Rachael C. Andrews,[2,3‡] Anca Frinculescu,[4,5] Ranjeet Kumar,[1] Benedict May,[1] Tom Tooth,[6] Peter Collins,[7] Andrew Costello,[8,9] Tom S. F. Haines,[10] Tom P. Freeman,[11] Ian S. Blagbrough,[1] Jennifer Scott,[12] Trevor Shine,[5] Oliver B. Sutcliffe,[8] Stephen M. Husbands,[1] Jonathan Leach,[13] Richard W. Bowman,[14]* Christopher R. Pudney[1,3,15]*

[1]Department of Life Sciences, [2]Department of Chemistry, [3]Centre for Sustainable and Circular Technologies, University of Bath, Bath BA2 7AY, UK. [4]Department of Analytical, Environmental and Forensic Sciences, King's College London, 150 Stamford Street, London SE1 9NH, UK. [5]TICTAC Communications Ltd., Room 1.159 Jenner Wing, St. George's University of London, Cranmer Terrace, London SW17 0RE, UK. [6]HMP Bristol, 19 Cambridge Road, Horfield, Bristol, BS7 8PS, UK. [7]Avon and Somerset Police, Valley Road, Bristol, BS20 8JJ, UK. [8]MANchester DRug Analysis & Knowledge Exchange (MANDRAKE), Department of Natural Sciences, Manchester Metropolitan University, Manchester, M1 5GD. [9]Greater Manchester Police, Openshaw Complex, Lawton Street, Openshaw, Manchester M11 2NS [10]Department of Computer Science, [11]Department of Psychology, University of Bath, Bath, BA2 7AY, UK. [12]Bristol Medical School, University of Bristol, Bristol, BS8 2PS, UK. [13]Institute of Chemical Sciences, Heriot-Watt University, Edinburgh, EH14 4AS, UK. [14]School of Physics and Astronomy, University of Glasgow, Glasgow, G12 8QQ, UK. [15]Centre for Therapeutic Innovation, University of Bath, Bath BA2 7AY, UK.

Corresponding Author * richard.bowman@glasgow.ac.uk, c.r.pudney@bath.ac.uk

Table of Contents

Movies S4

Movie S1. Ultra-portable device detection of synthetic cannabinoids on variety of physical matrices. S4

Figures S5

Figure S1. Example FSFs for the range of 'core' moieties that give rise to SC fluorescence. S5

Figure S2. Further details of device design. S6

Figure S3. Fluctuation in signal for each recorded change with respect to time. S7

Figure S4. Fluorescence spectral data for an example SC (MDMB-4en-PINACA) on paper and in solution (MeOH). S8

Figure S5. Structures of analytes shown in Figure 5. S9

Figure S6. TLC Data of extractions from samples ASP 1-20. S10

Figure S7. Labelled ^{1}H NMR of ASP1 in $CDCl_3$. S11

Figure S8. Labelled ^{1}H NMR of ASP2 in $CDCl_3$. S12

Figure S9. Labelled ^{1}H NMR of ASP3 in $CDCl_3$. S13

Figure S10. Labelled ^{1}H NMR of ASP4 in $CDCl_3$. S14

Figure S11. Labelled ^{1}H NMR of ASP5 in $CDCl_3$. S15

Figure S12. Labelled ^{1}H NMR of ASP6 in $CDCl_3$. S16

Figure S13. Labelled ^{1}H NMR of ASP7 in $CDCl_3$. S17

Figure S14. Labelled ^{1}H NMR of ASP8 in $CDCl_3$. S18

Figure S15. Labelled ^{1}H NMR of ASP9 in $CDCl_3$. S19

Figure S16. Labelled ^{1}H NMR of ASP10 in $CDCl_3$. S20

Figure S17. Labelled ^{1}H NMR of ASP11 in $CDCl_3$. S21

Figure S18. Labelled ^{1}H NMR of ASP12 in $CDCl_3$. S22

Figure S19. Labelled ^{1}H NMR of ASP13 in $CDCl_3$. S23

Figure S20. Labelled ^{1}H NMR of ASP14 in $CDCl_3$. S24

Figure S21. Labelled ^{1}H NMR of ASP15 in $CDCl_3$. S25

Figure S22. Labelled ^{1}H NMR of ASP16 in $CDCl_3$. S26

Figure S23. Labelled ^{1}H NMR of ASP17 in $CDCl_3$. S27

Figure S24. Labelled ^{1}H NMR of ASP18 in $CDCl_3$. S28

Figure S25. Labelled ^{1}H NMR of ASP19 in $CDCl_3$. S29

Figure S26. Labelled ^{1}H NMR of ASP20 in $CDCl_3$. S30

Figure S27. Labelled ^{1}H NMR of ASP21 in $CDCl_3$. S31

Figure S28. Labelled ^{1}H NMR of ASP22 in $CDCl_3$. S32

Figure S29. Labelled ^{1}H NMR of ASP23 in $CDCl_3$. S33

Figure S30. Labelled ^{1}H NMR of ASP24 in $CDCl_3$. S34

Figure S31. Labelled ^{1}H NMR of ASP25 in $CDCl_3$. S35

Figure S32. Labelled ^{1}H NMR of ASP26 in $CDCl_3$. S36

Figure S33. Labelled ^{1}H NMR of ASP27 in $CDCl_3$. S37

Figure S34. Labelled ^{1}H NMR of ASP28 in $CDCl_3$. S38

Figure S35. Labelled ^{1}H NMR of ASP29 in $CDCl_3$. S39
Figure S36. Labelled ^{1}H NMR of ASP30 in $CDCl_3$. S40
Figure S37. Labelled ^{1}H NMR of ASP31 in CD_3OD. S41
Figure S38. Labelled ^{1}H NMR of ASP32 in CD_3OD. S42
Figure S39. Labelled ^{1}H NMR of ASP33 in CD_3OD. S43
Figure S40. Labelled ^{1}H NMR of ASP34 in CD_3OD. S44
Figure S41. Labelled ^{1}H NMR of ASP35 in CD_3OD. S45
Figure S42. Labelled ^{1}H NMR of ASP36 in CD_3OD. S46
Figure S43. Labelled ^{1}H NMR of ASP38 in CD_3OD. S47
Figure S44. Labelled ^{1}H NMR of ASP39 in CD_3OD. S48
Figure S45. Labelled ^{1}H NMR of SP066 in CD_3OD. S49
Figure S46. Labelled ^{1}H NMR of SP067 in CD_3OD. S50
Figure S47. Labelled ^{1}H NMR of SP068 in CD_3OD. S51
Figure S48. Extracted Mass Spectra from LC-MS Analysis for ASP2. S52
Figure S49. Extracted Mass Spectra from LC-MS Analysis for ASP3. S52
Figure S50. Extracted Mass Spectra from LC-MS Analysis for ASP4. S53
Figure S51. Extracted Mass Spectra from LC-MS Analysis for ASP5. S53
Figure S52. Extracted Mass Spectra from LC-MS Analysis for ASP6. S53
Figure S53. Extracted Mass Spectra from LC-MS Analysis for ASP8. S54
Figure S54. Extracted Mass Spectra from LC-MS Analysis for ASP11. S54
Figure S55. Extracted Mass Spectra from LC-MS Analysis for ASP12. S54
Figure S56. Extracted Mass Spectra from LC-MS Analysis for ASP13. S55
Figure S57. Extracted Mass Spectra from LC-MS Analysis for ASP14. S55
Figure S58. Extracted Mass Spectra from LC-MS Analysis for ASP15. S55
Figure S59. Extracted Mass Spectra from LC-MS Analysis for ASP16. S56
Figure S60. Extracted Mass Spectra from LC-MS Analysis for ASP17. S56
Figure S61. Extracted Mass Spectra from LC-MS Analysis for ASP18. S57
Figure S62. Extracted Mass Spectra from LC-MS Analysis for ASP19. S58
Figure S63. Extracted Mass Spectra from LC-MS Analysis for ASP22. S59
Figure S64. Extracted Mass Spectra from LC-MS Analysis for ASP23. S59
Figure S65. Extracted Mass Spectra from LC-MS Analysis for ASP24. S59
Figure S66. Extracted Mass Spectra from LC-MS Analysis for ASP25. S60
Figure S67. Extracted Mass Spectra from LC-MS Analysis for ASP26. S60
Figure S68. Extracted Mass Spectra from LC-MS Analysis for ASP28. S61
Figure S69. Extracted Mass Spectra from LC-MS Analysis for ASP29. S62
Figure S70. Extracted Mass Spectra from LC-MS Analysis for ASP30. S63
Tables S64
Table S1. Summary results for the confirmatory analysis using the combined TLC, NMR and LC-MS workflow. S64
Table S2. Results of trial of 181 paper (letters, cards etc.) samples seized from prisons highlighting the effect of changing detection threshold. S66

Movies

Movie S1. Ultra-portable device detection of synthetic cannabinoids on variety of physical matrices. See video file in online supporting information.

Figures

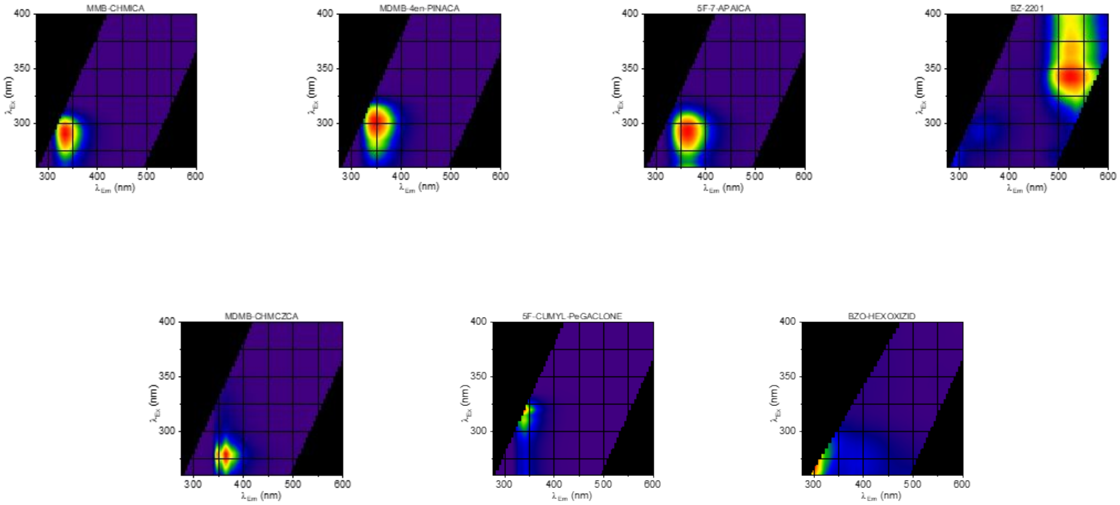

Figure S1. Example FSFs for the range of ‘core’ moieties that give rise to SC fluorescence.

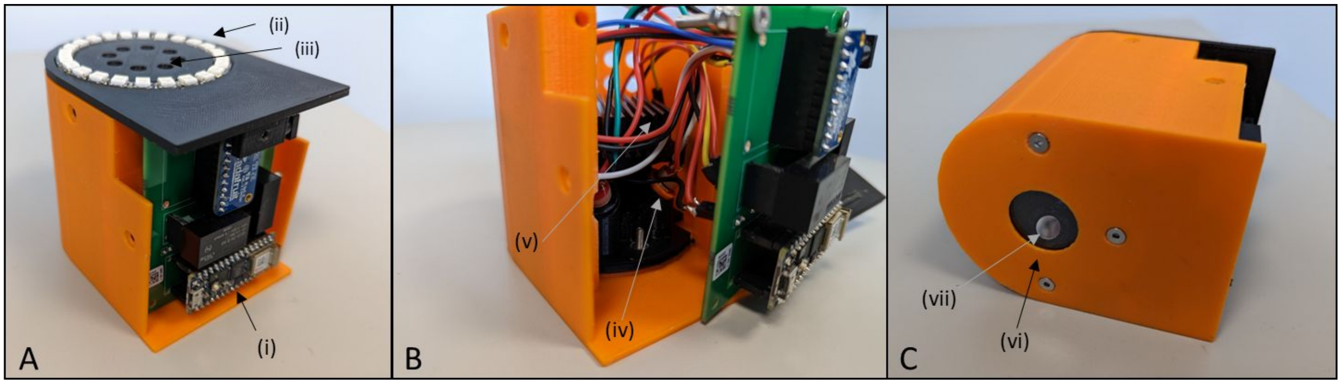

Figure S2. Further details of device design. (i) Custom PCB with power handling and conversion, LED driver, ADC and microcontroller. (ii) LED ring for reporting. (iii) ventilation for heatsink. (iv) Photodiode (one of four). (v) The heatsink. (vi) a sapphire window to protect the bandpass filters. (vii) bandpass filter (one of four).

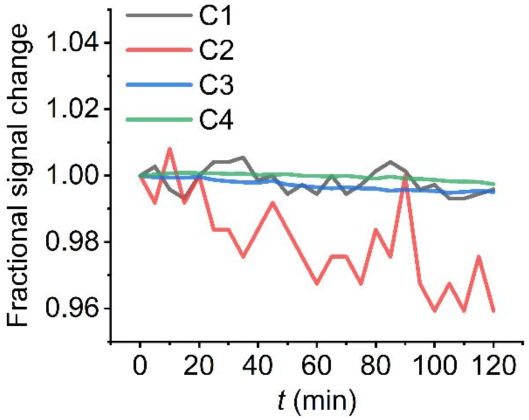

Figure S3. Fluctuation in signal for each recorded change with respect to time.

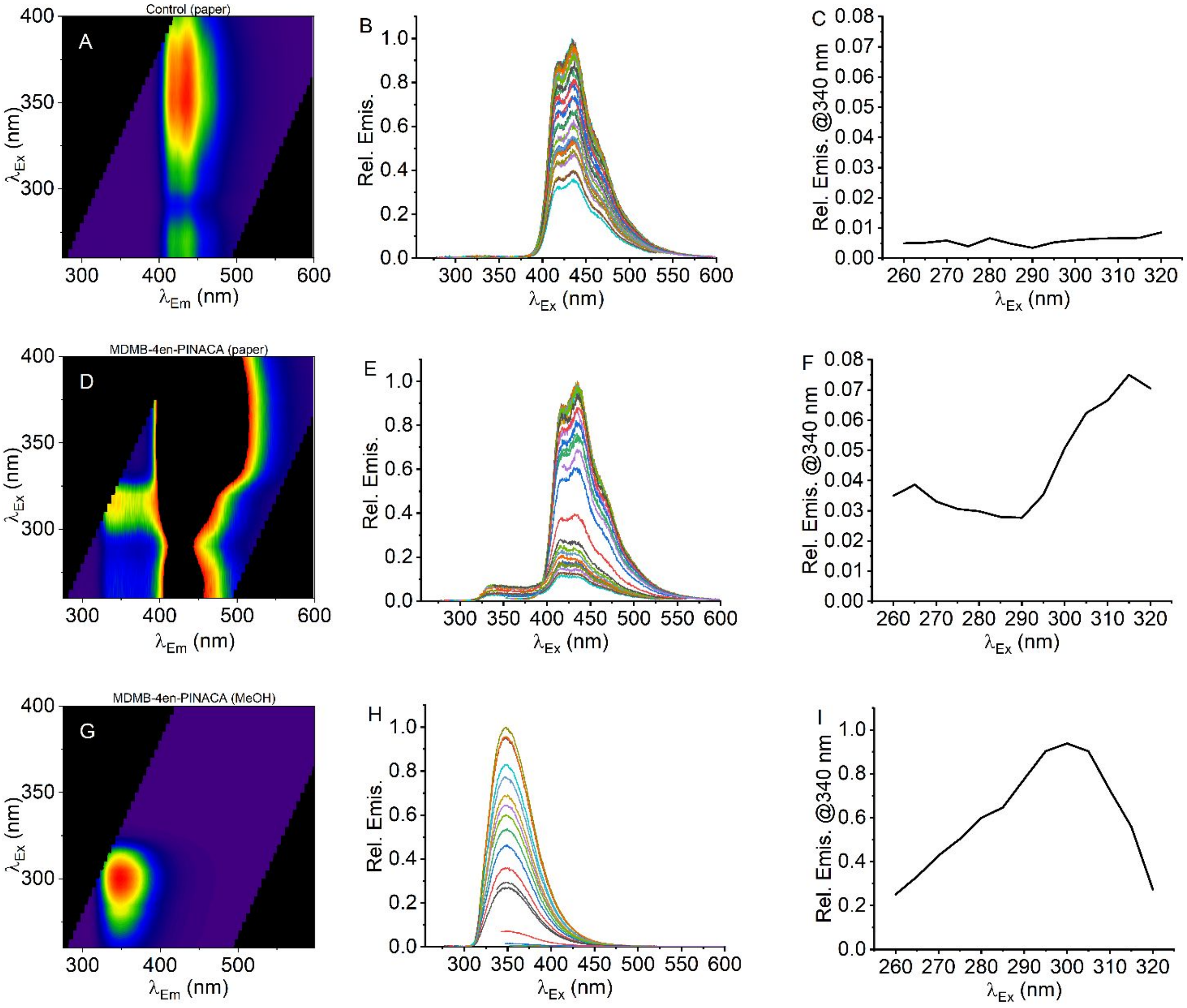

Figure S4. Fluorescence spectral data for an example SC (MDMB-4en-PINACA) on paper and in solution (MeOH). Each row shows the EEM (at left), constituent spectra (middle panel) and excitation spectrum at 340 nm emission (at right) for paper (**A-C**), paper with MDMB-4en-PINACA (**D-F**) and MDMB-4en-PINACA in MeOH (**G-I**).

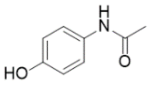
Paracetamol

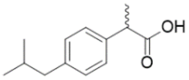
Ibuprofen

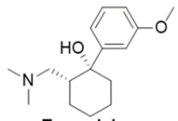
Tramadol

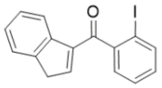
1

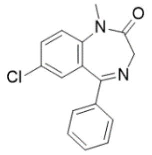
Diazepam

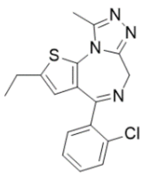
Etizolam

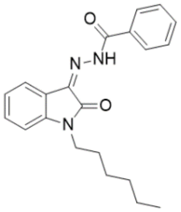
BZO-HEXOXIZID

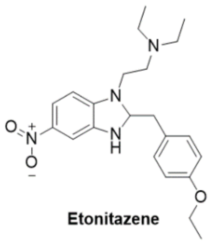
Etonitazene

Figure S5. Structures of analytes shown in Figure 5.

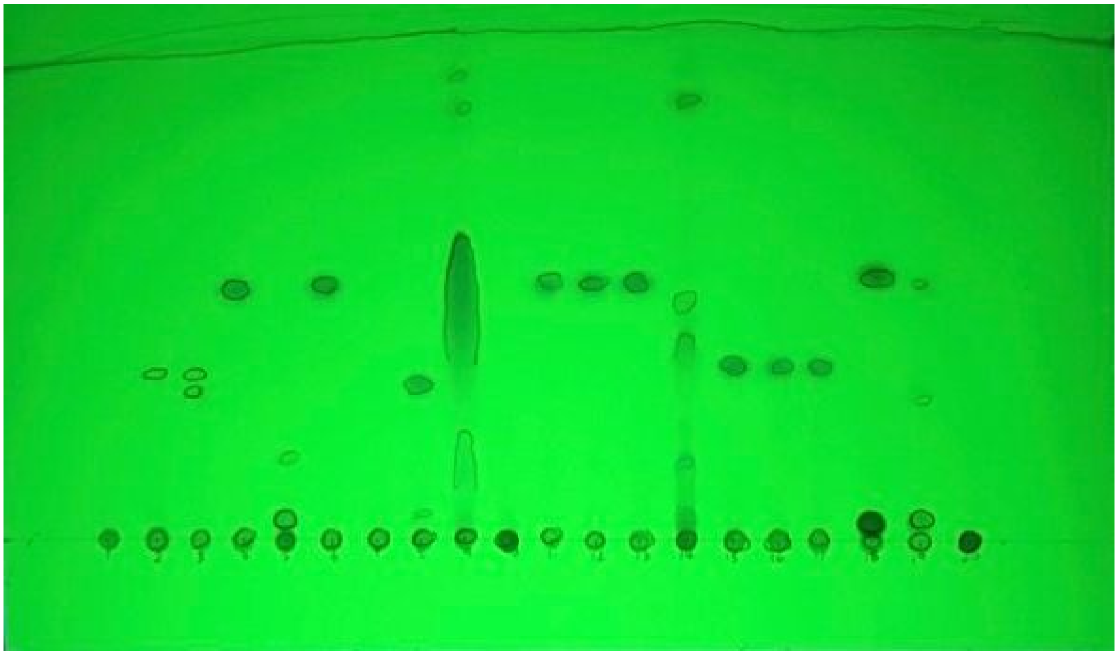

Silica TLC plate showing ASP 1-20, run in hexane : diethyl ether, 2 : 1

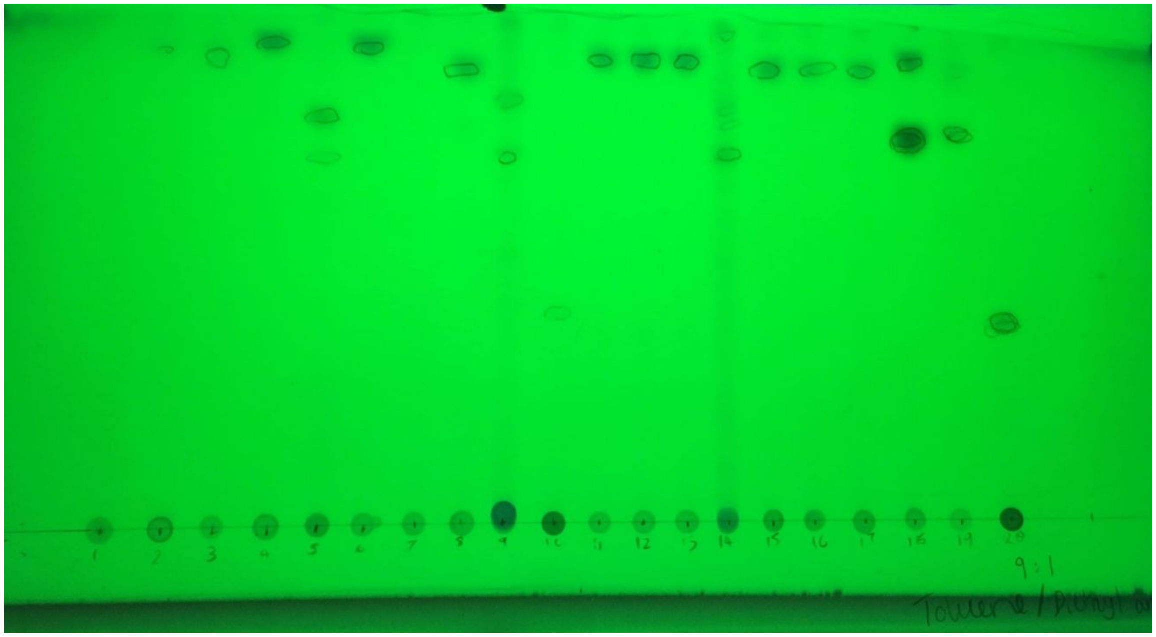

Silica TLC plate showing ASP 1-20, run in toluene : diethyl amine, 9 : 1

Figure S6. TLC Data of extractions from samples ASP 1-20.

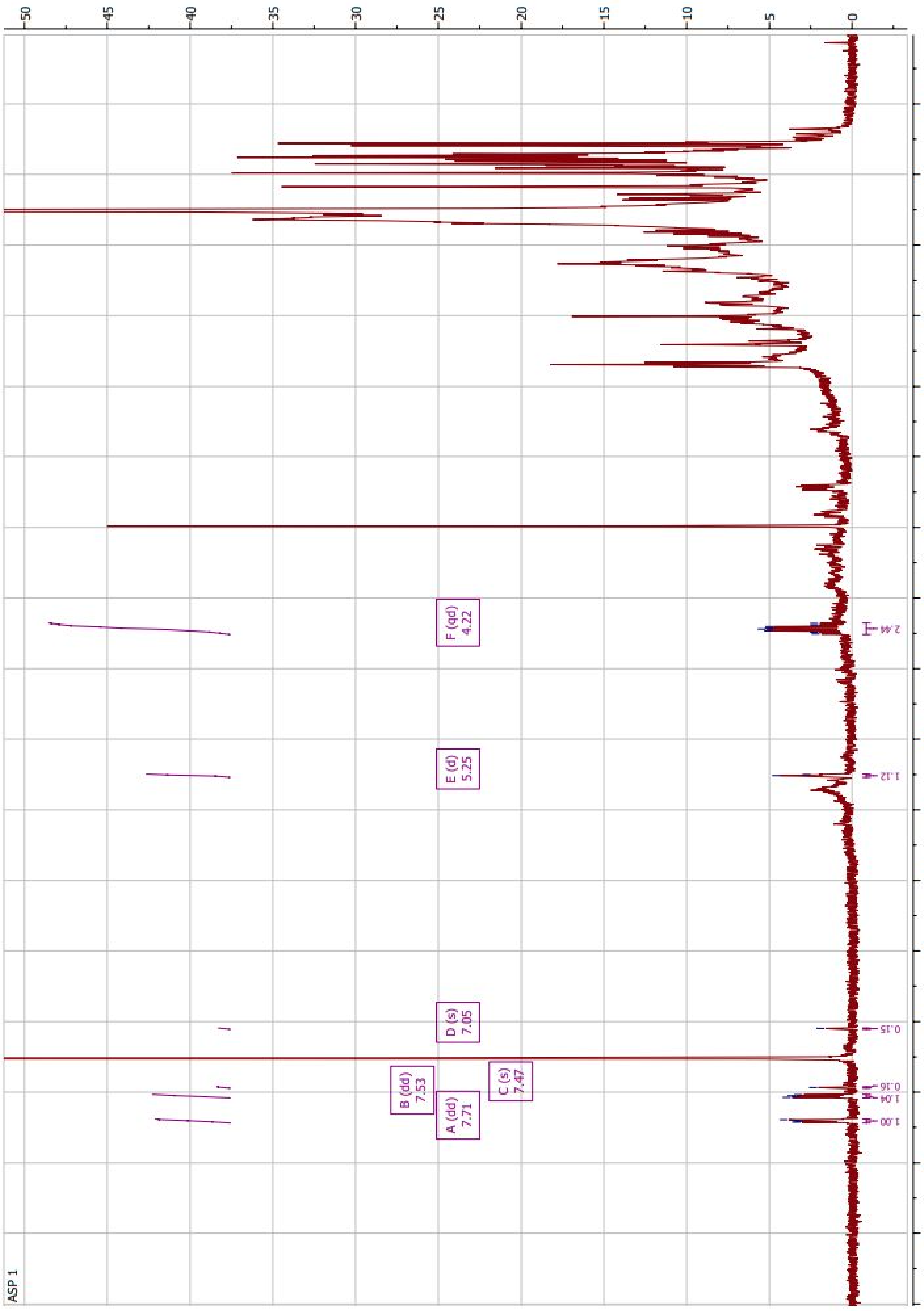

Figure S7. Labelled ^{1}H NMR of ASP1 in $CDCl_3$.

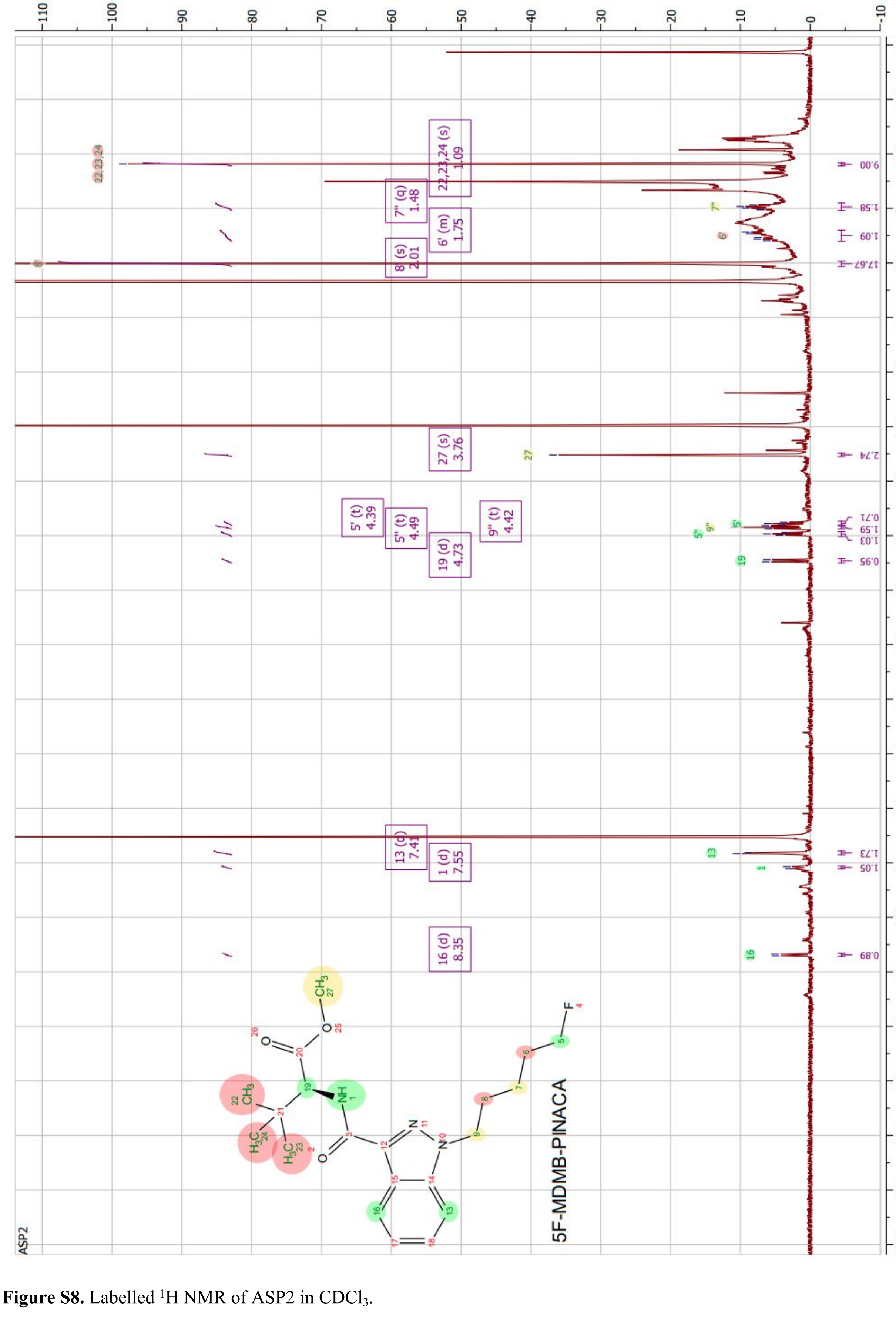

Figure S8. Labelled ^{1}H NMR of ASP2 in $CDCl_3$.

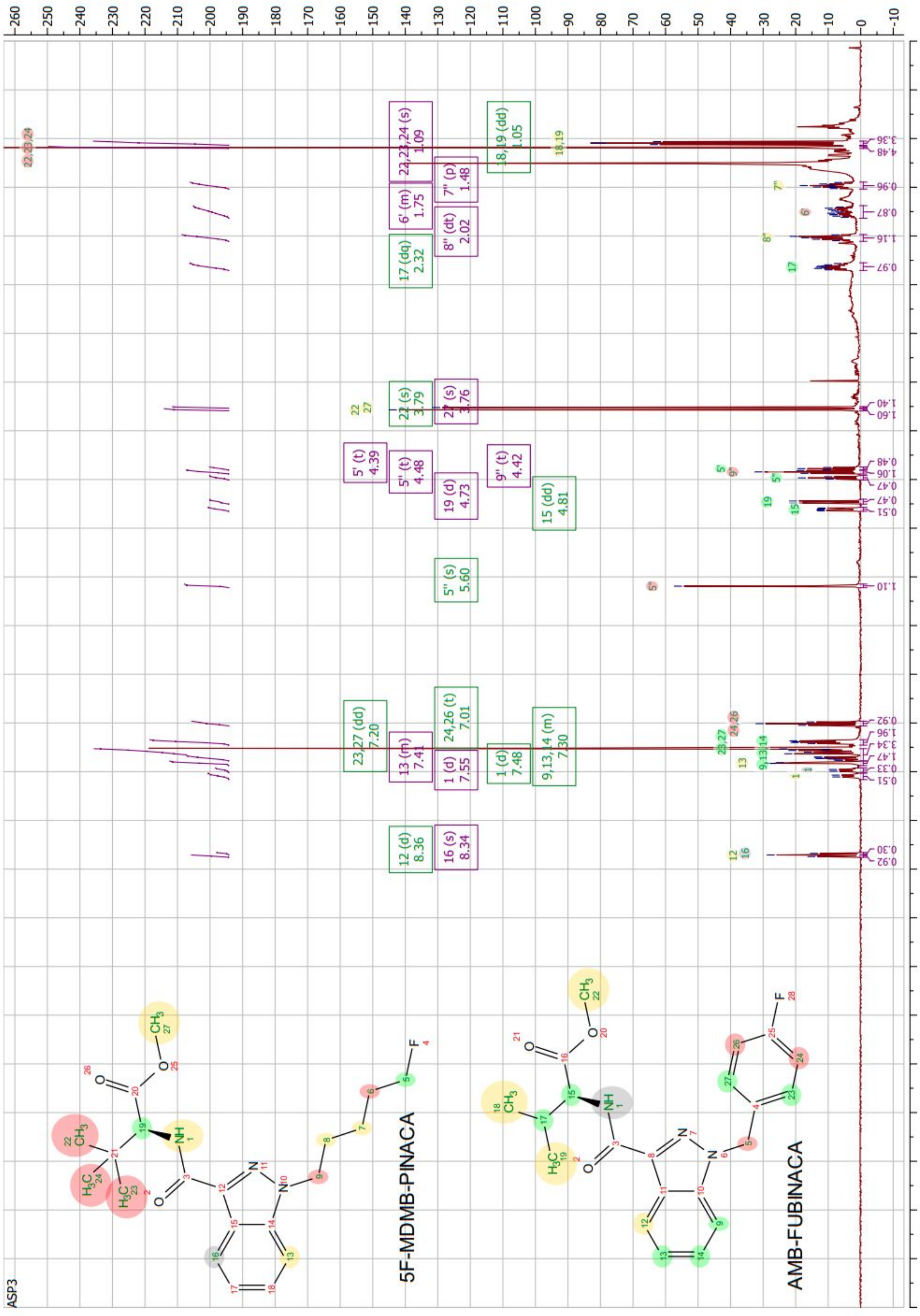

Figure S9. Labelled ^{1}H NMR of ASP3 in $CDCl_3$.

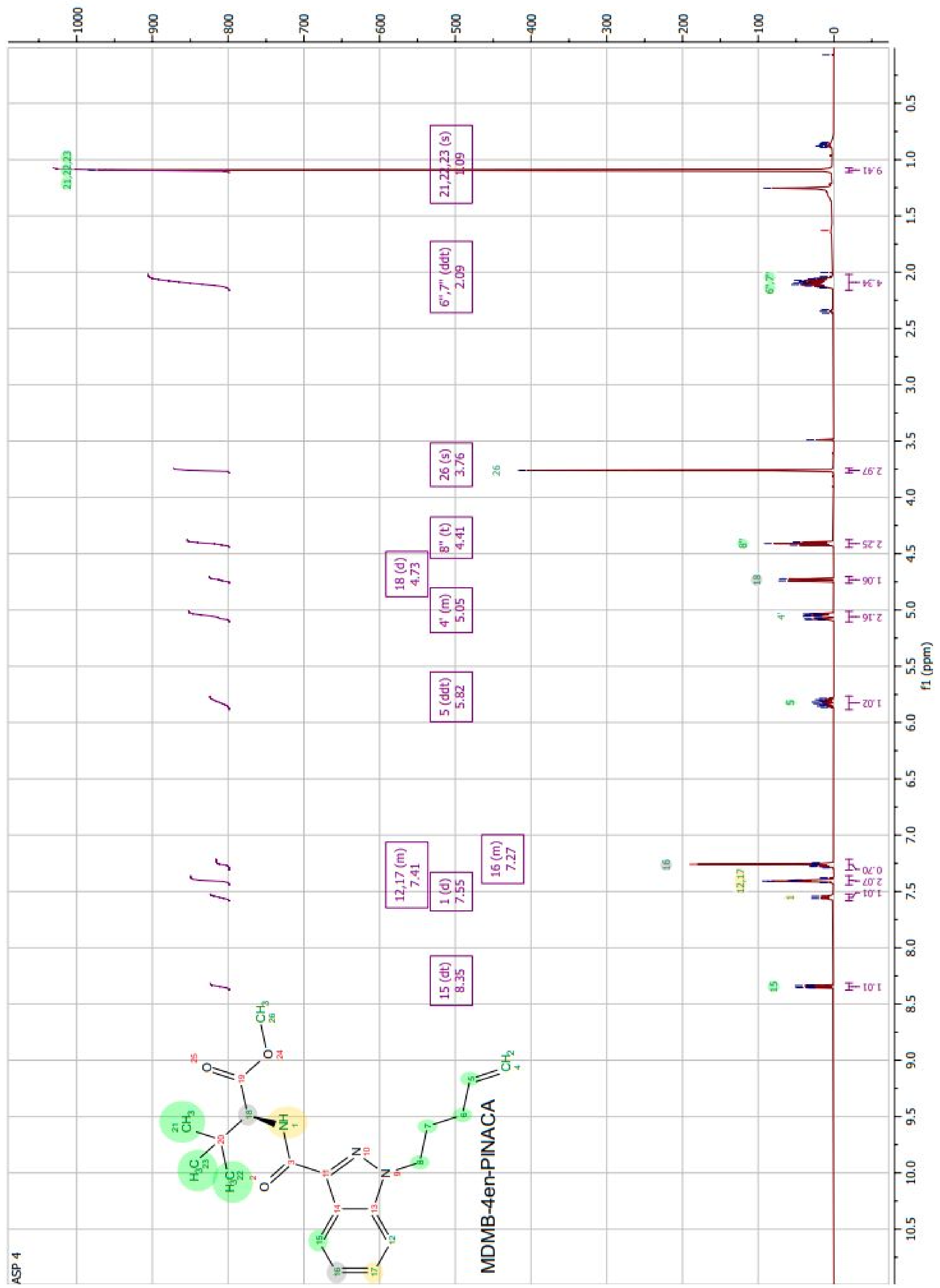

Figure S10. Labelled ^1H NMR of ASP4 in $CDCl_3$.

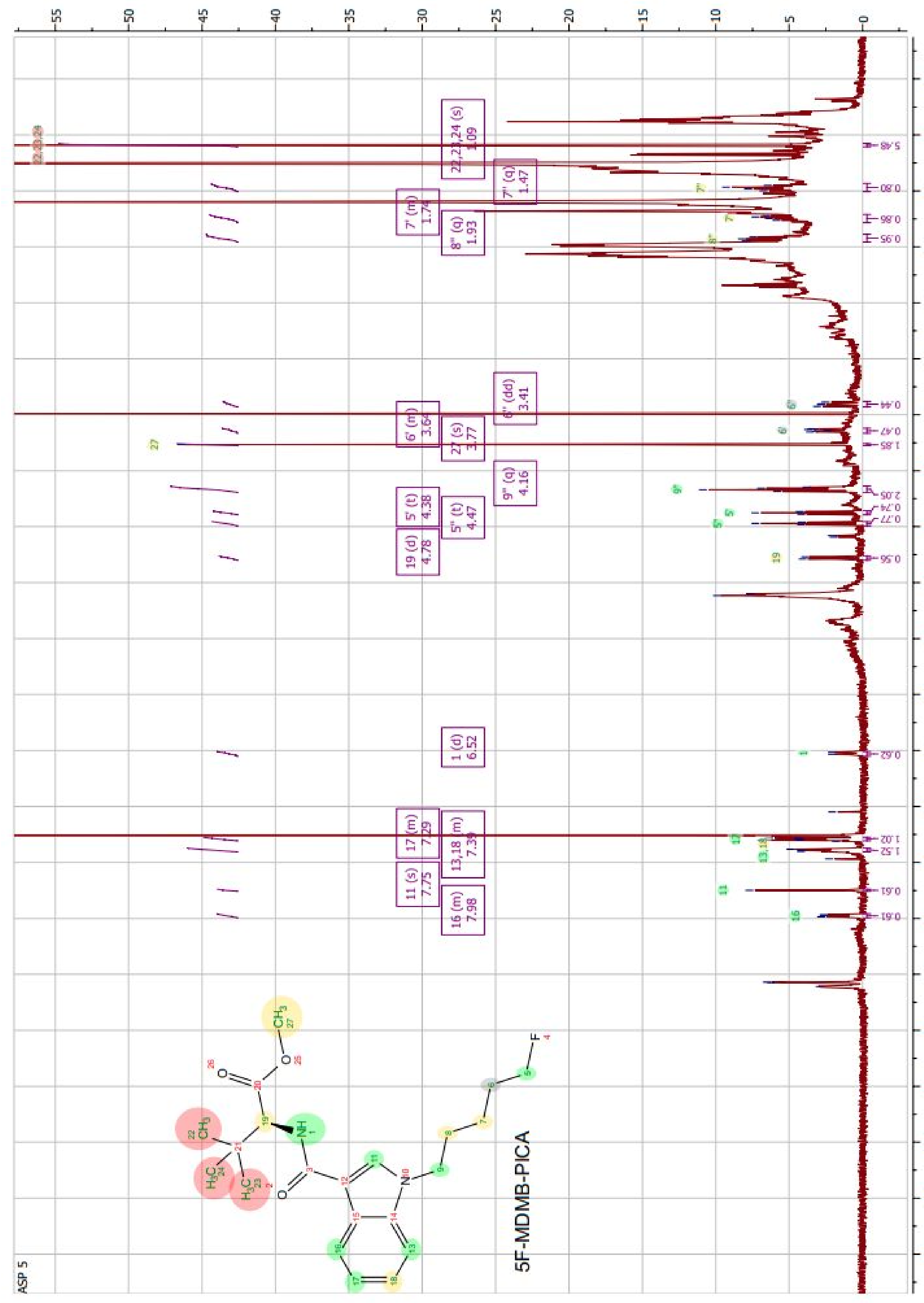

Figure S11. Labelled ^{1}H NMR of ASP5 in $CDCl_3$.

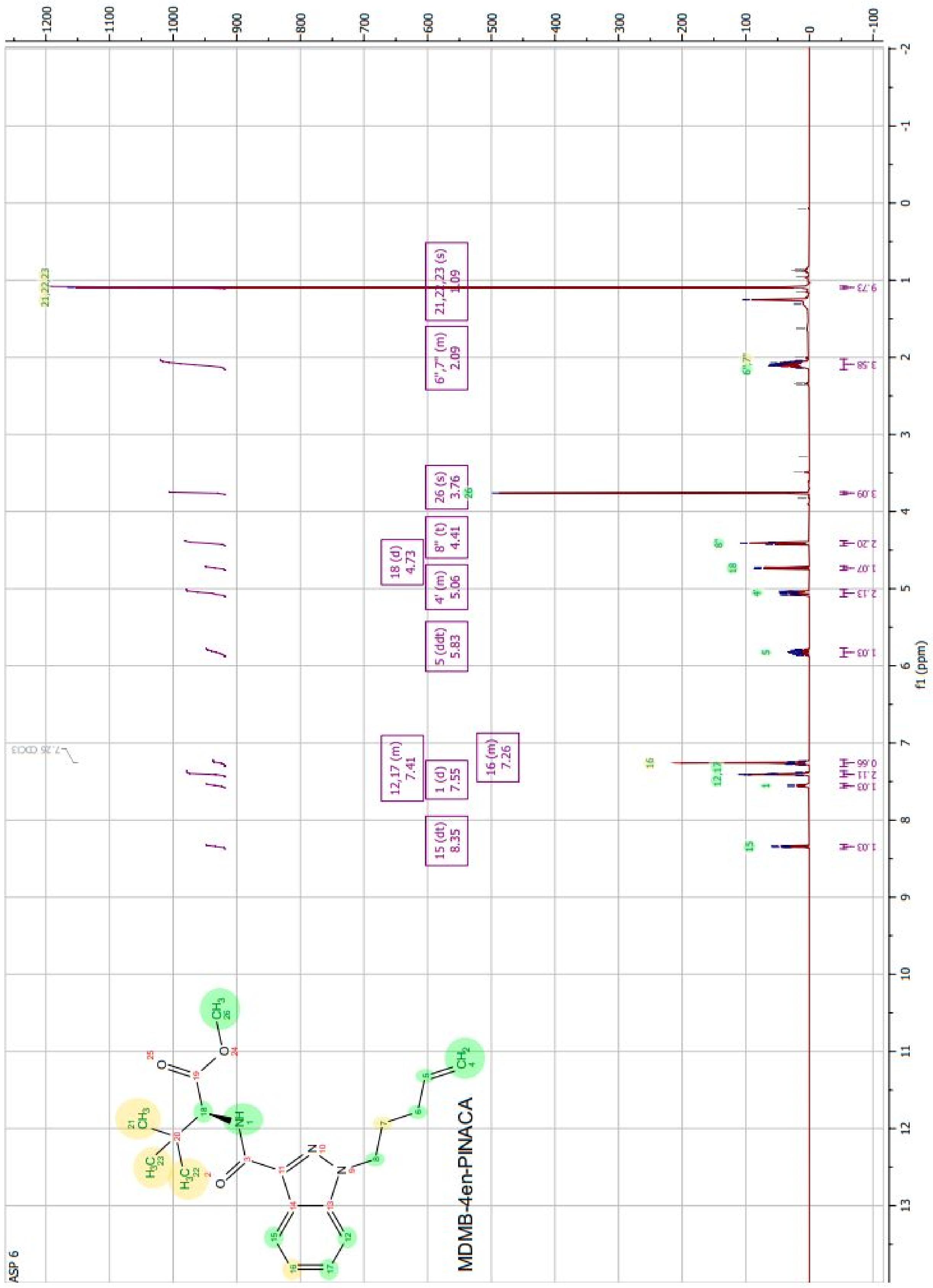

Figure S12. Labelled ^{1}H NMR of ASP6 in $CDCl_3$.

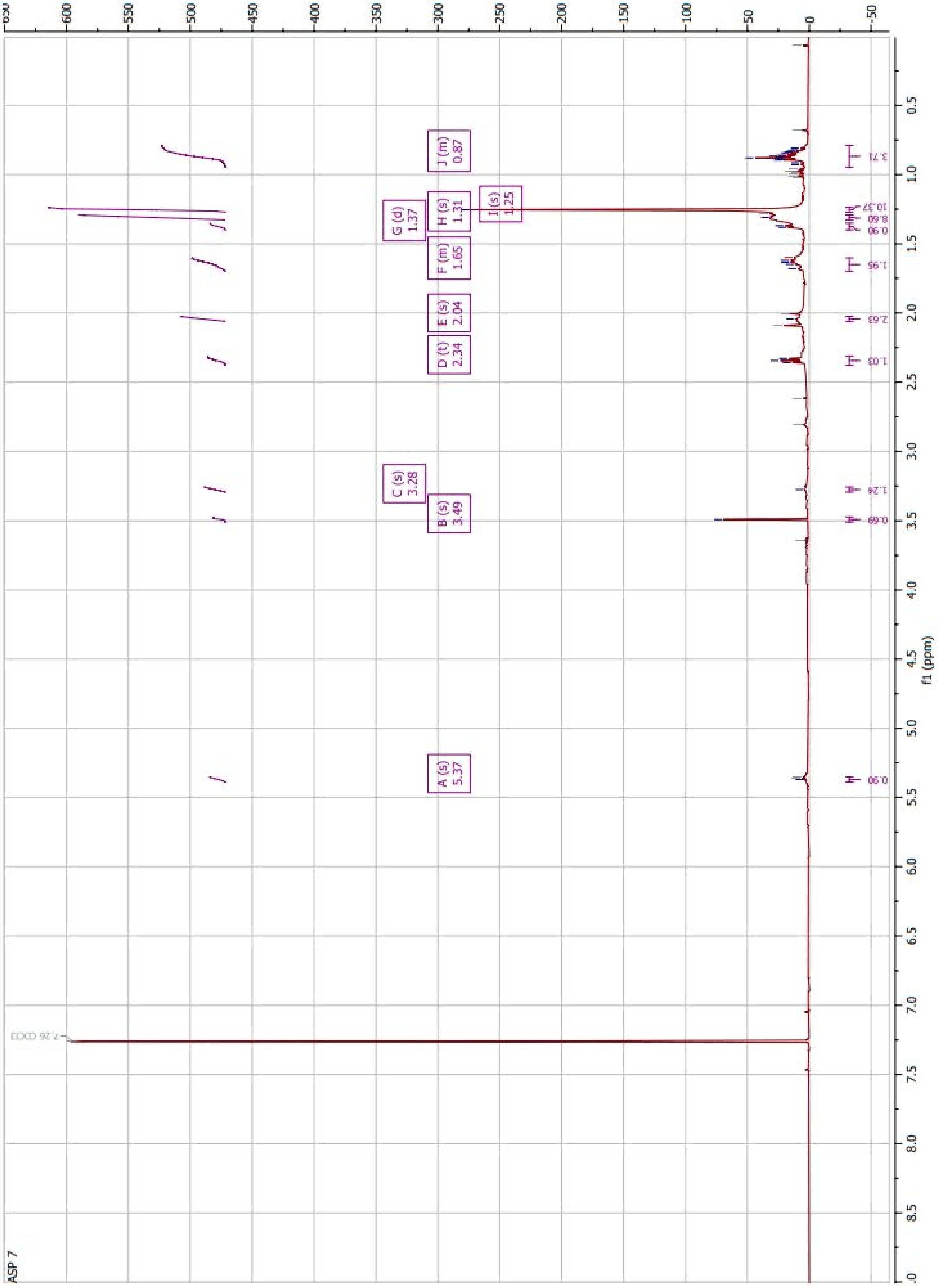

Figure S13. Labelled ^{1}H NMR of ASP7 in $CDCl_3$.

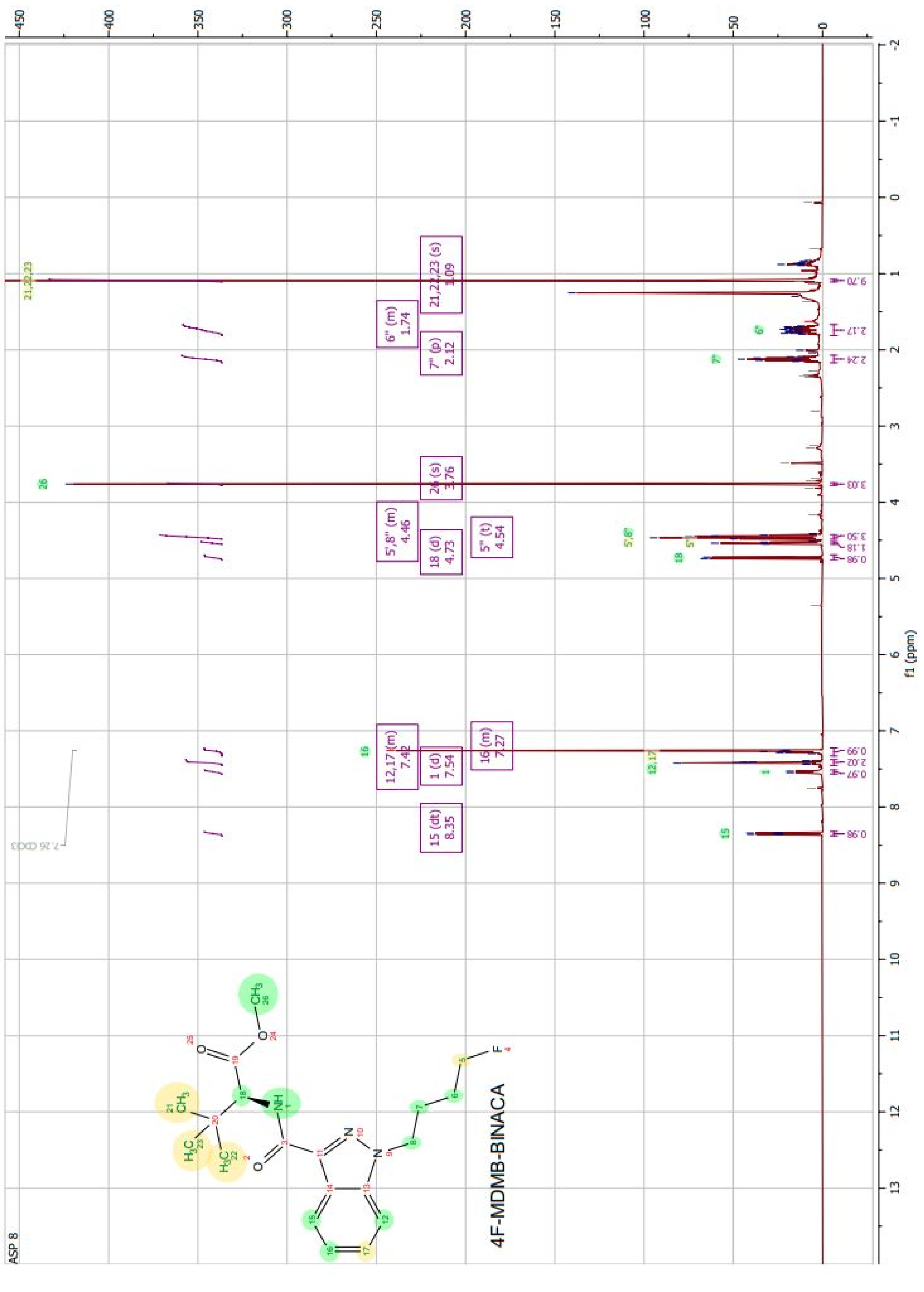

Figure S14. Labelled ^{1}H NMR of ASP8 in $CDCl_3$.

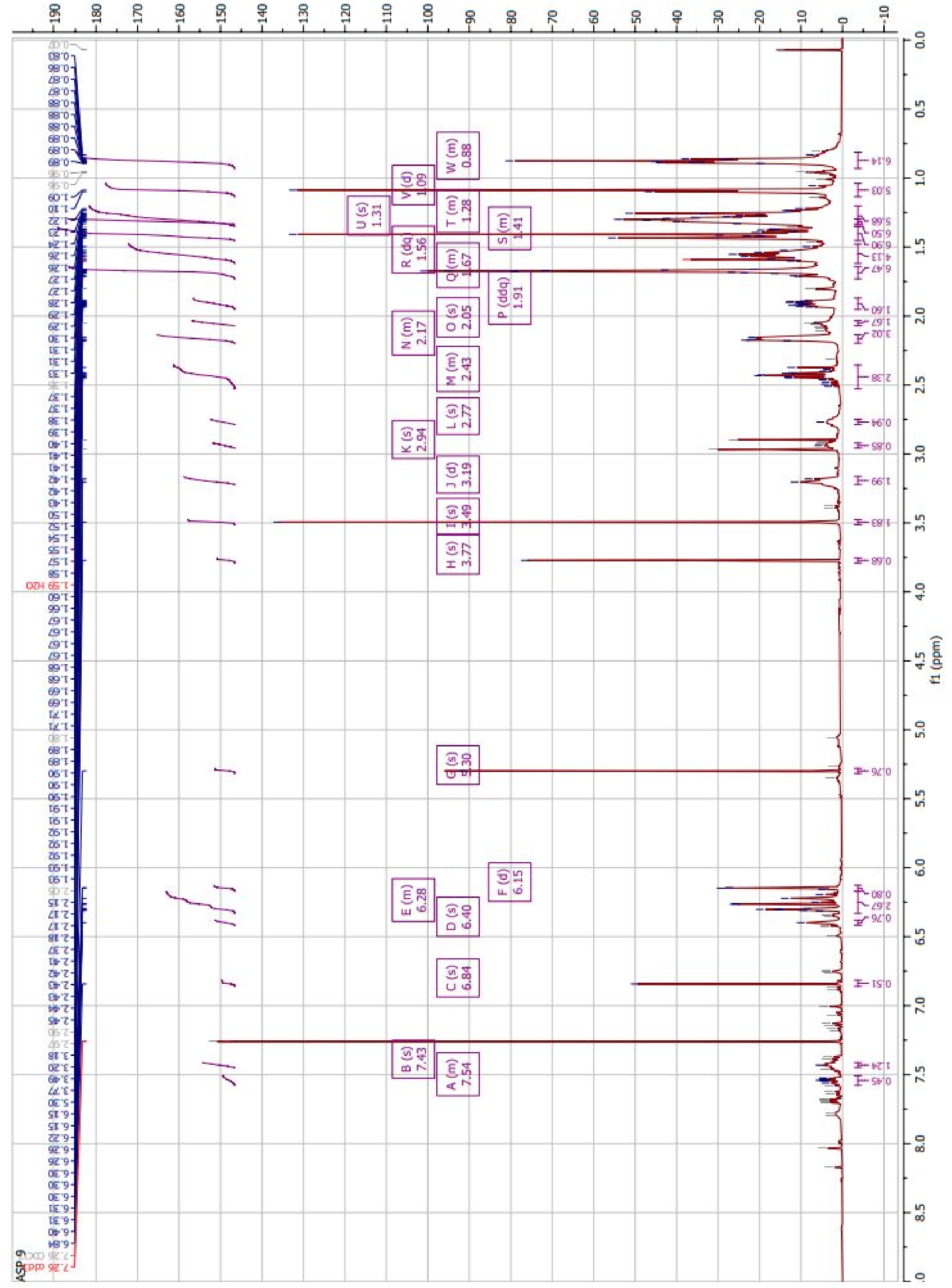

Figure S15. Labelled ^{1}H NMR of ASP9 in $CDCl_3$.

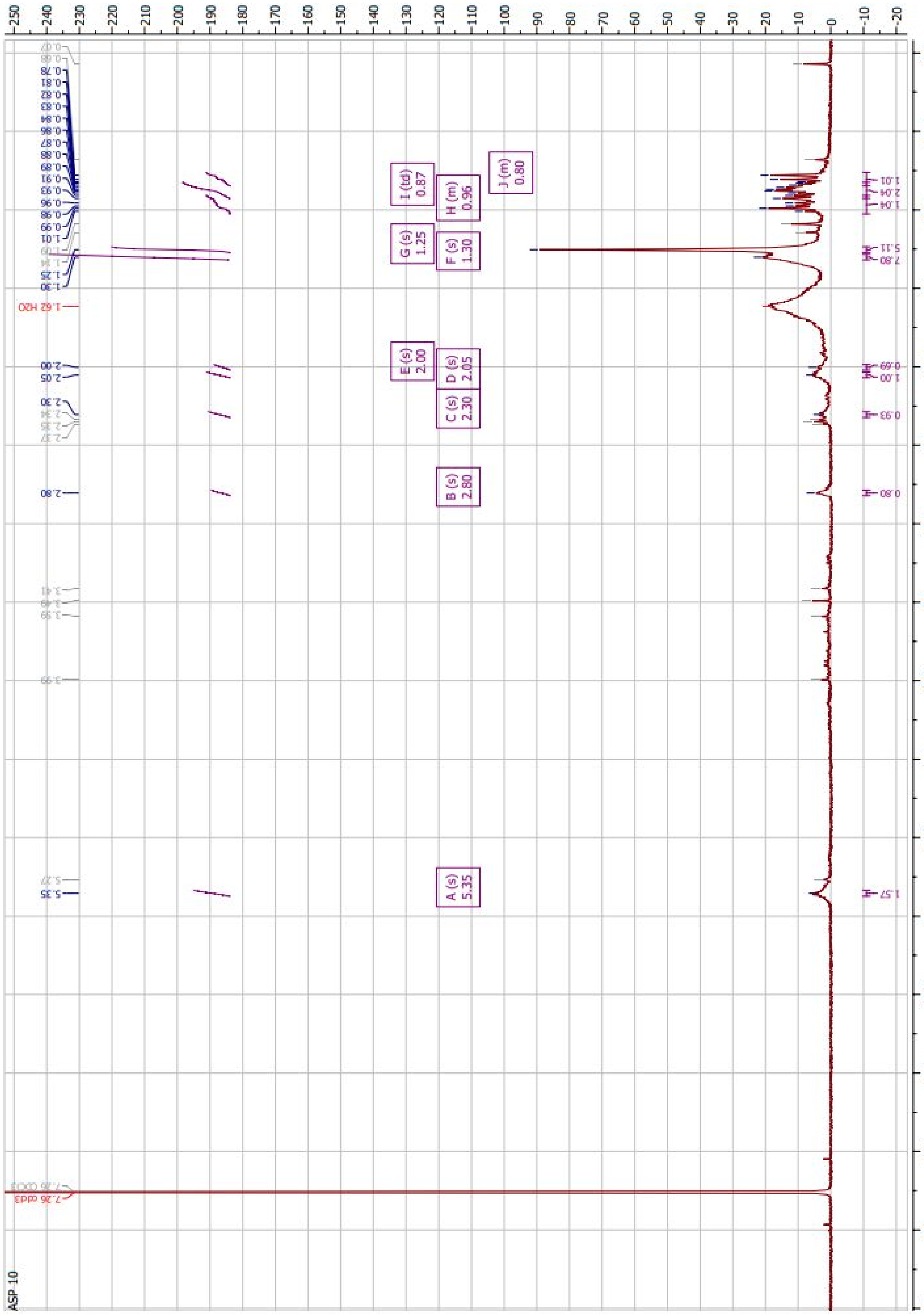

Figure S16. Labelled ^{1}H NMR of ASP10 in $CDCl_3$.

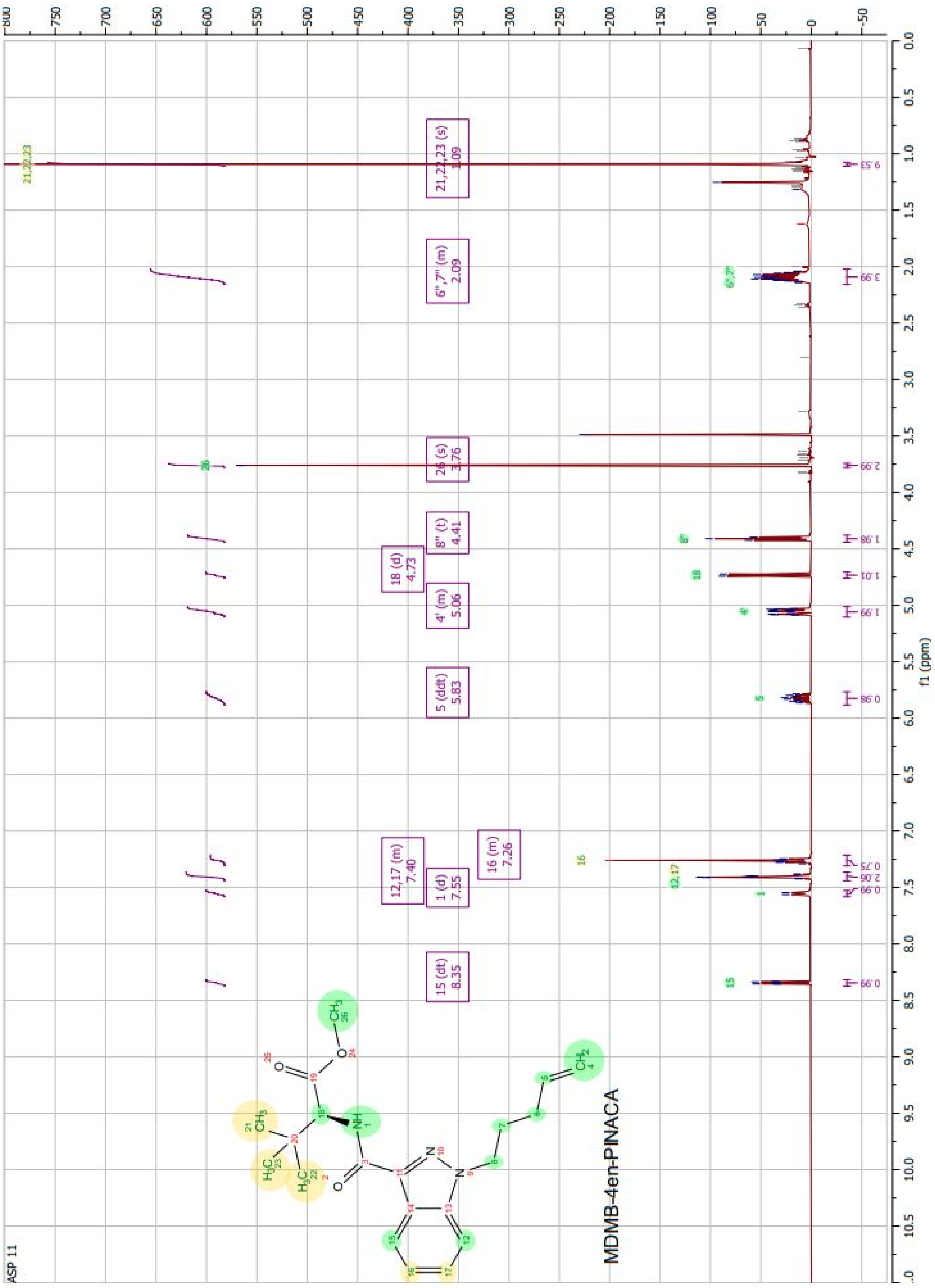

Figure S17. Labelled ^{1}H NMR of ASP11 in $CDCl_3$.

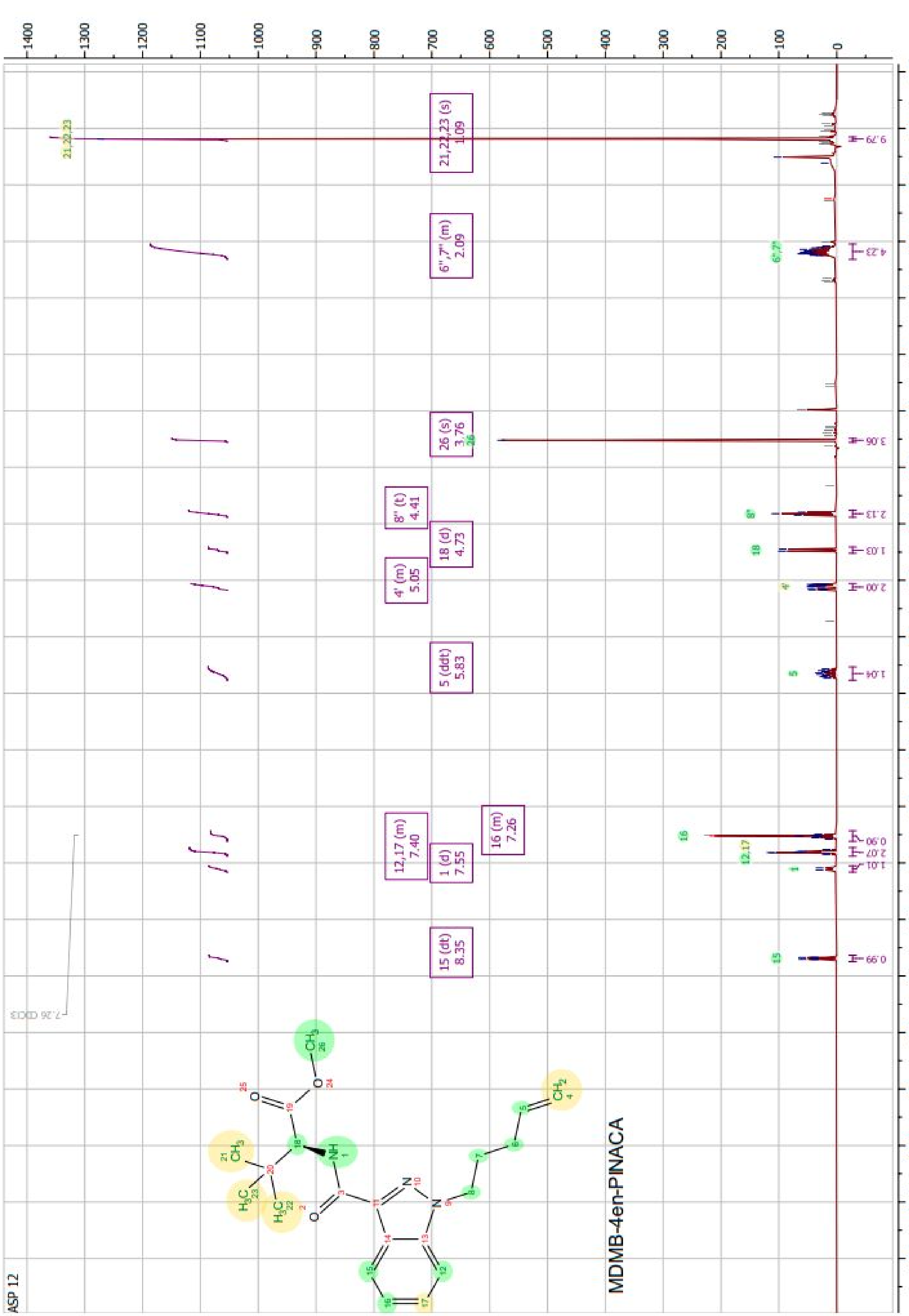

Figure S18. Labelled ^{1}H NMR of ASP12 in $CDCl_3$.

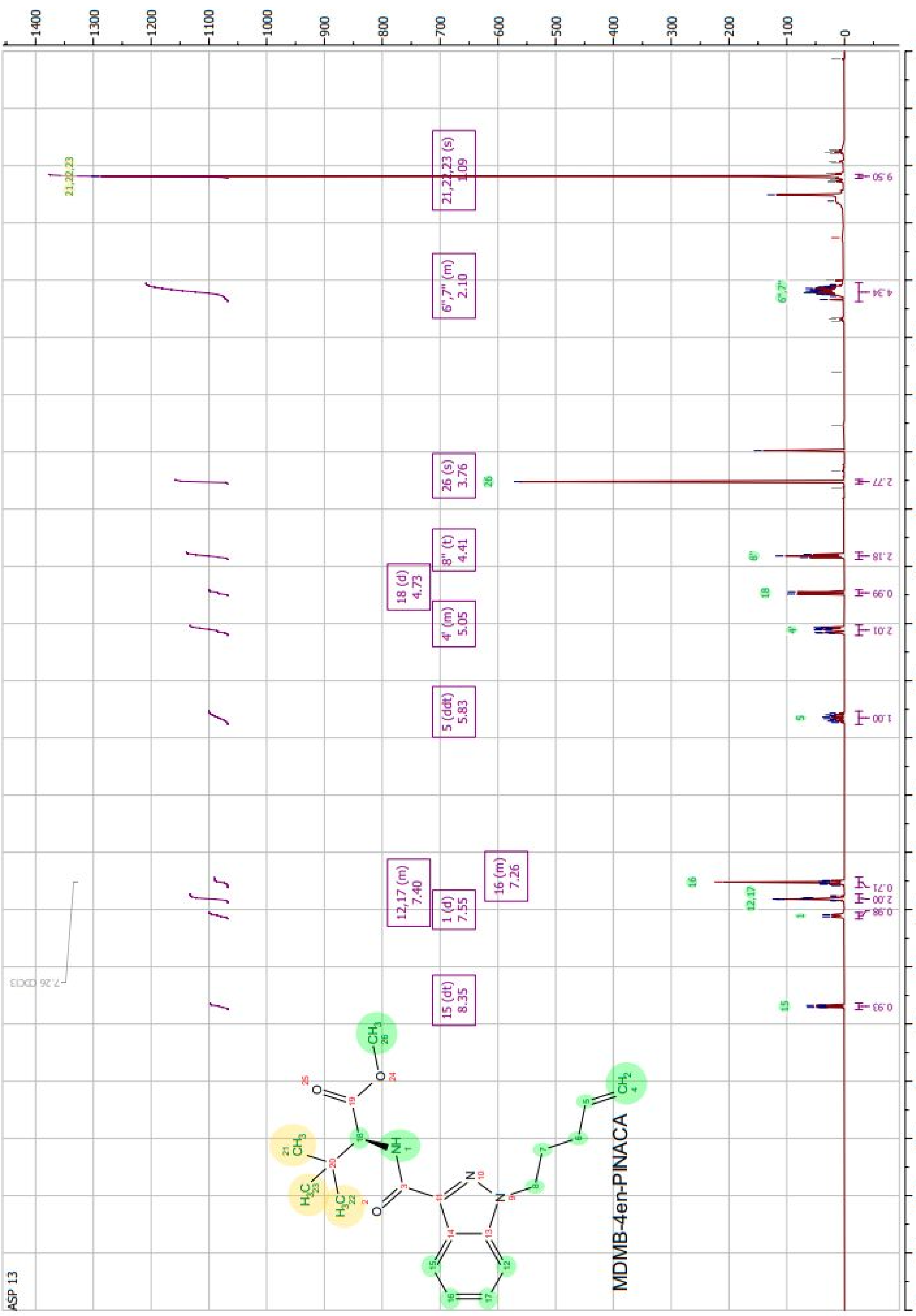

Figure S19. Labelled ^{1}H NMR of ASP13 in $CDCl_3$.

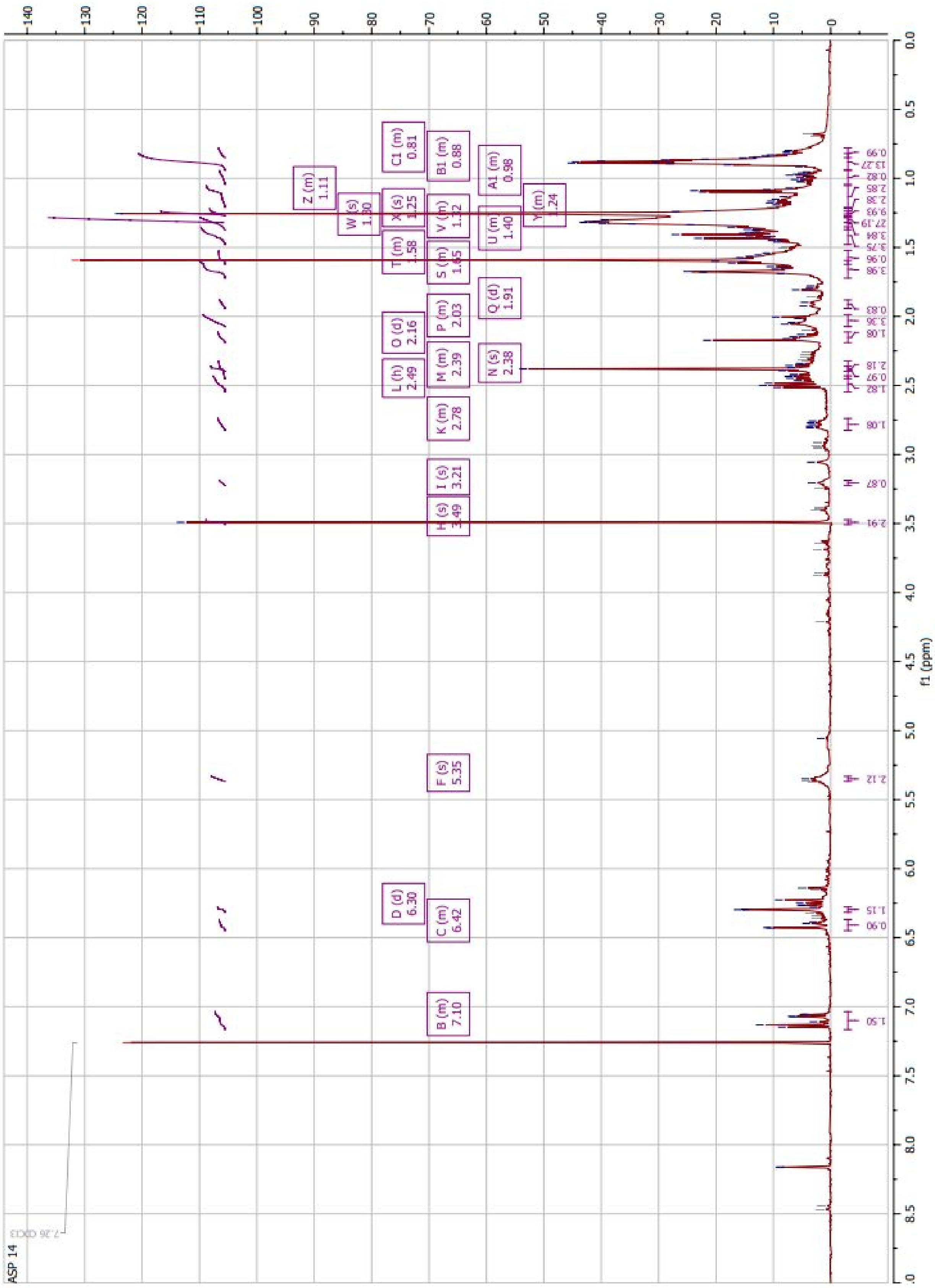

Figure S20. Labelled ^{1}H NMR of ASP14 in $CDCl_3$.

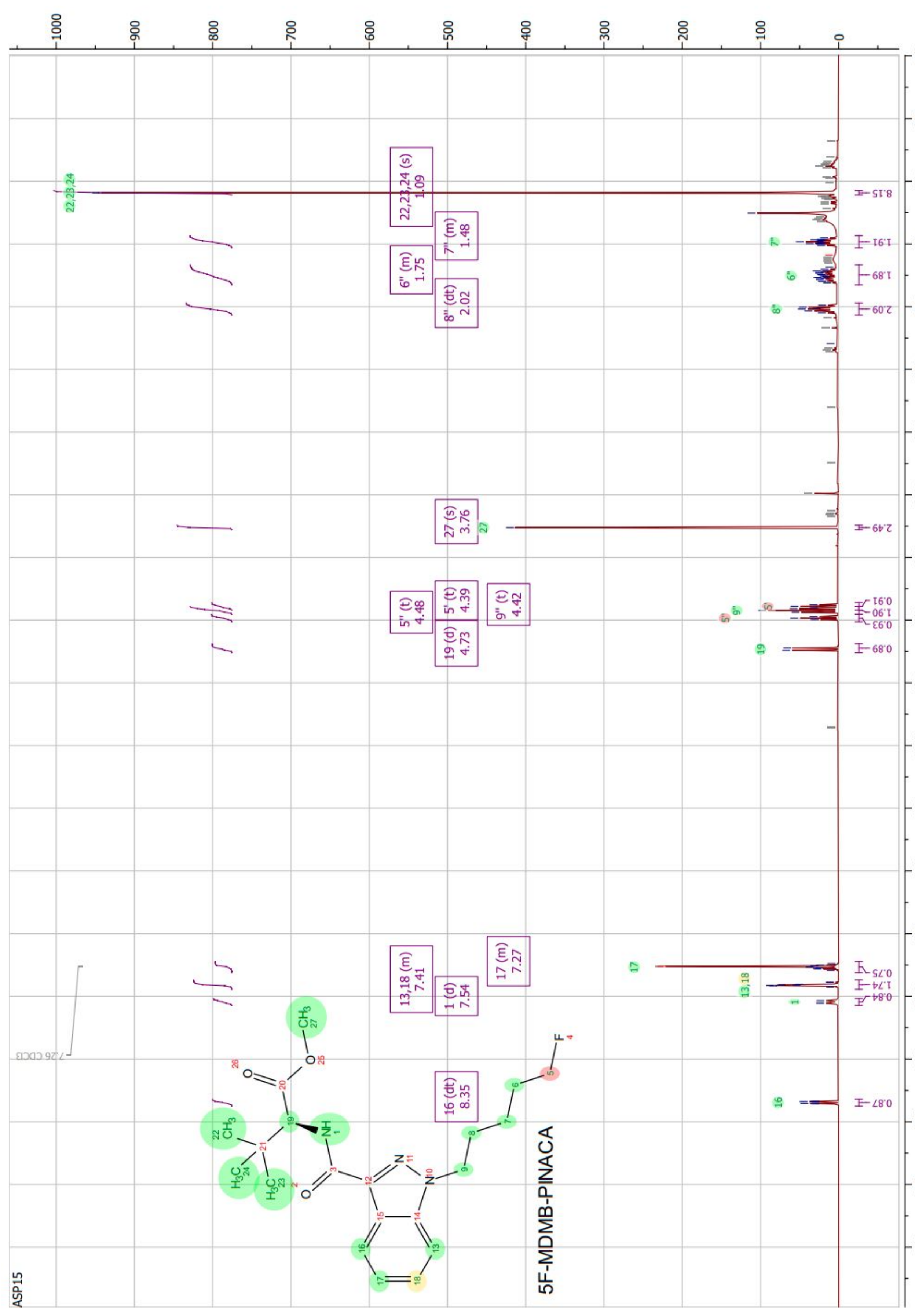

Figure S21. Labelled ^{1}H NMR of ASP15 in $CDCl_3$.

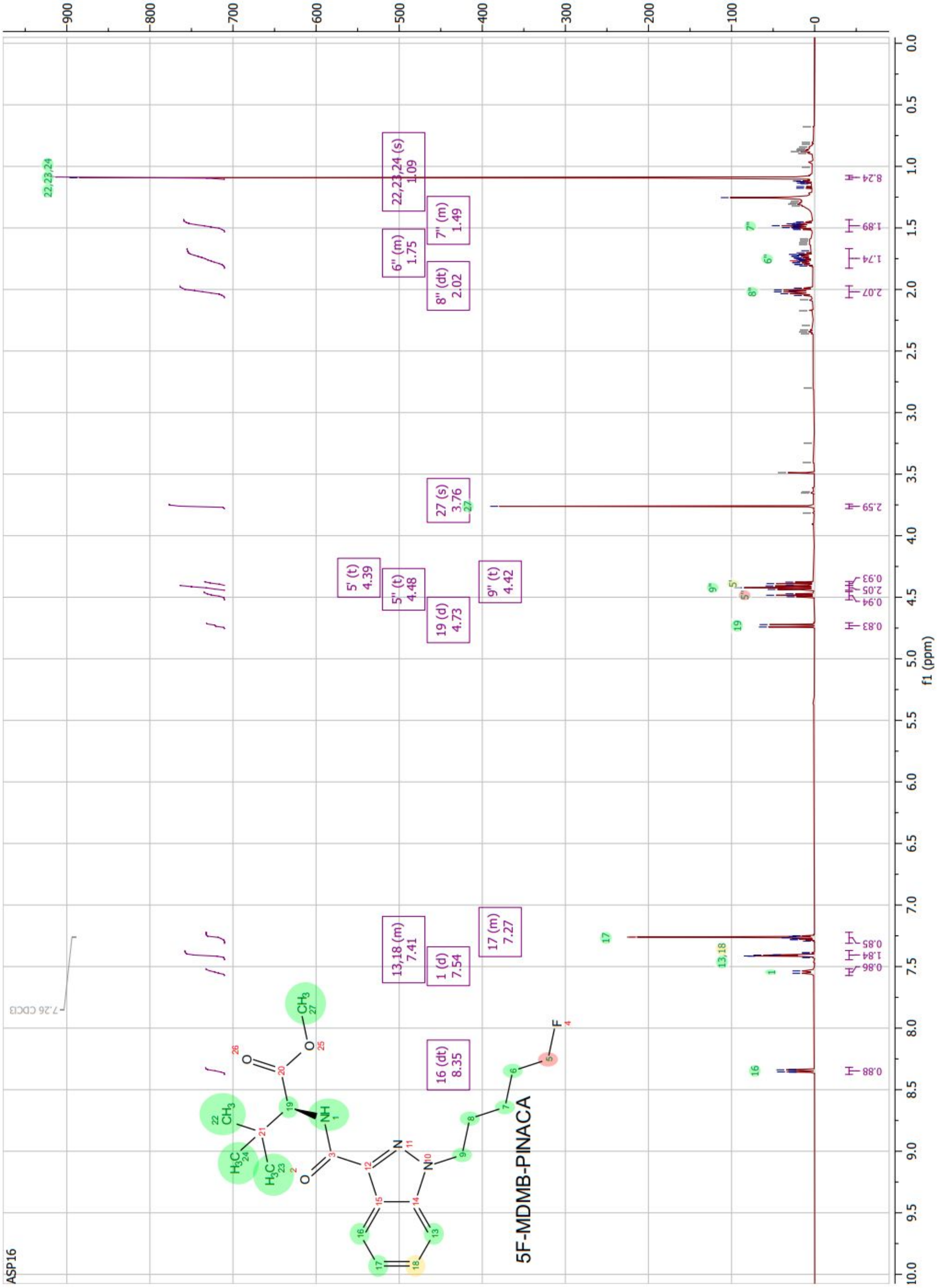

Figure S22. Labelled ^{1}H NMR of ASP16 in $CDCl_3$.

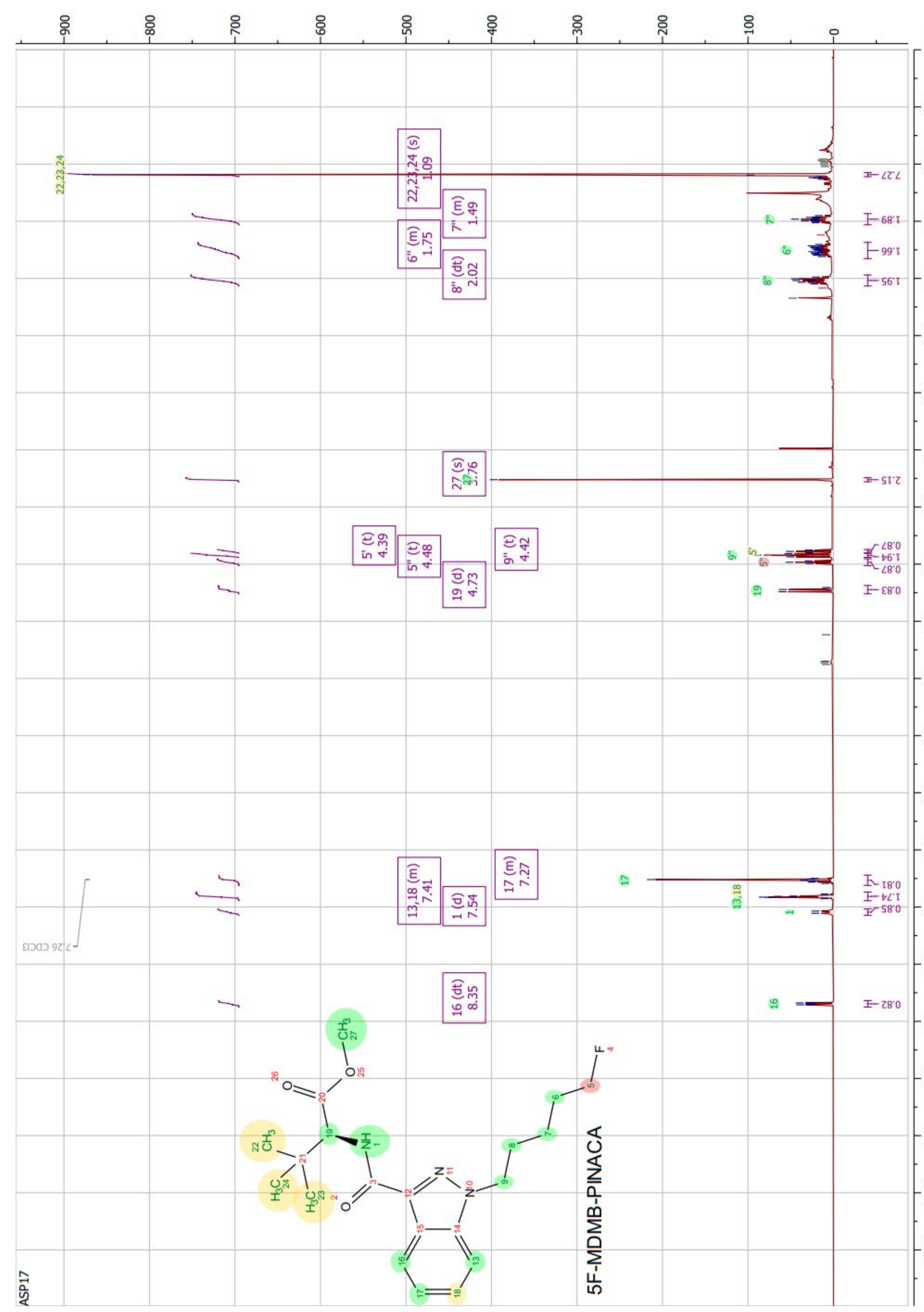

Figure S23. Labelled ^{1}H NMR of ASP17 in $CDCl_3$.

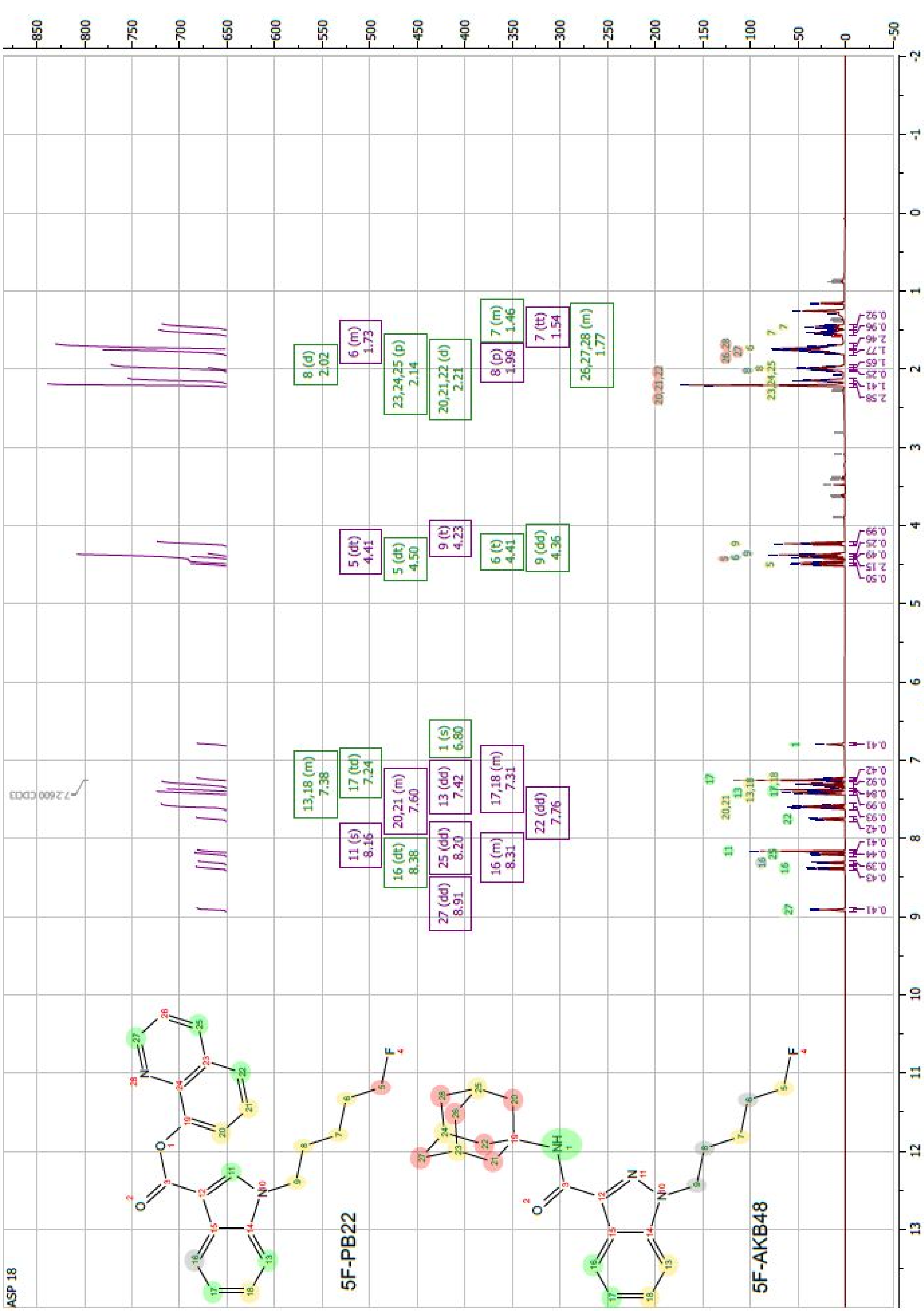

Figure S24. Labelled ^{1}H NMR of ASP18 in $CDCl_3$.

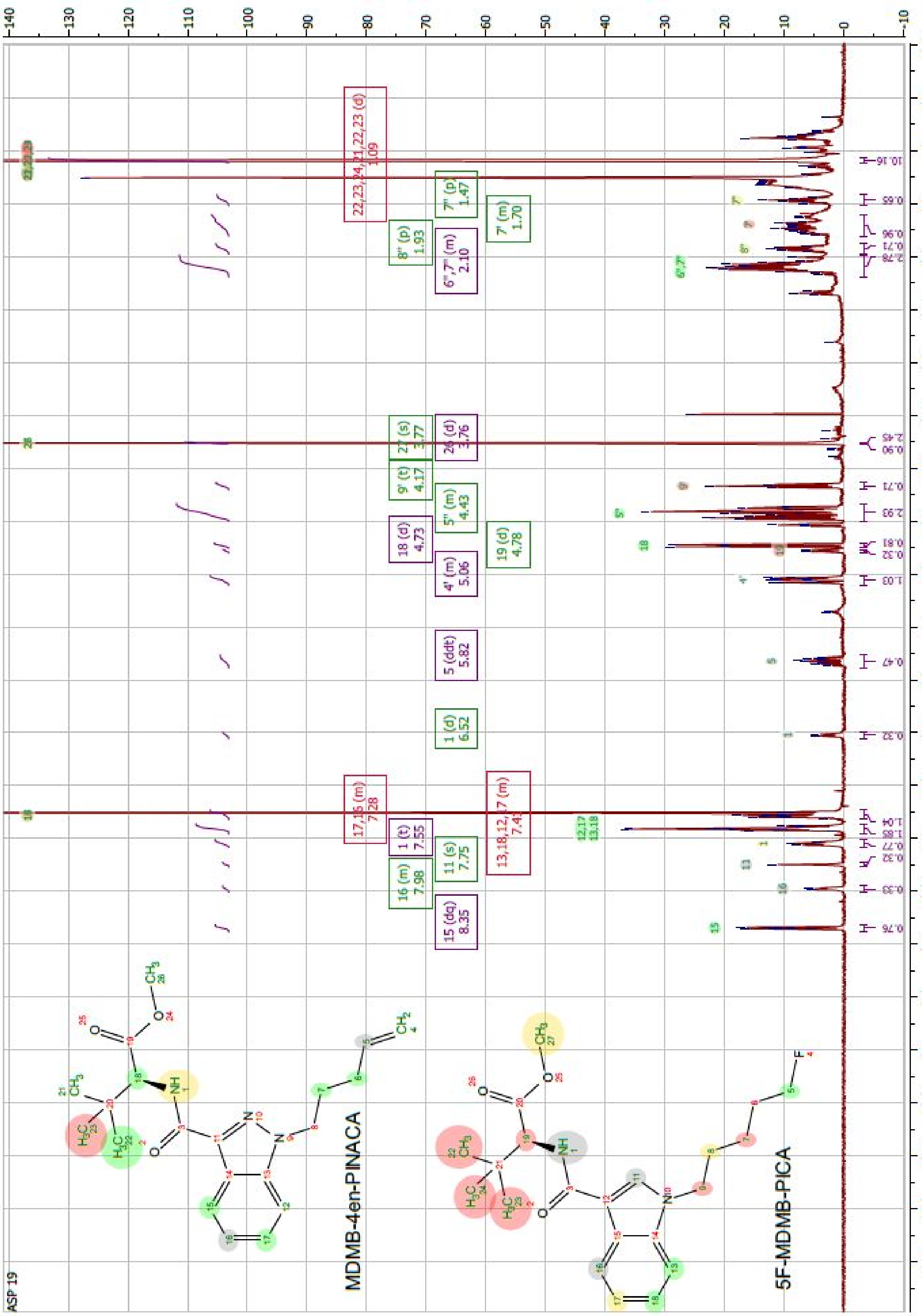

Figure S25. Labelled ^{1}H NMR of ASP19 in $CDCl_3$.

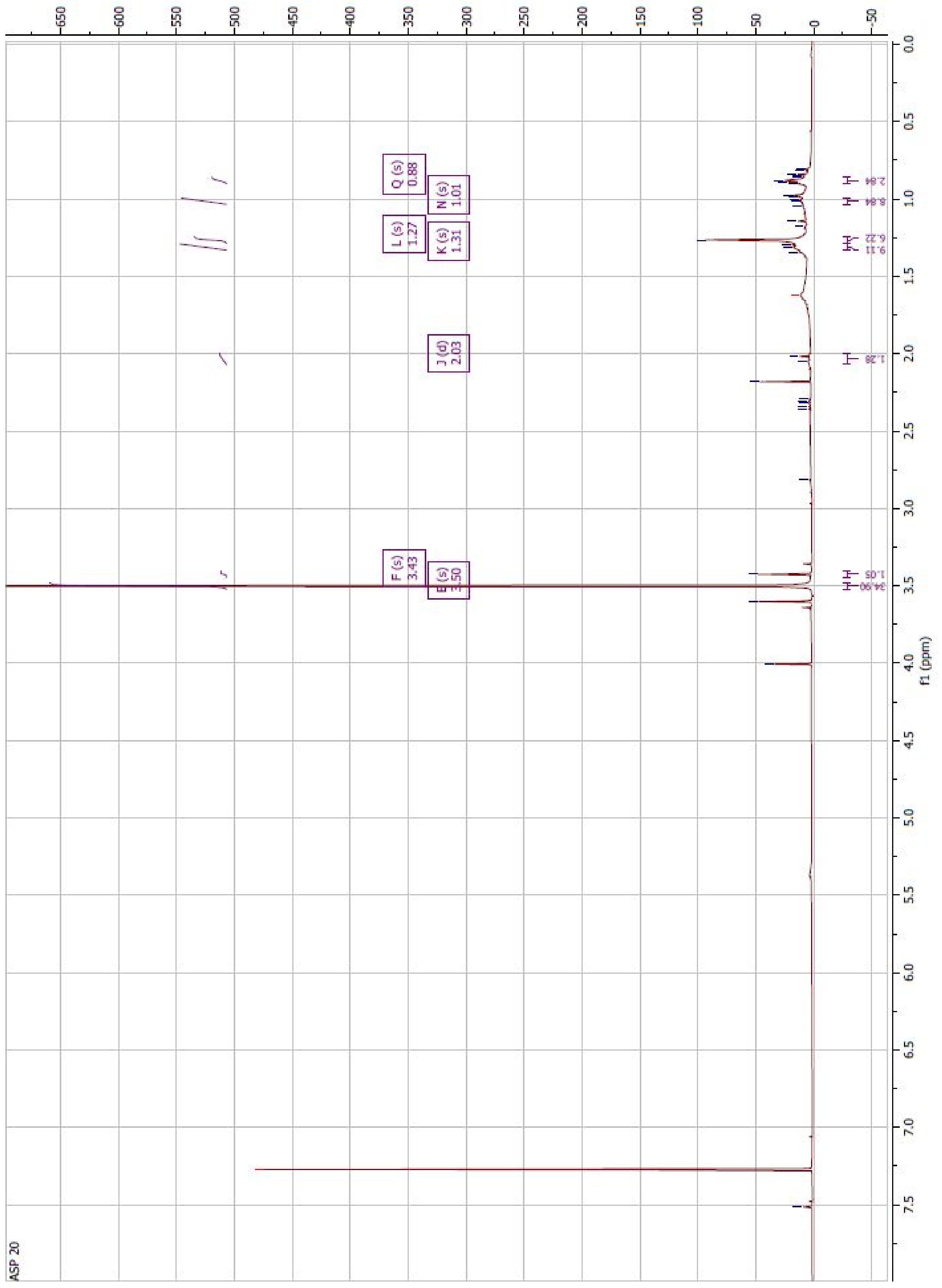

Figure S26. Labelled ^{1}H NMR of ASP20 in $CDCl_3$.

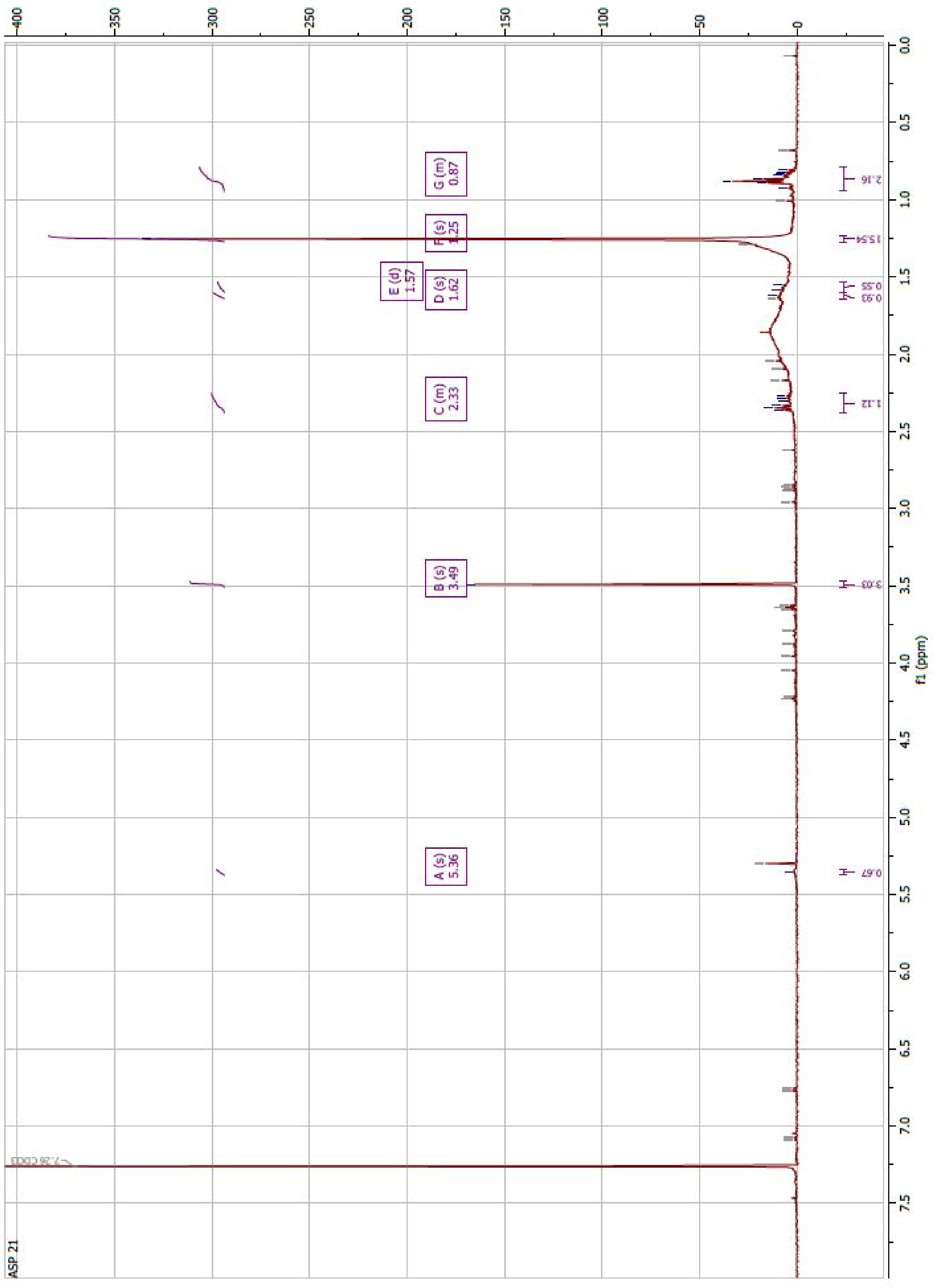

Figure S27. Labelled ^{1}H NMR of ASP21 in $CDCl_3$.

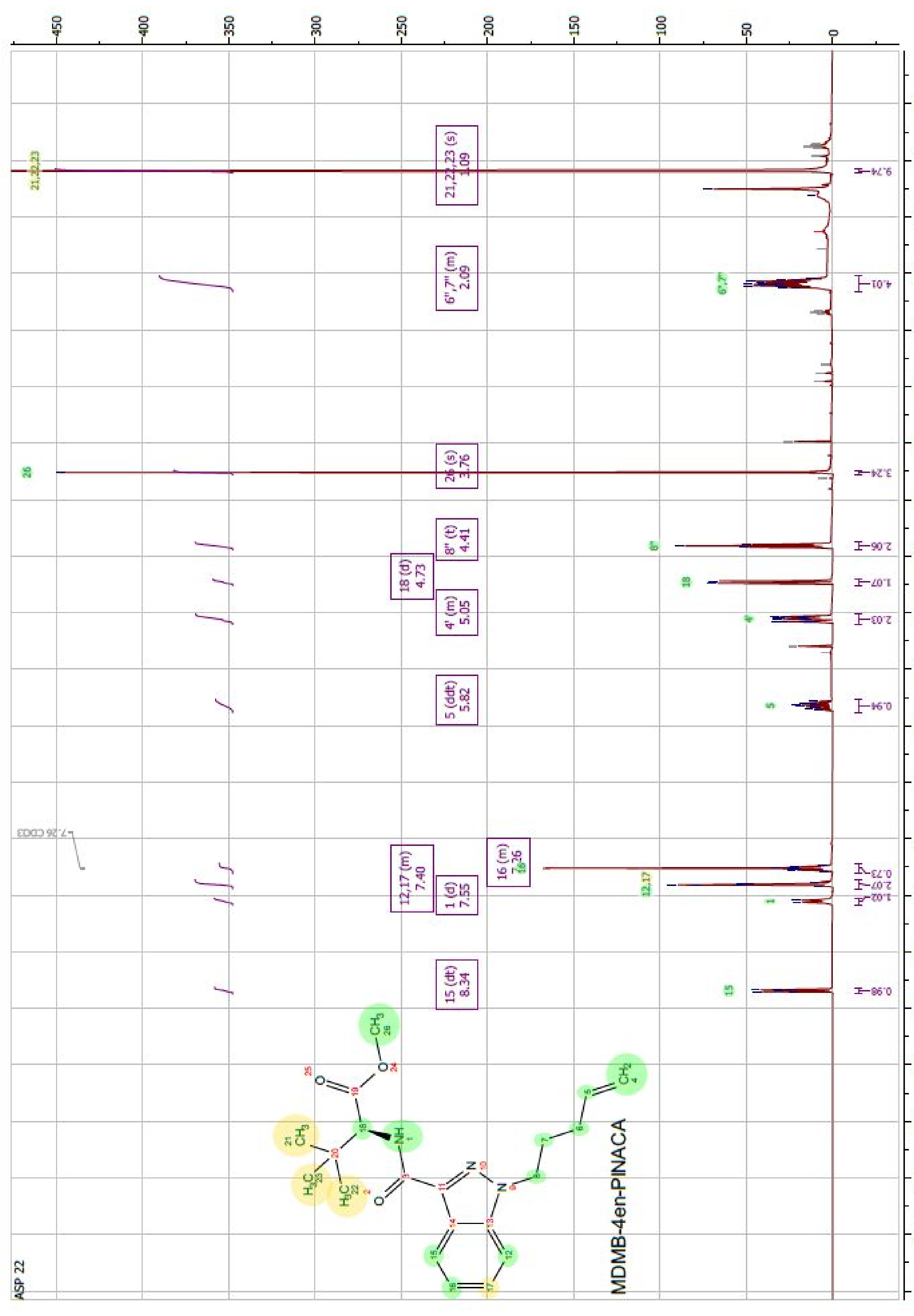

Figure S28. Labelled ^{1}H NMR of ASP22 in $CDCl_3$.

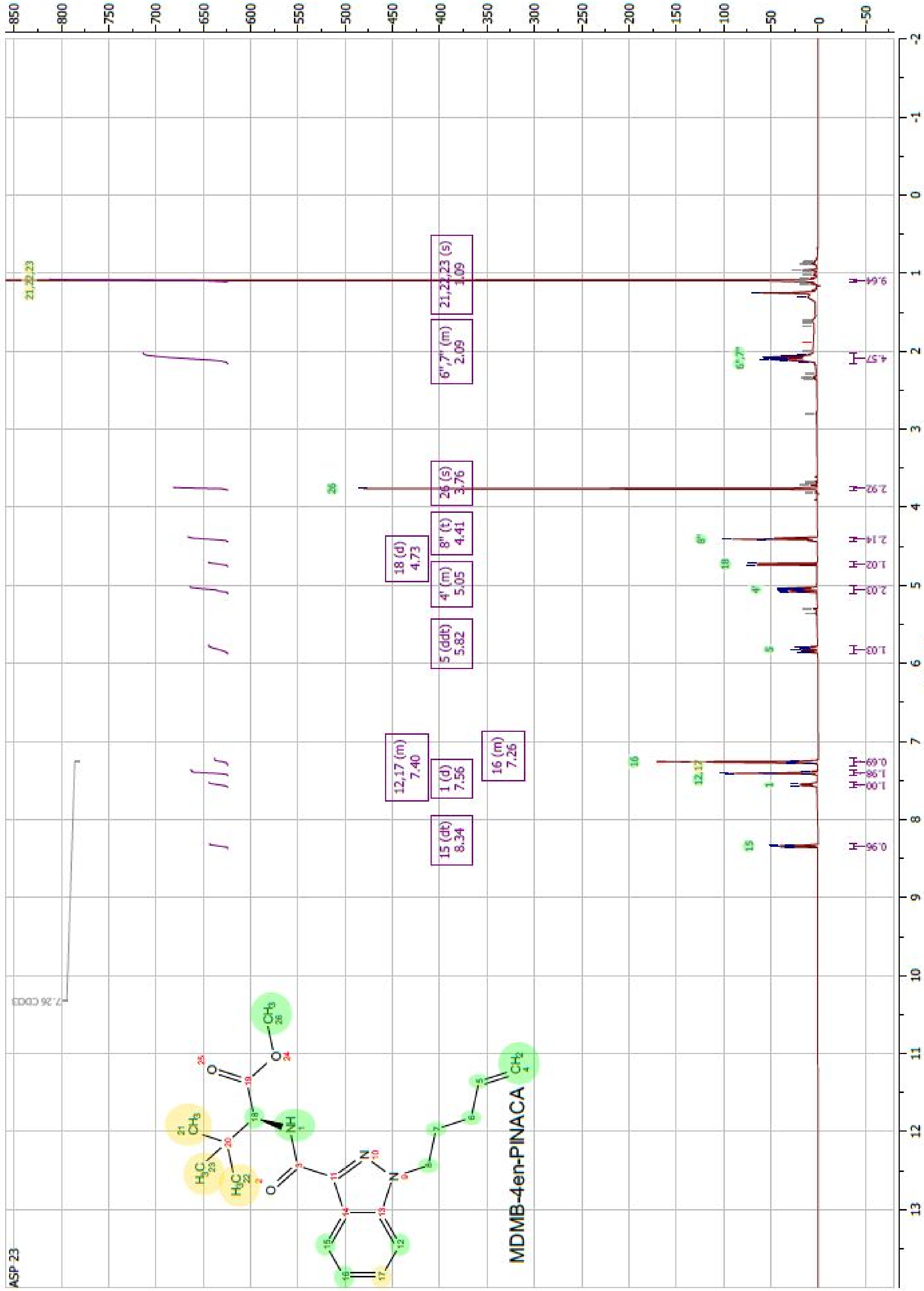

Figure S29. Labelled ^{1}H NMR of ASP23 in $CDCl_3$.

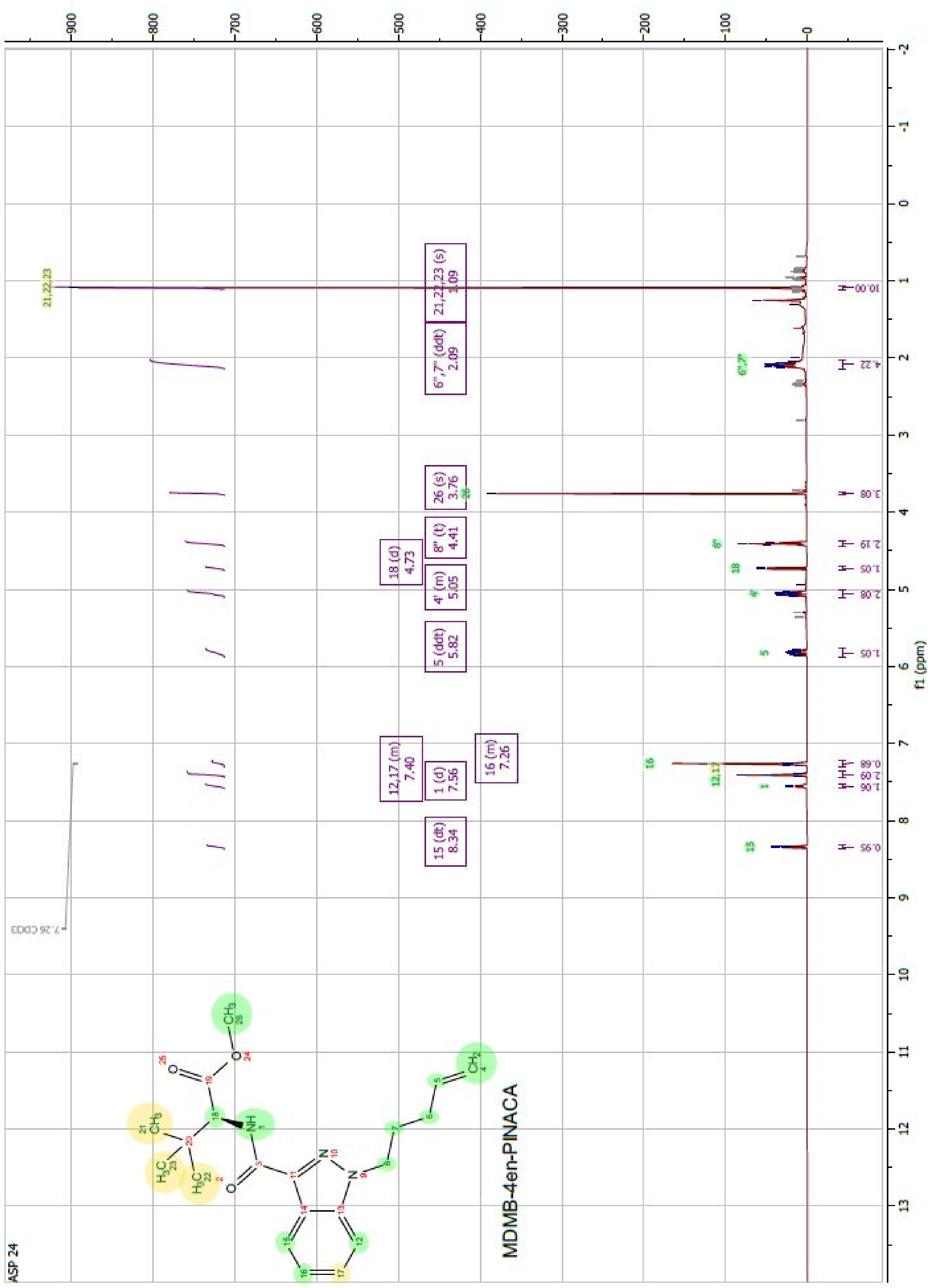

Figure S30. Labelled ^{1}H NMR of ASP24 in $CDCl_3$.

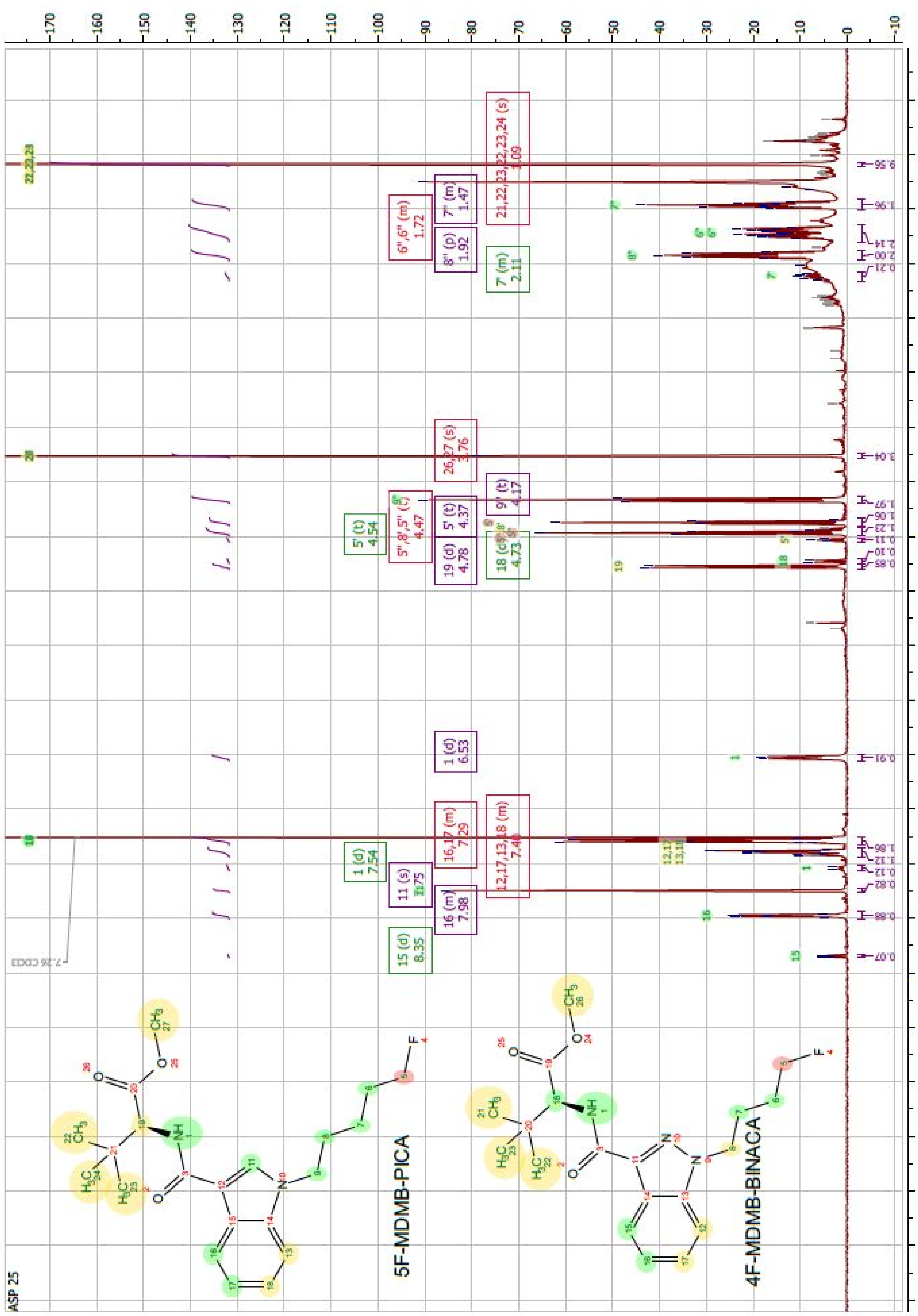

Figure S31. Labelled ^{1}H NMR of ASP25 in $CDCl_3$.

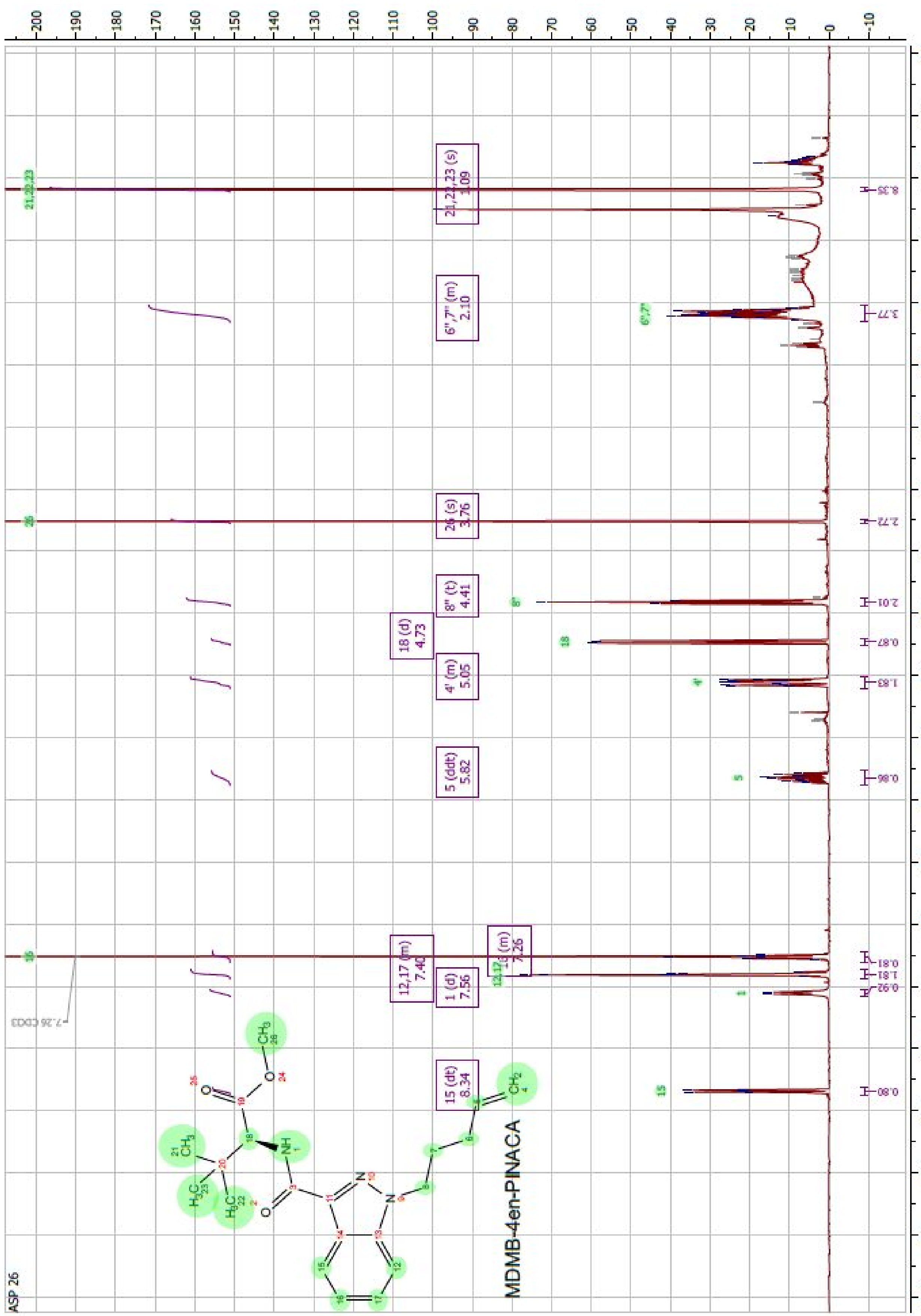

Figure S32. Labelled ^{1}H NMR of ASP26 in $CDCl_3$.

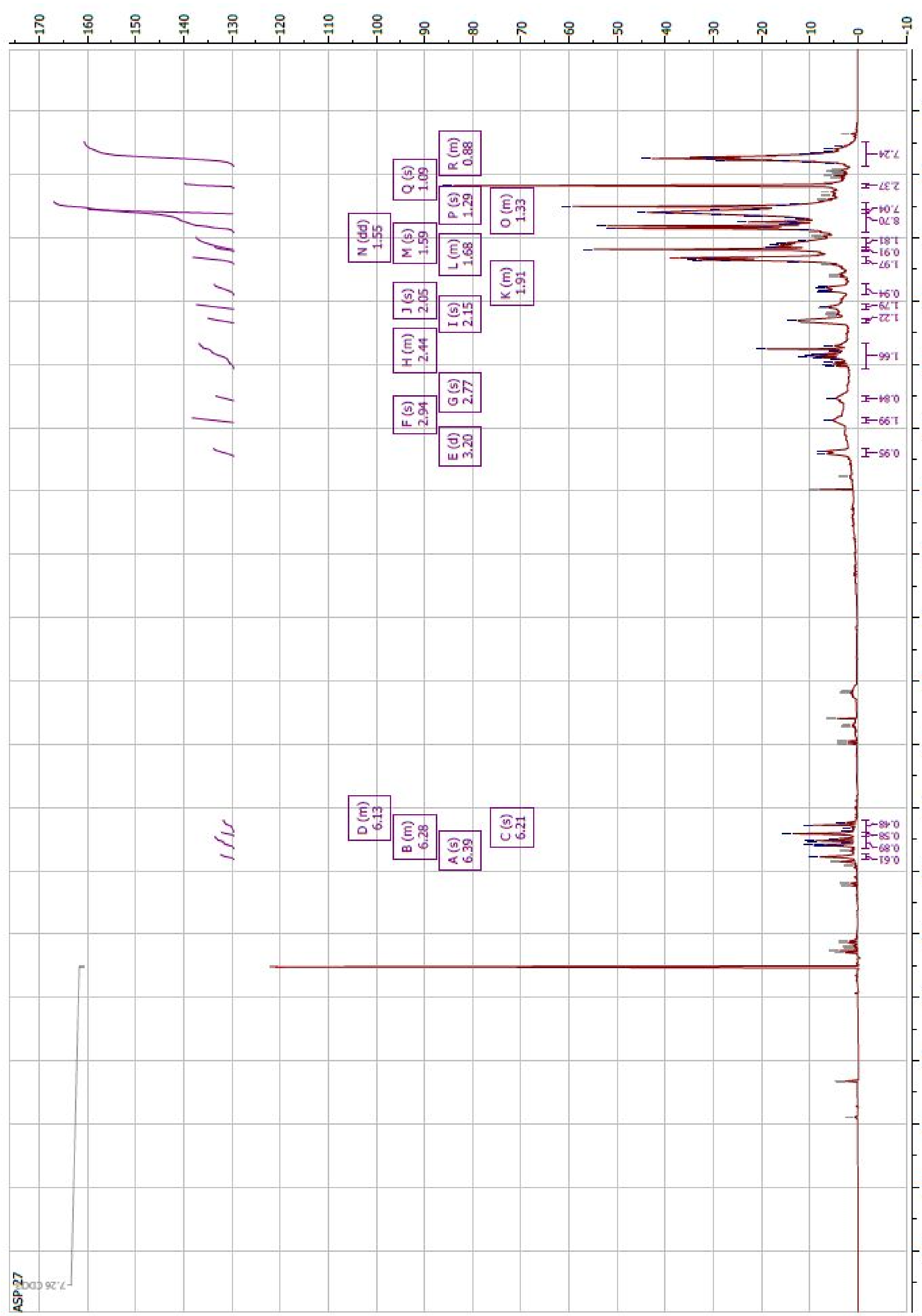

Figure S33. Labelled ^{1}H NMR of ASP27 in $CDCl_3$.

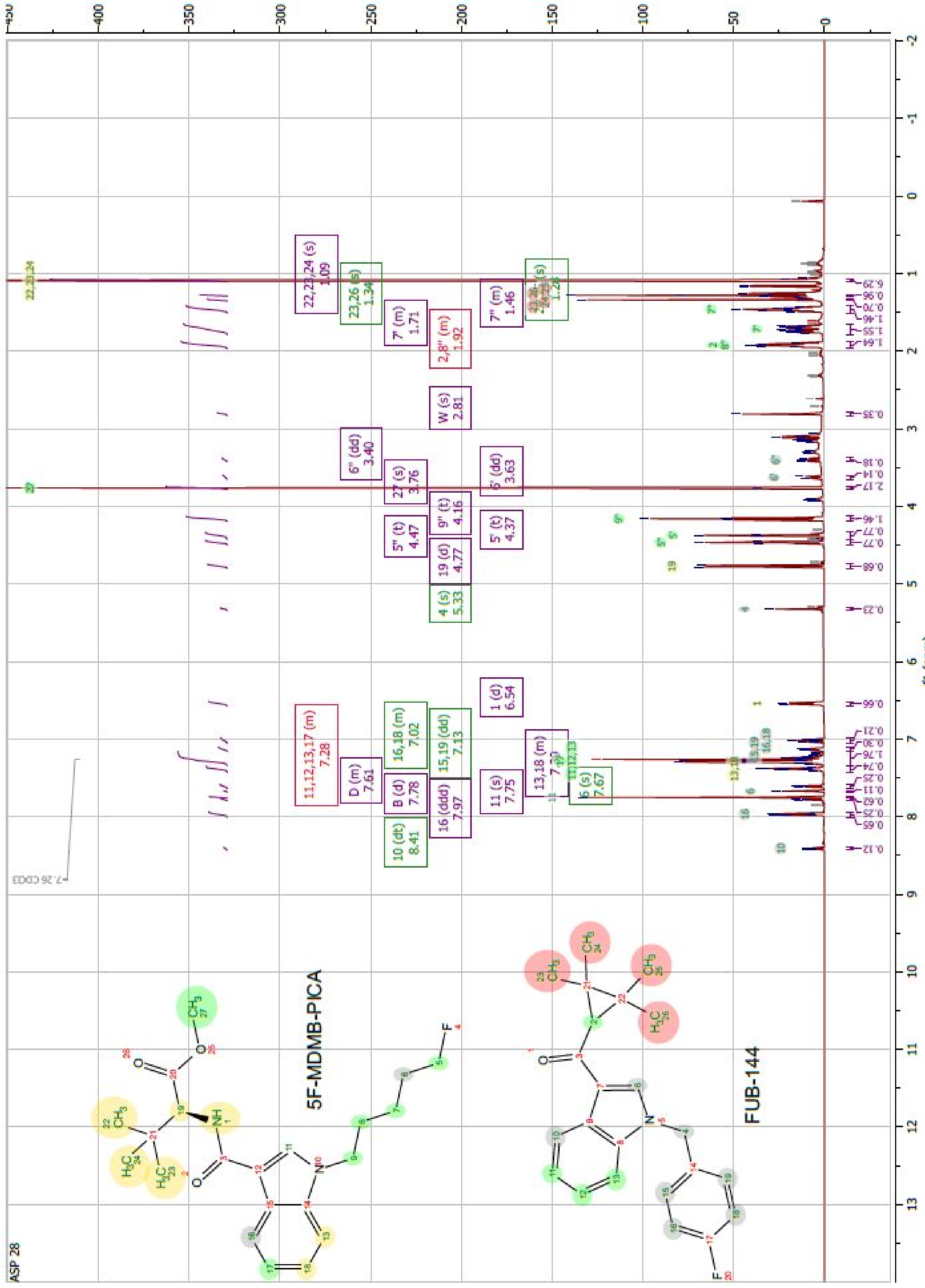

Figure S34. Labelled ^{1}H NMR of ASP28 in $CDCl_3$.

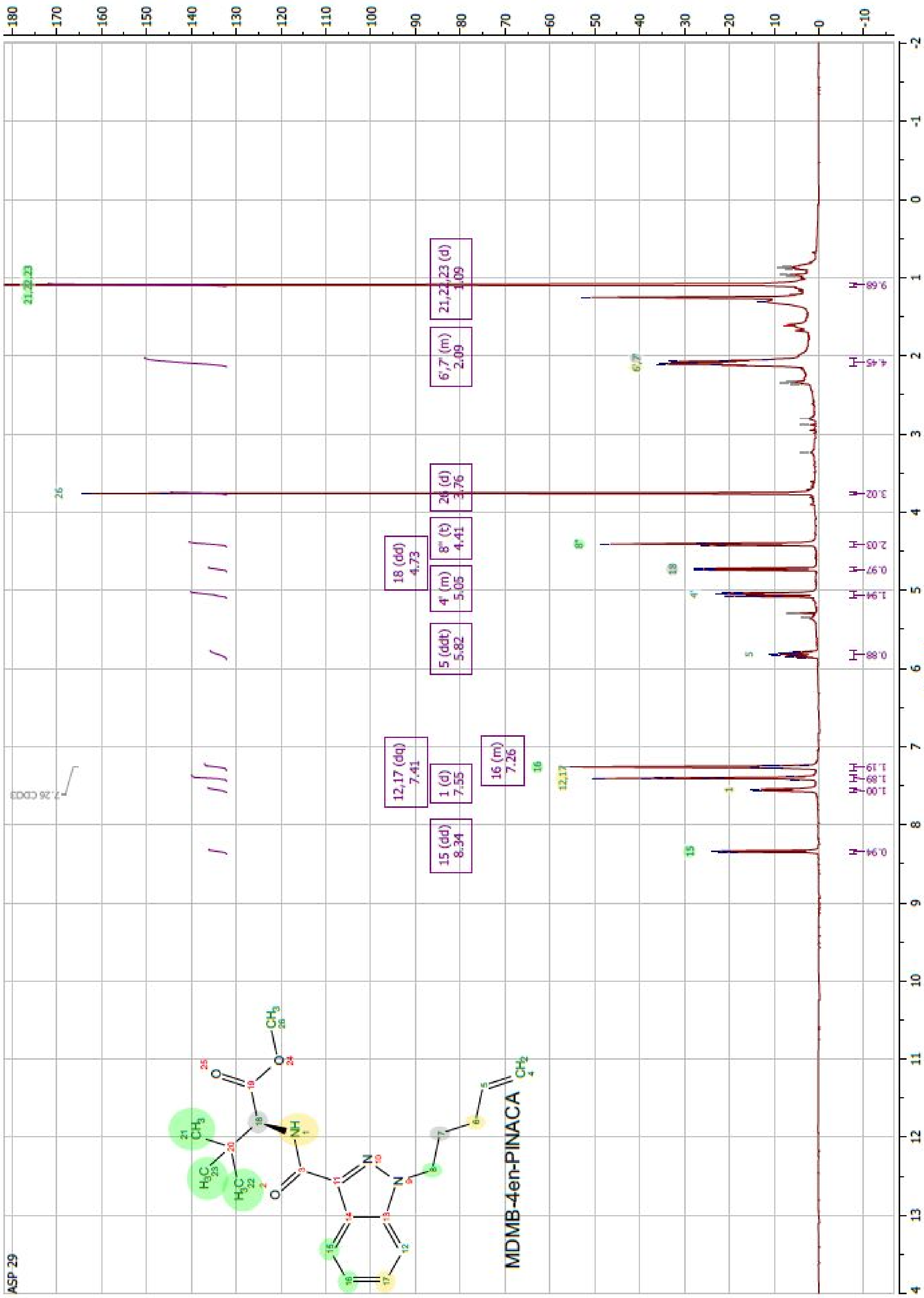

Figure S35. Labelled ^{1}H NMR of ASP29 in $CDCl_3$.

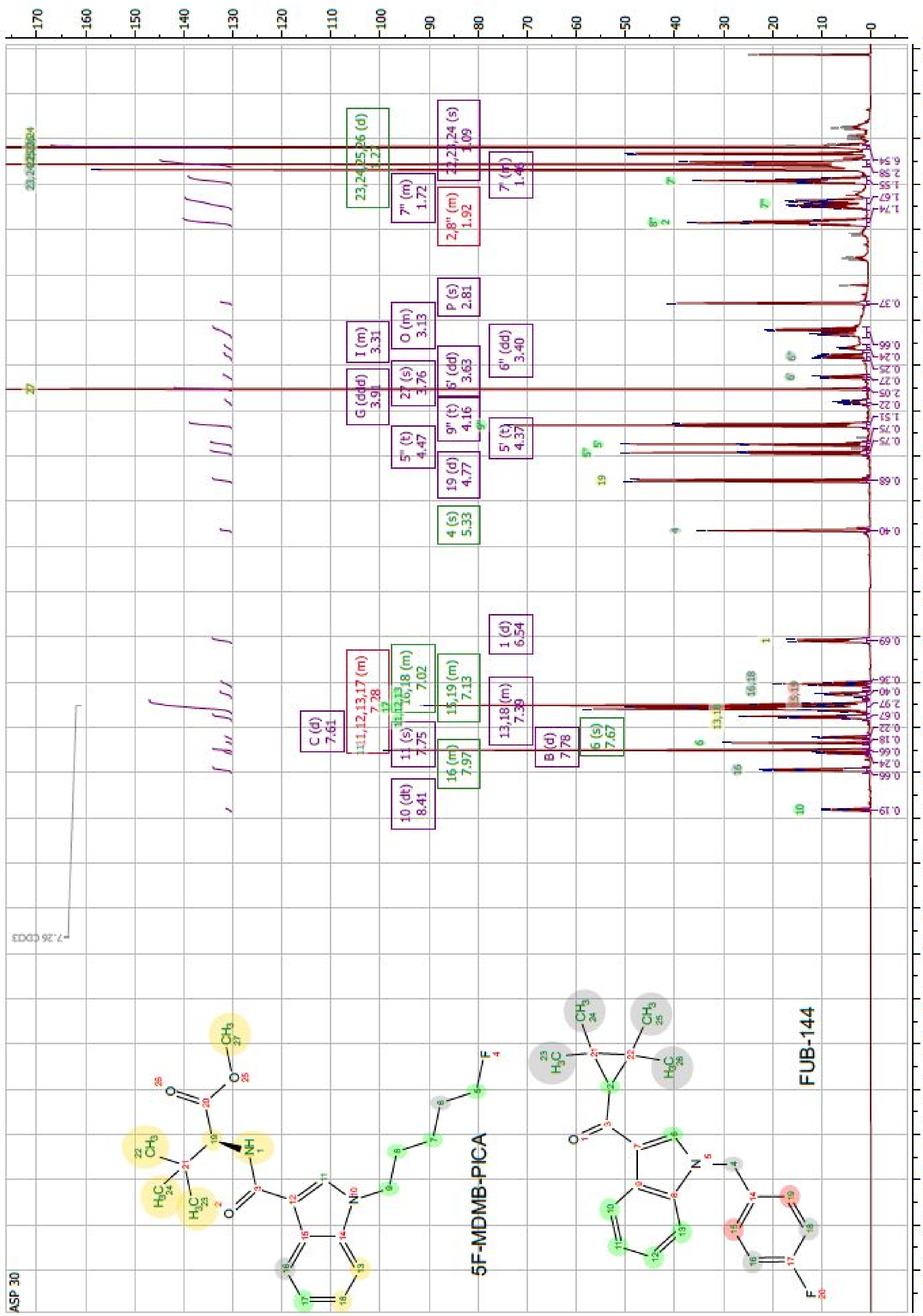

Figure S36. Labelled ^{1}H NMR of ASP30 in $CDCl_3$.

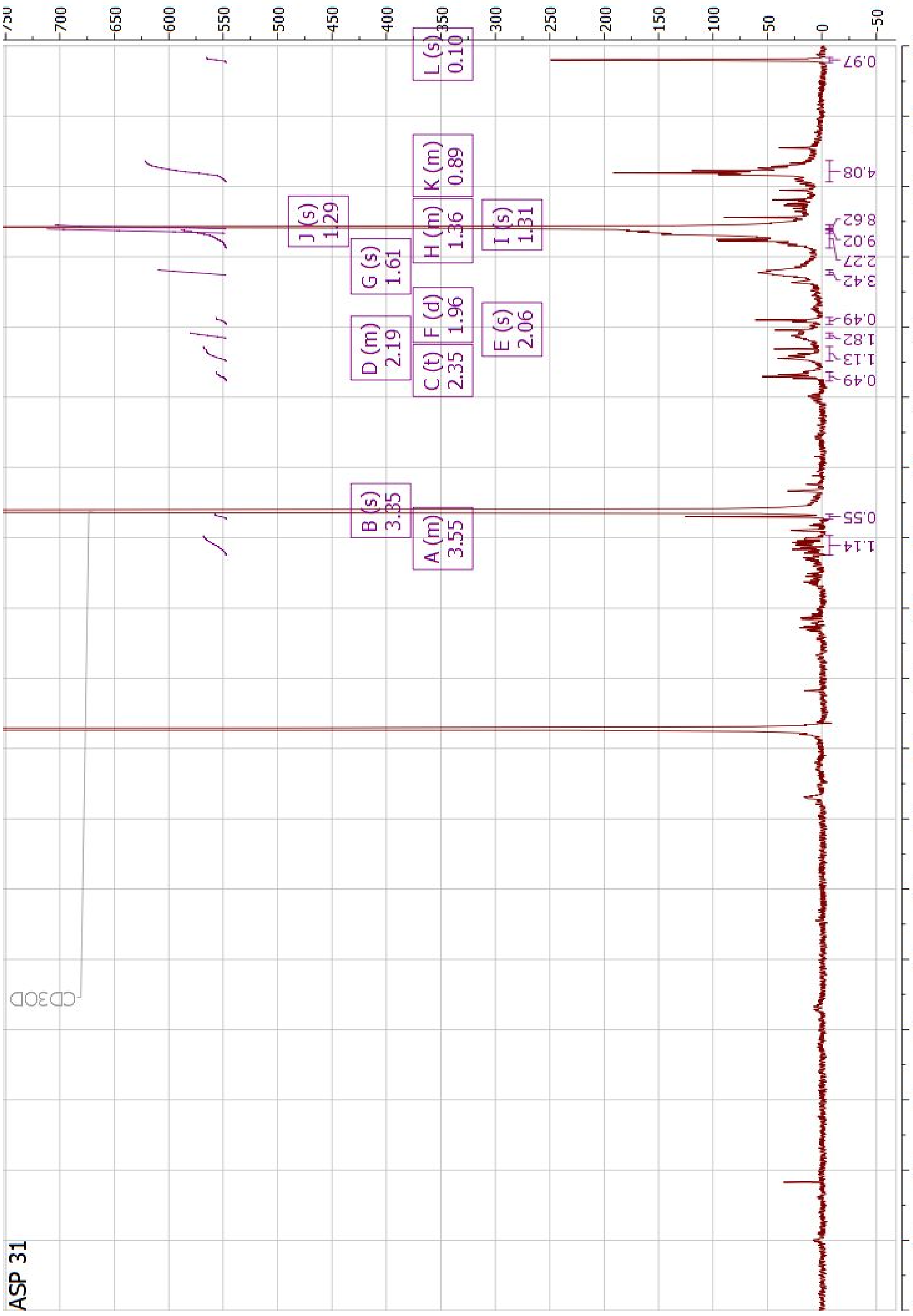

Figure S37. Labelled ^{1}H NMR of ASP31 in CD_3OD.

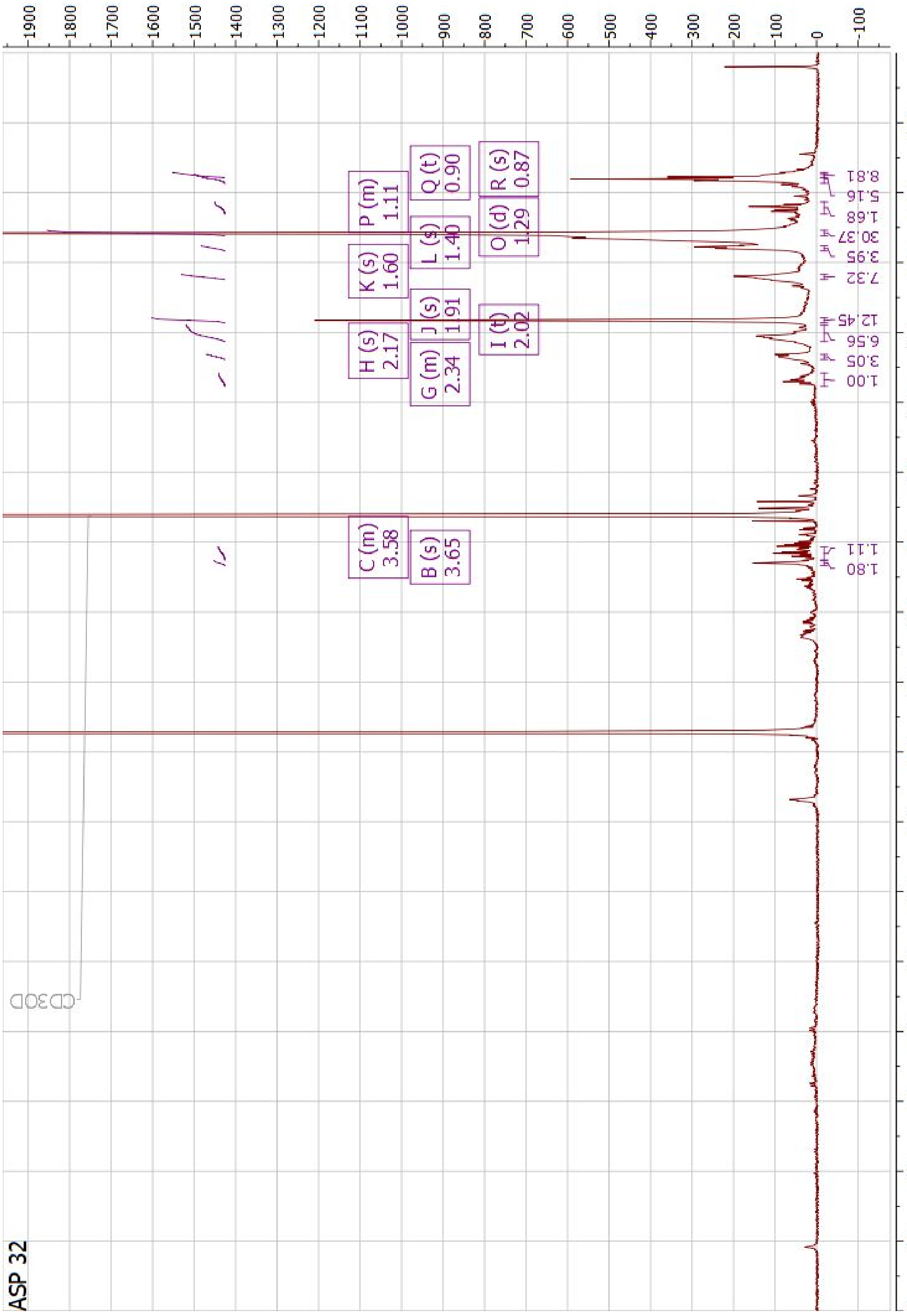

Figure S38. Labelled ^{1}H NMR of ASP32 in CD_3OD.

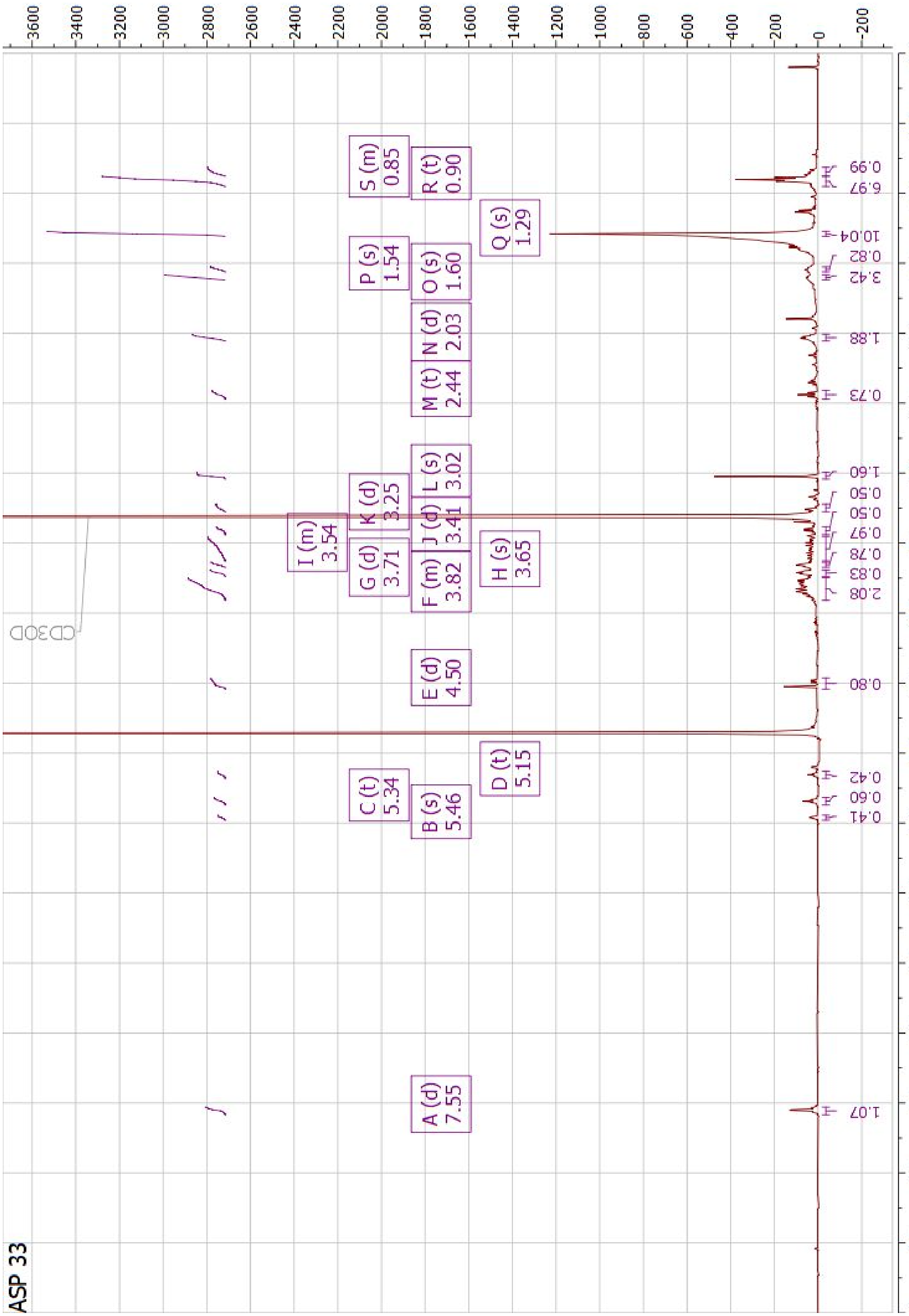

Figure S39. Labelled ^{1}H NMR of ASP33 in CD_3OD.

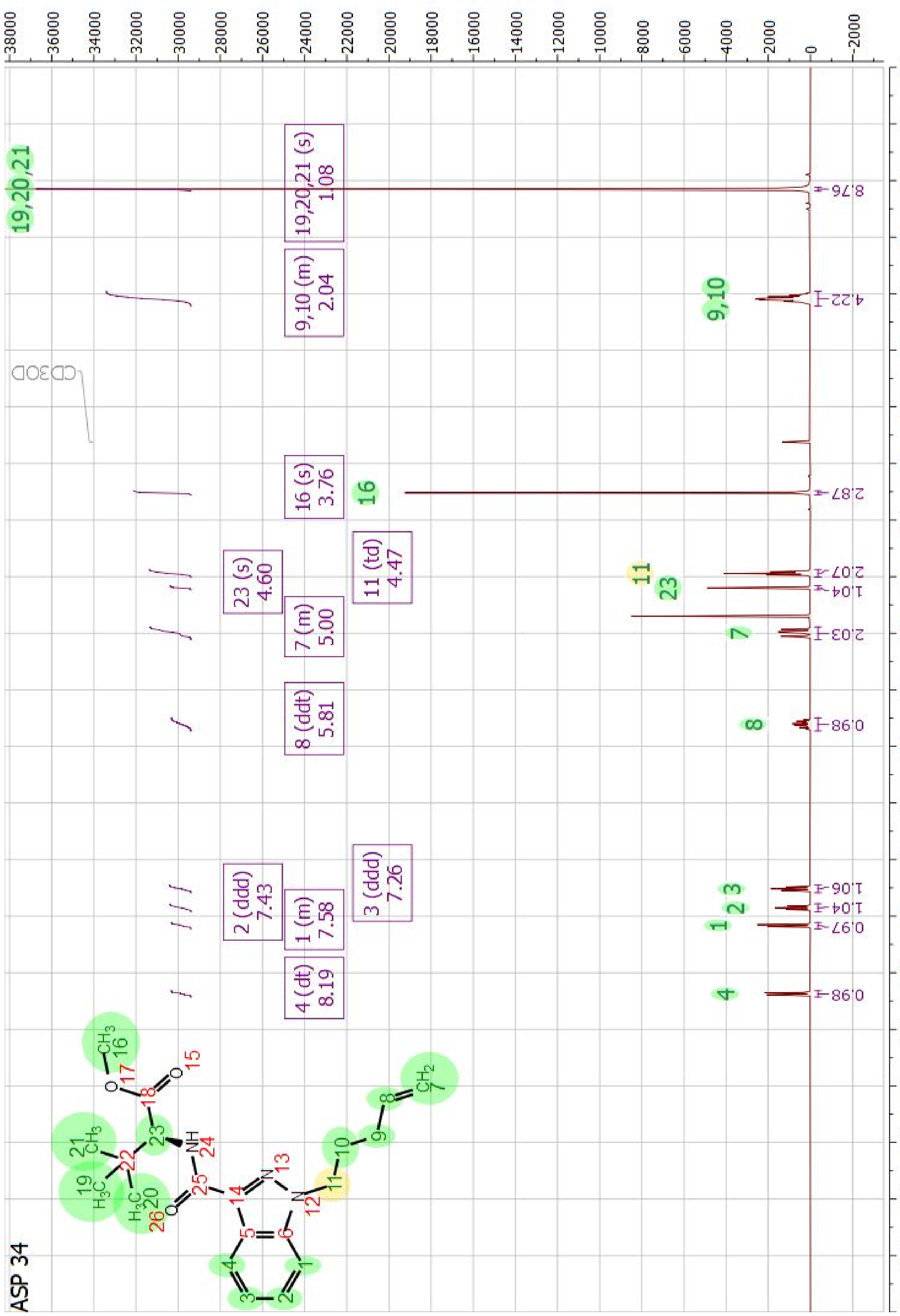

Figure S40. Labelled ^{1}H NMR of ASP34 in CD_3OD.

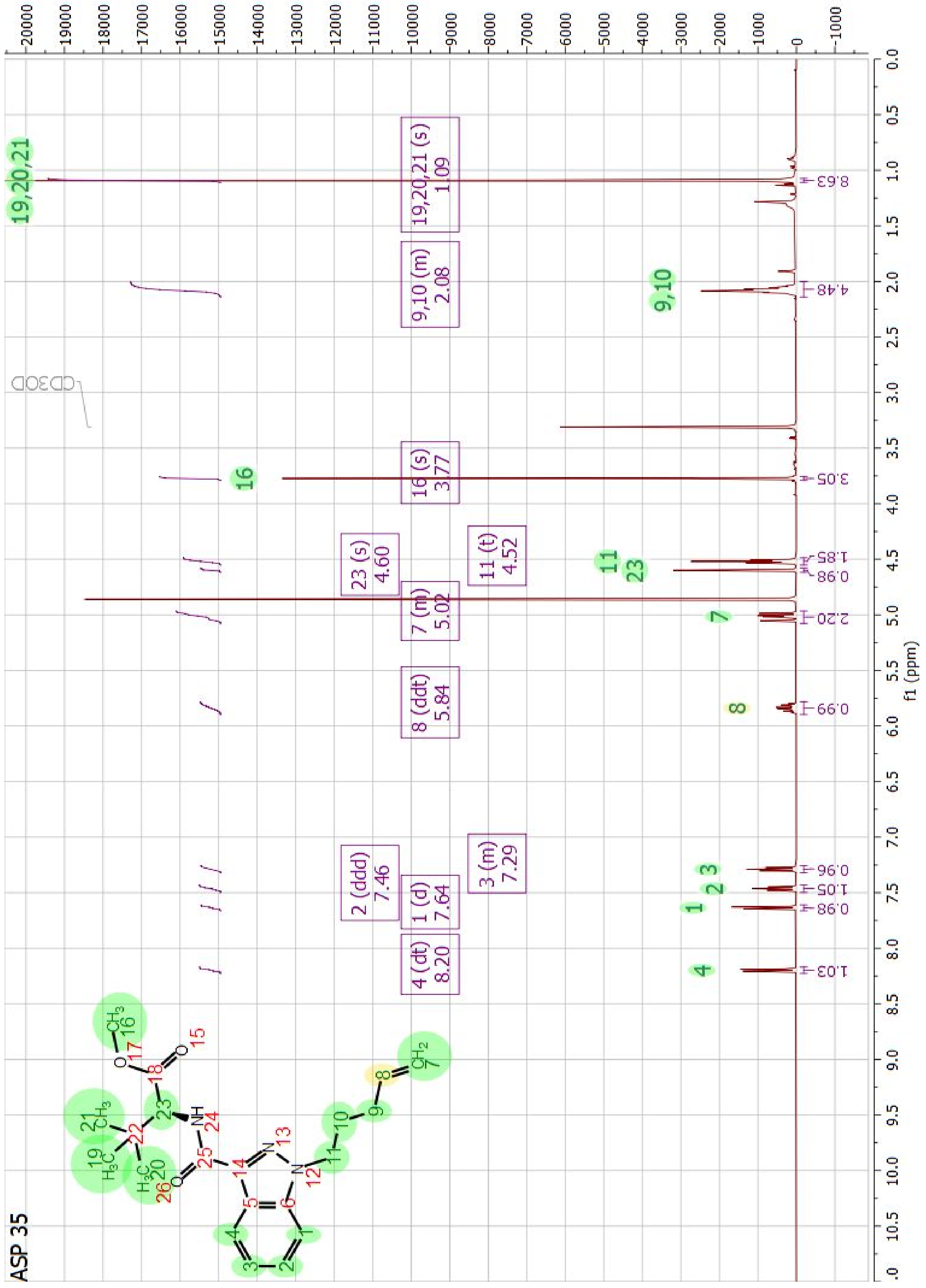

Figure S41. Labelled ^{1}H NMR of ASP35 in CD_3OD.

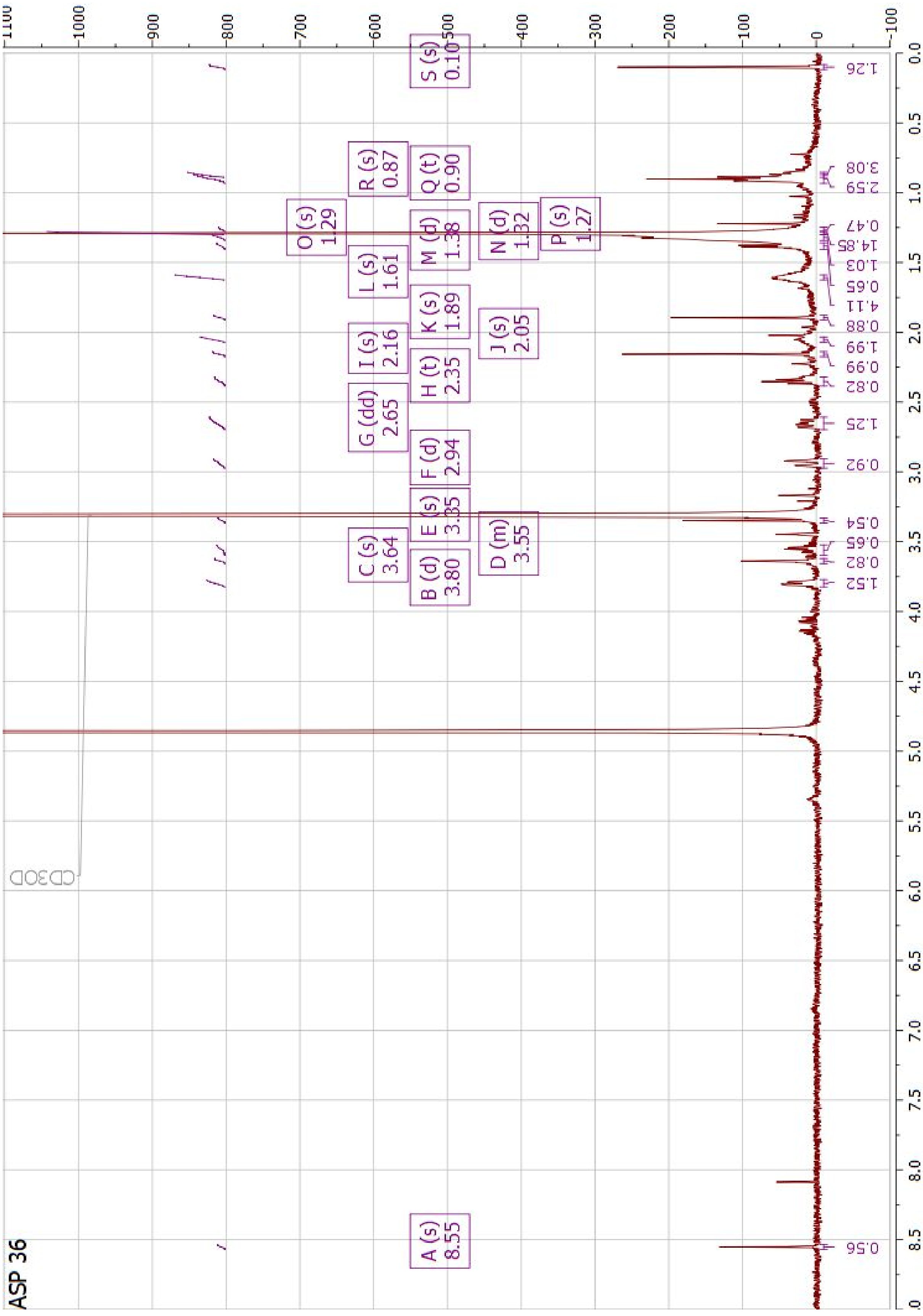

Figure S42. Labelled ^{1}H NMR of ASP36 in CD_3OD.

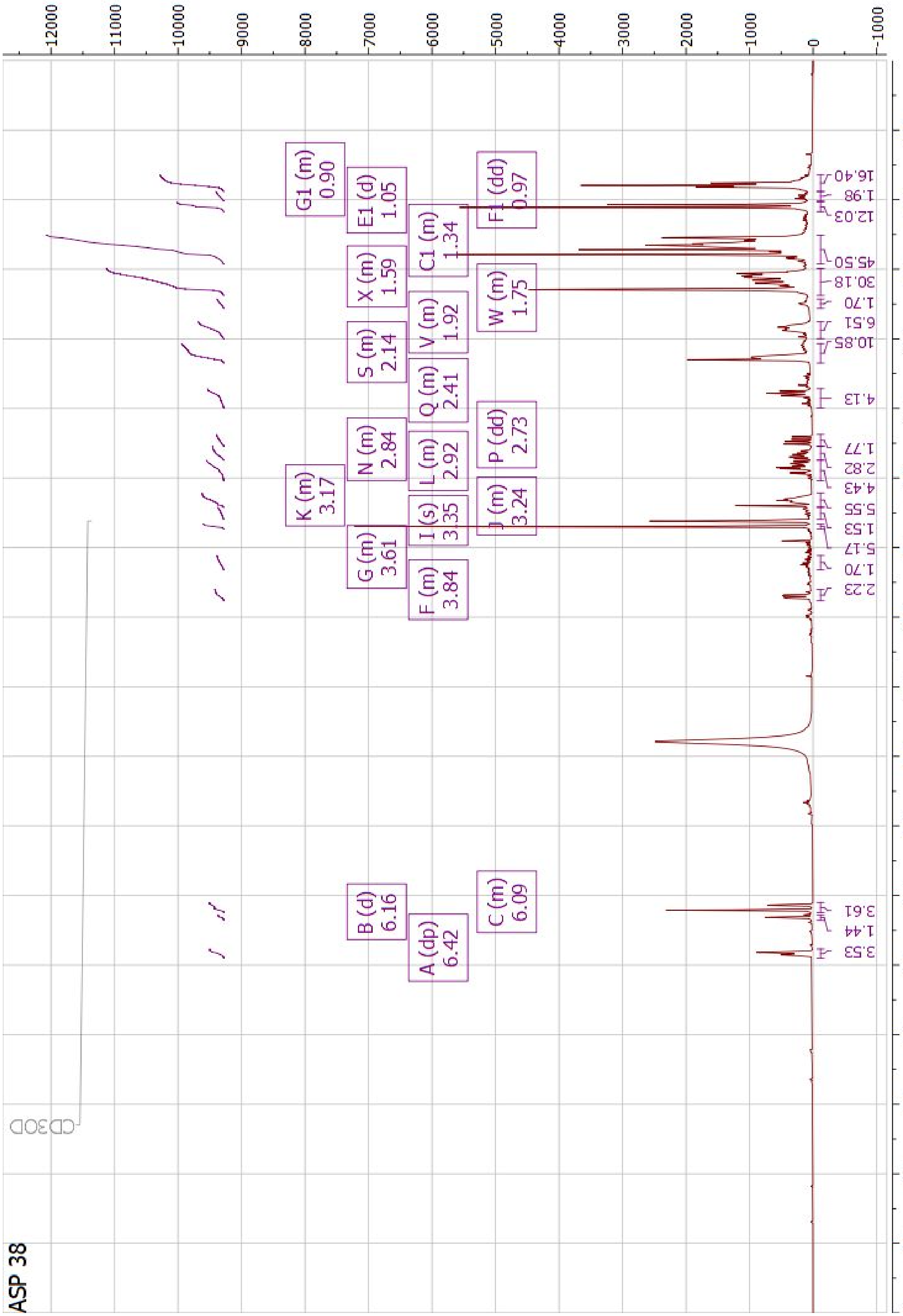

Figure S43. Labelled ^{1}H NMR of ASP38 in CD_3OD.

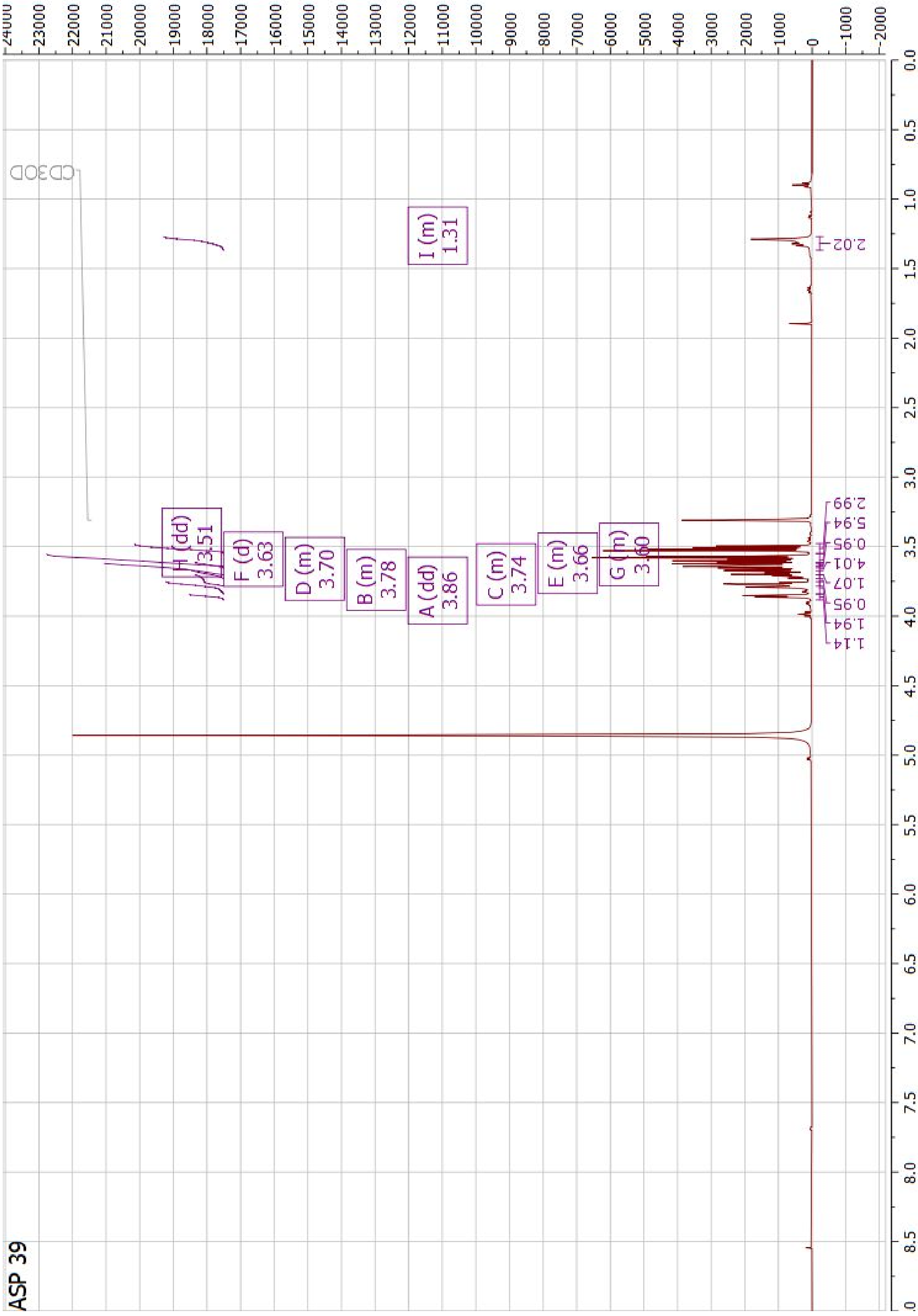

Figure S44. Labelled ^{1}H NMR of ASP39 in CD_3OD.

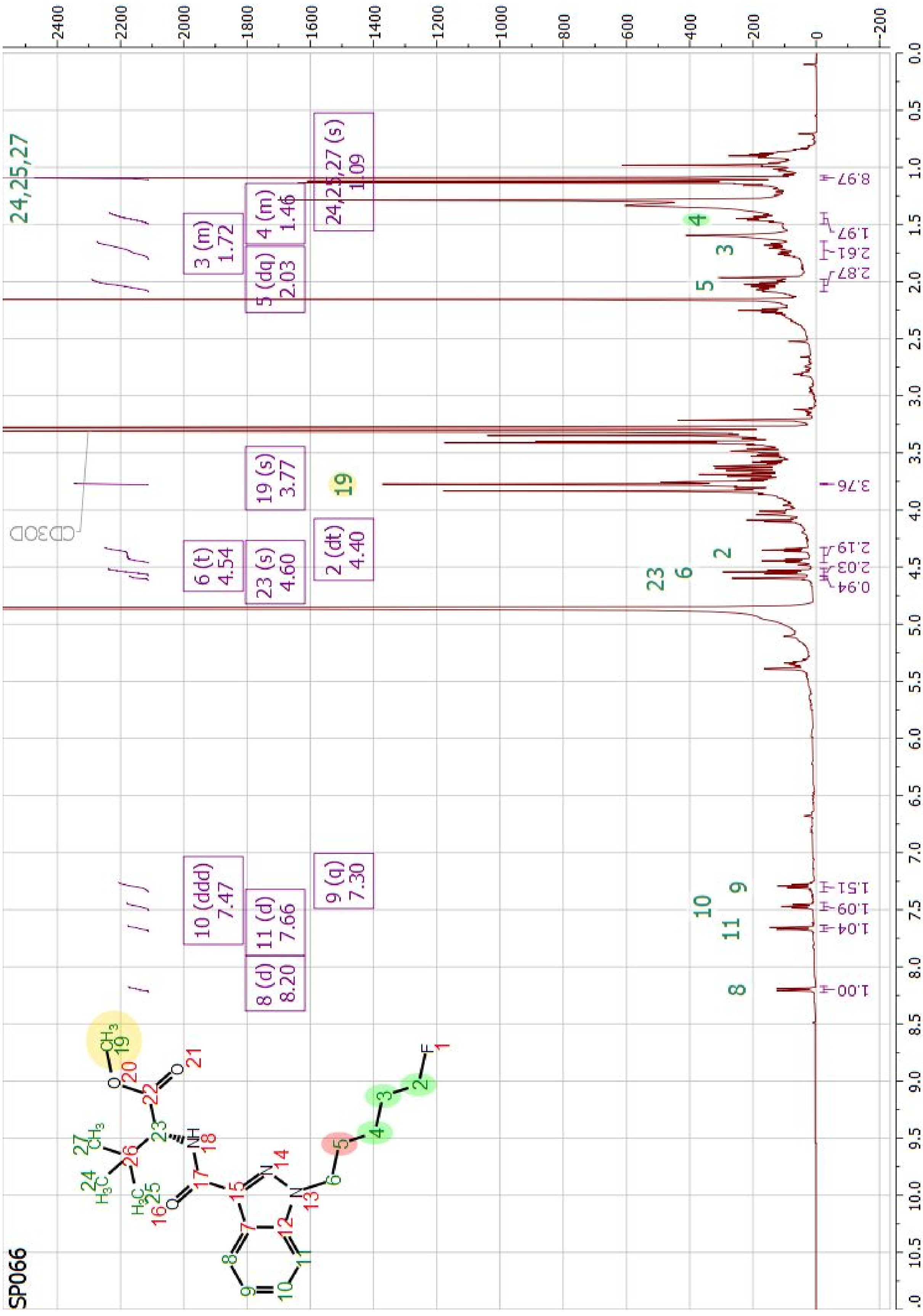

Figure S45. Labelled ^{1}H NMR of SP066 in CD_3OD.

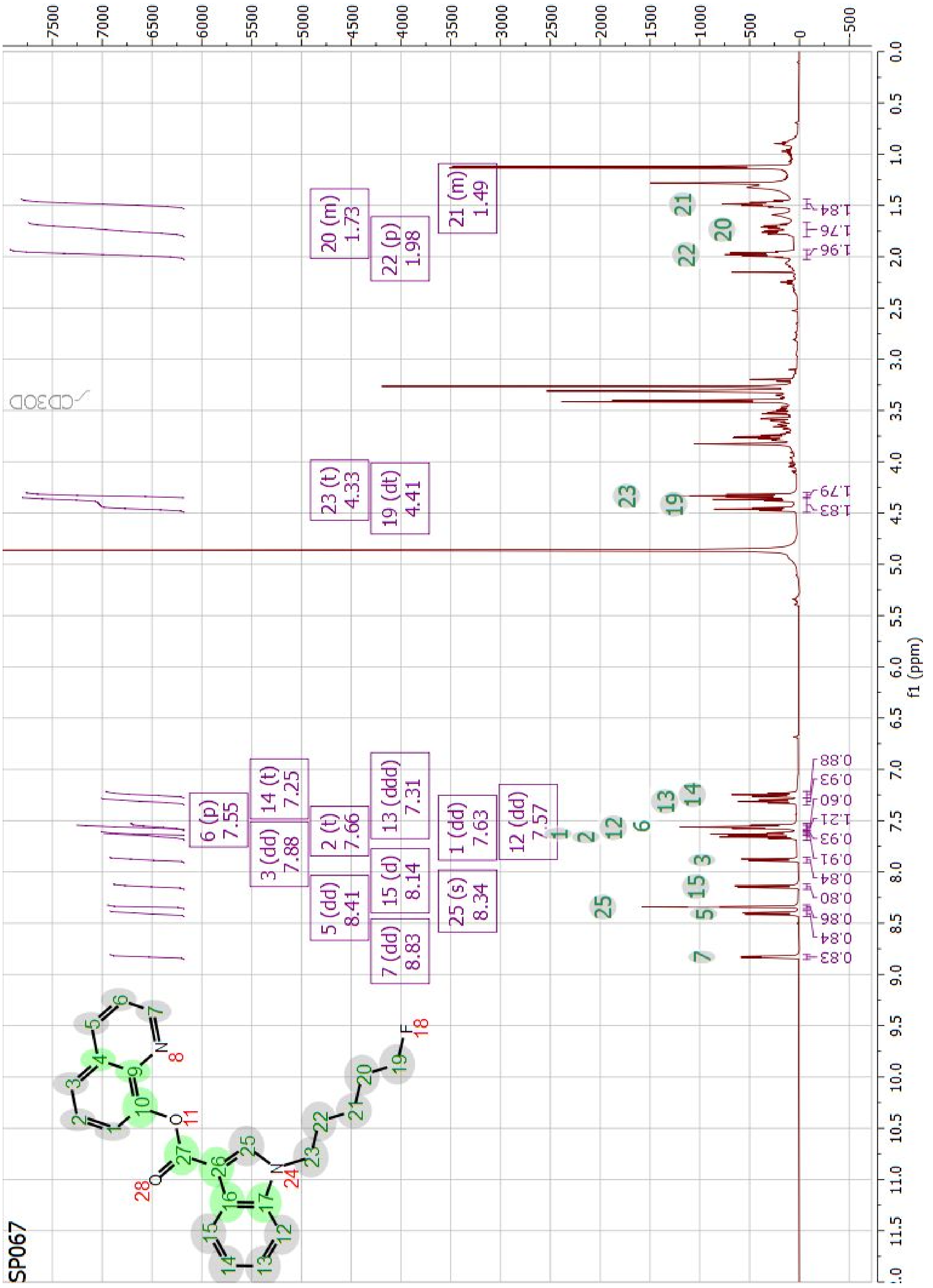

Figure S46. Labelled ^{1}H NMR of SP067 in CD_3OD.

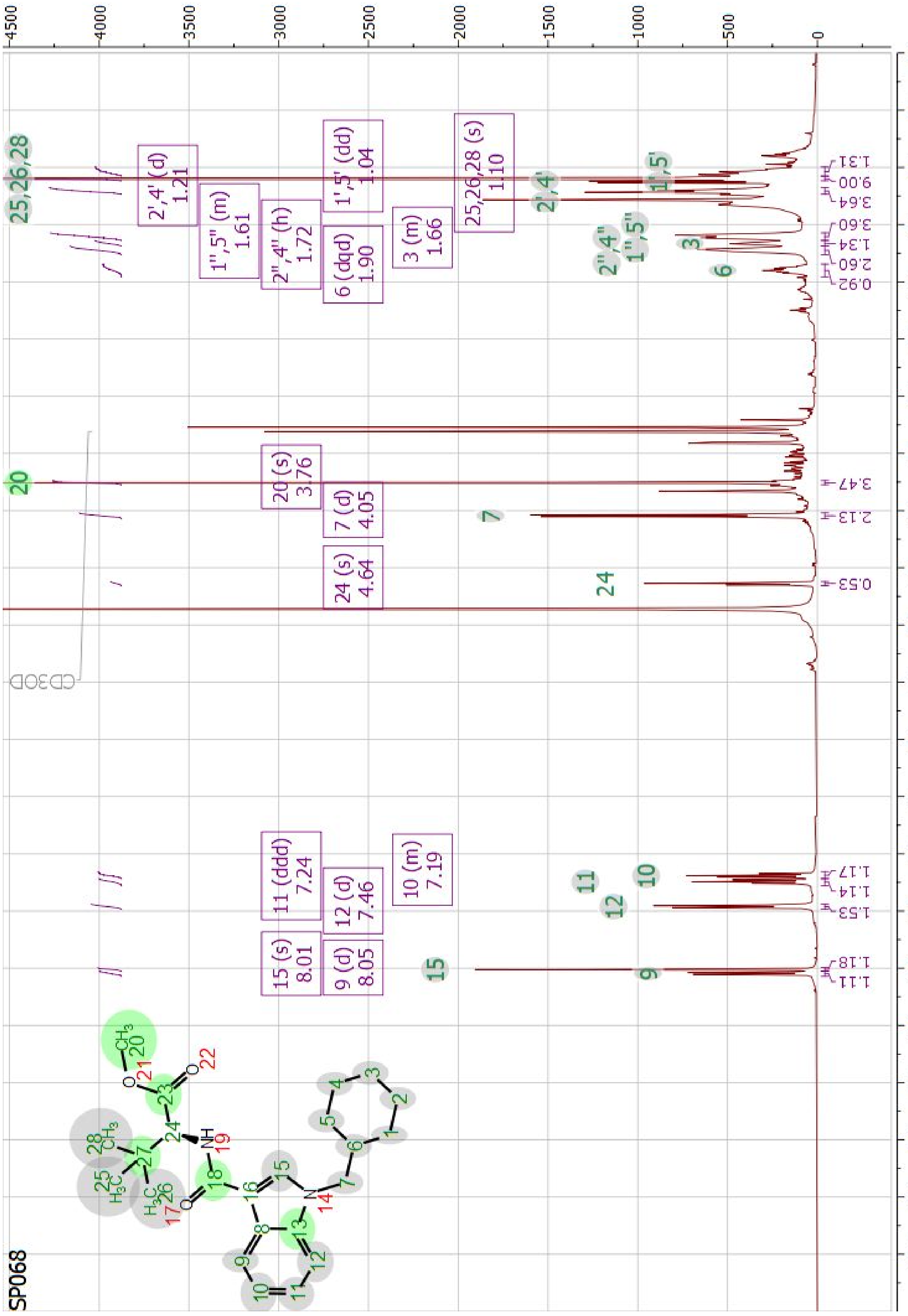

Figure S47. Labelled ^{1}H NMR of SP068 in CD_3OD.

ASP2: 5F-MDMB-PINACA

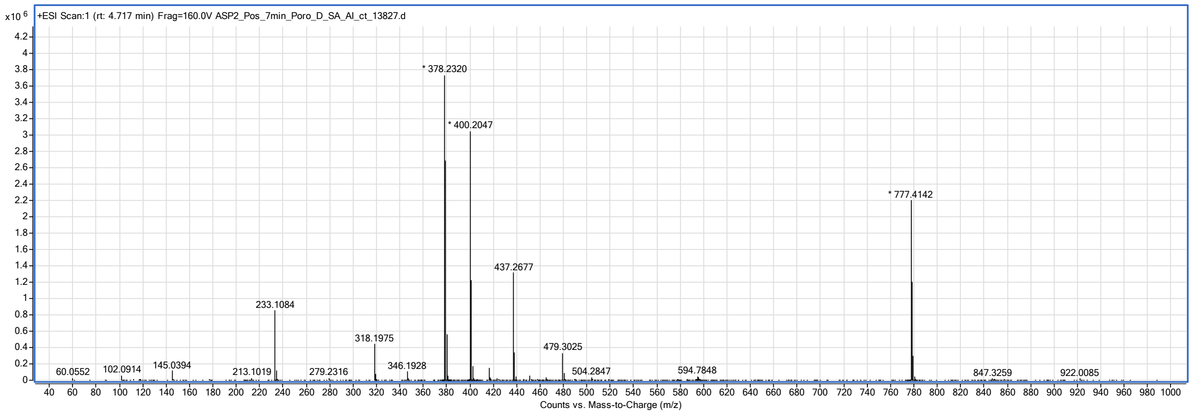

Figure S48. Extracted Mass Spectra from LC-MS Analysis for ASP2.

ASP3: AMB-FUBINACA

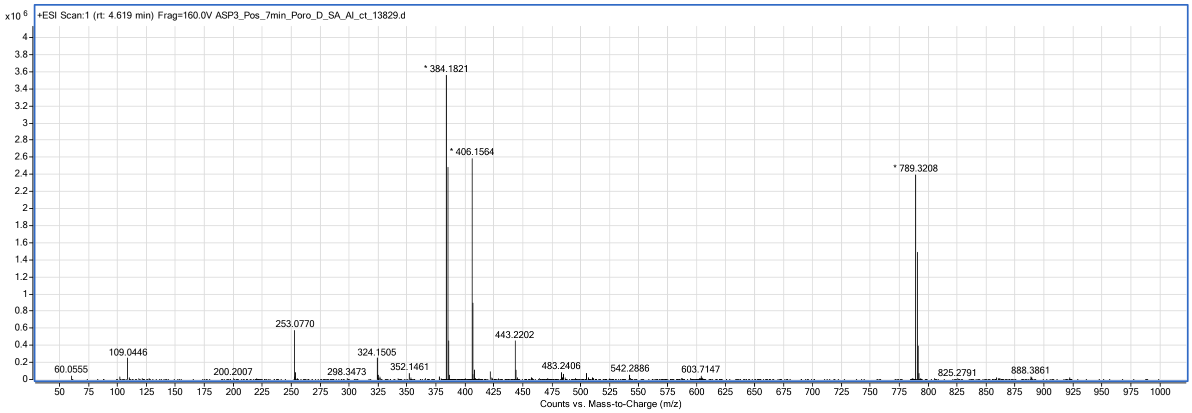

5F-MDMB-PINACA

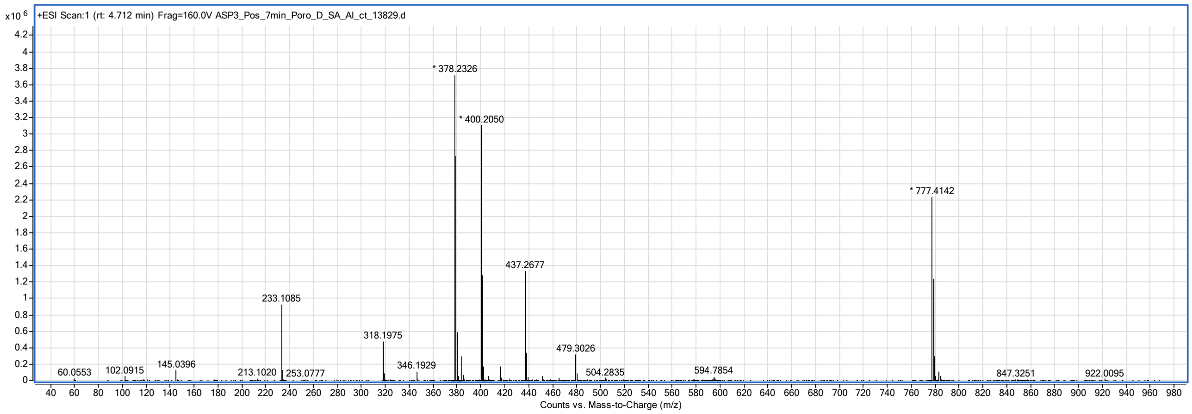

Figure S49. Extracted Mass Spectra from LC-MS Analysis for ASP3.

ASP4: MDMB-4en-PINACA

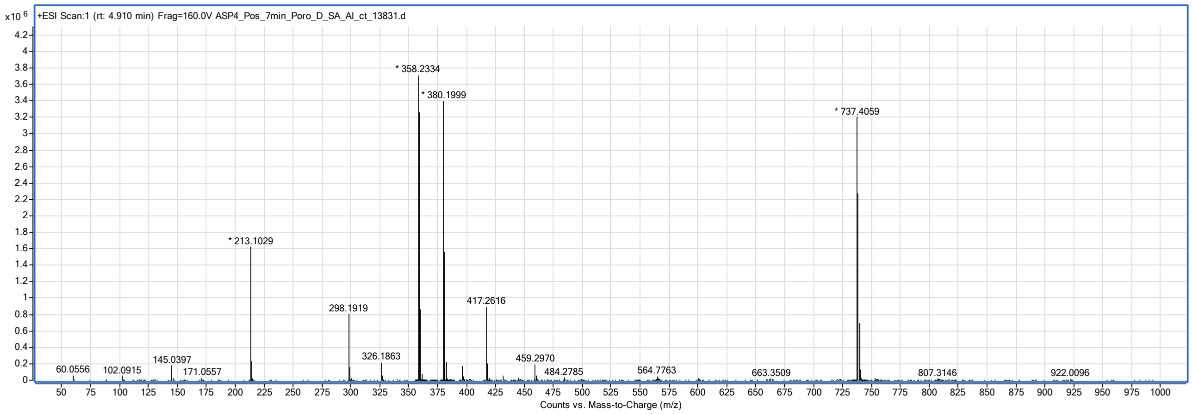

Figure S50. Extracted Mass Spectra from LC-MS Analysis for ASP4.

ASP5: 5F-MDMB-PICA

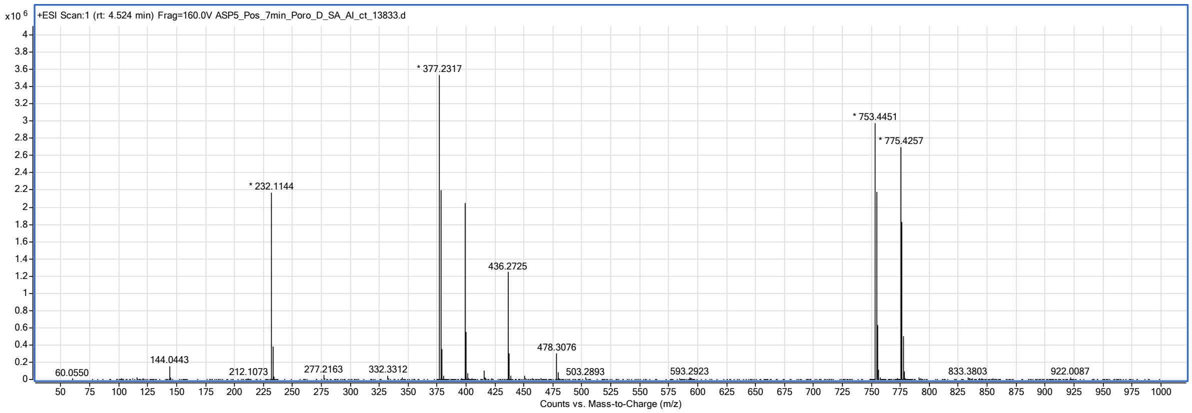

Figure S51. Extracted Mass Spectra from LC-MS Analysis for ASP5.

ASP6: MDMB-4en-PINACA

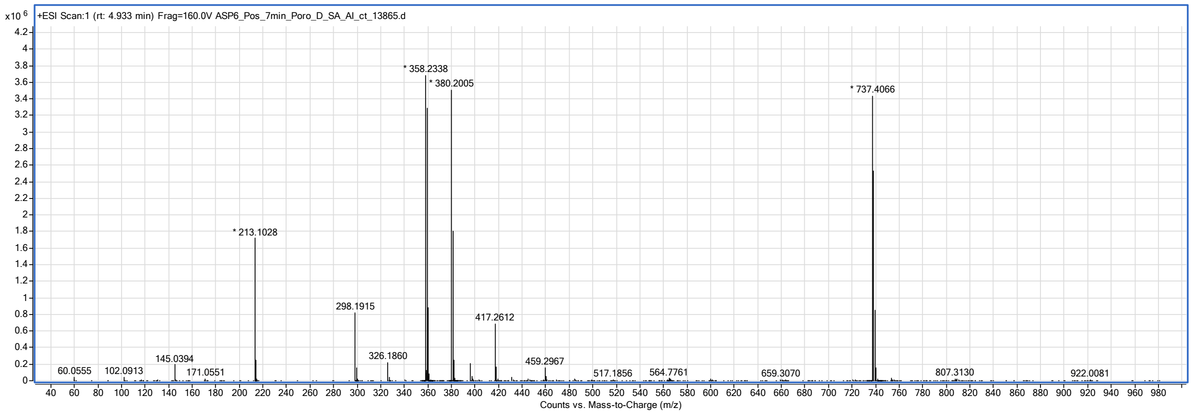

Figure S52. Extracted Mass Spectra from LC-MS Analysis for ASP6.

ASP8: 4F-MDMB-BINACA

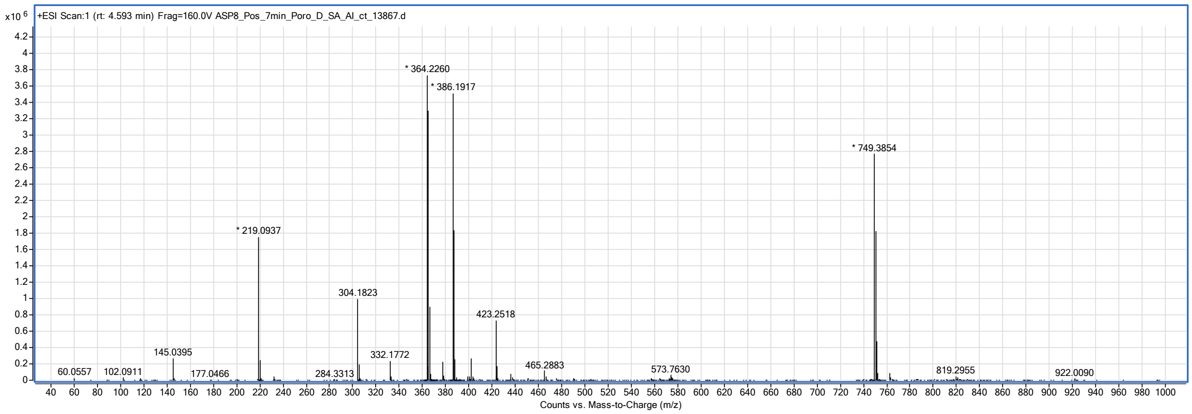

Figure S53. Extracted Mass Spectra from LC-MS Analysis for ASP8.

ASP11: MDMB-4en-PINACA

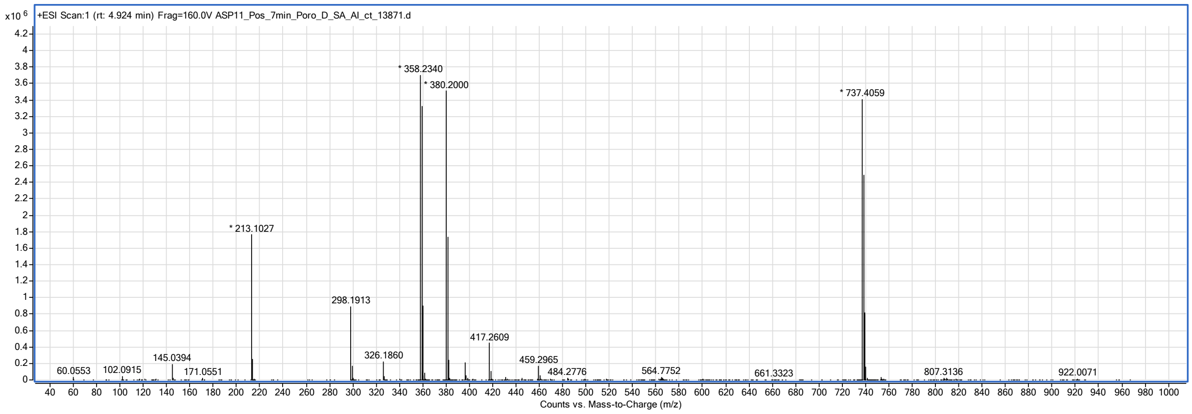

Figure S54. Extracted Mass Spectra from LC-MS Analysis for ASP11.

ASP12: MDMB-4en-PINACA

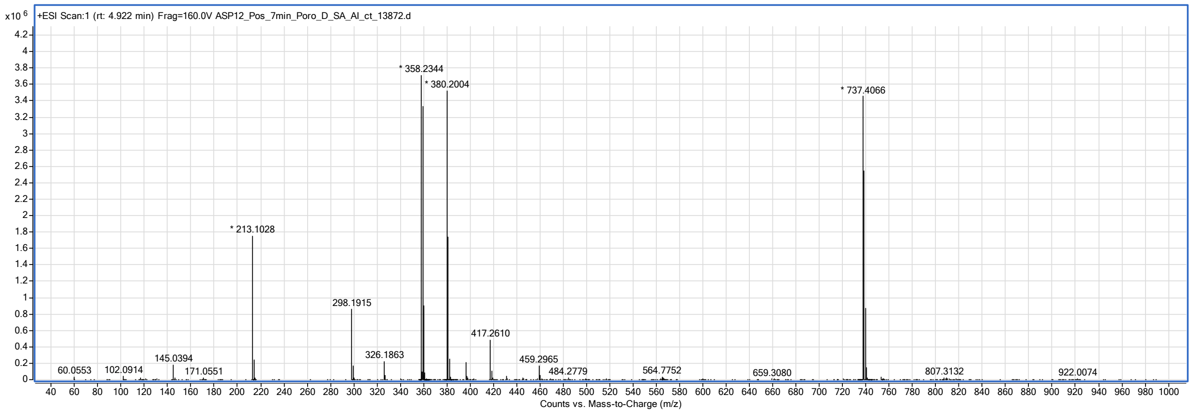

Figure S55. Extracted Mass Spectra from LC-MS Analysis for ASP12.

ASP13: MDMB-4en-PINACA

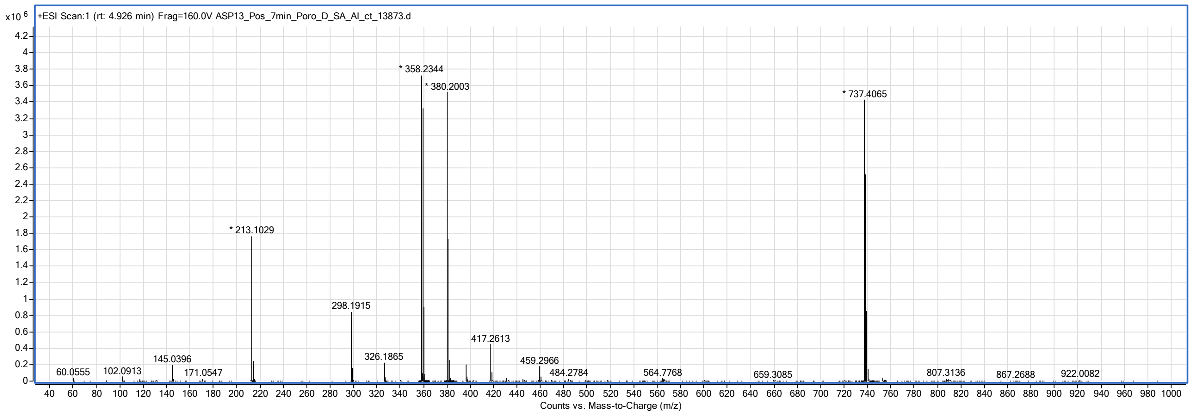

Figure S56. Extracted Mass Spectra from LC-MS Analysis for ASP13.

ASP14: 5F-MDMB-PINACA

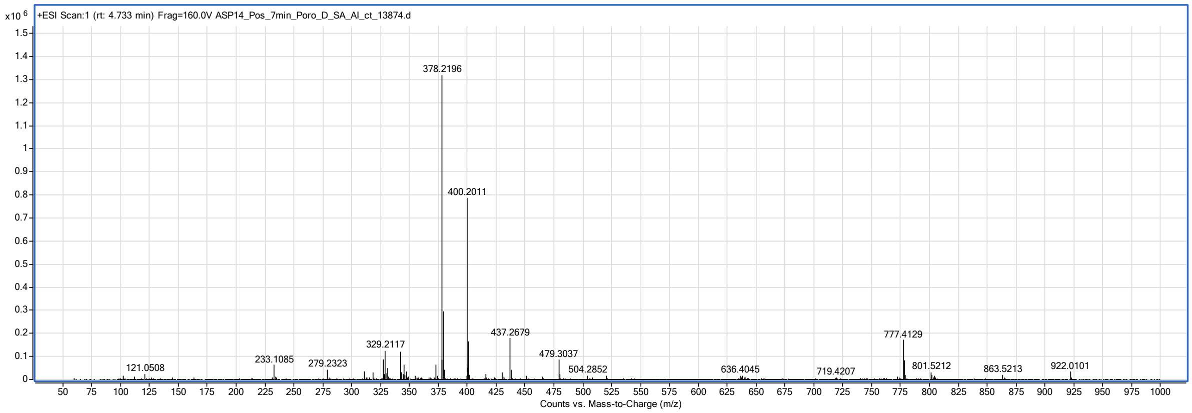

Figure S57. Extracted Mass Spectra from LC-MS Analysis for ASP14.

ASP15: 5F-MDMB-PINACA

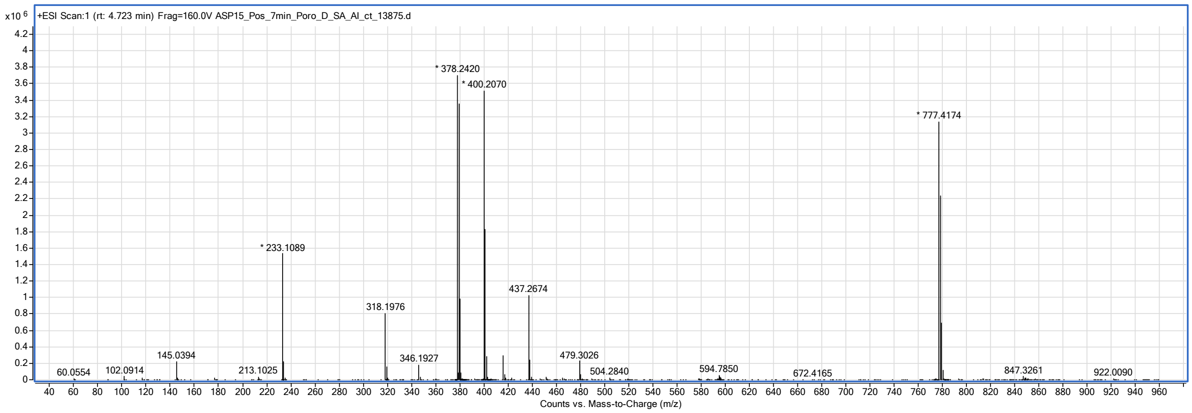

Figure S58. Extracted Mass Spectra from LC-MS Analysis for ASP15.

ASP16: 5F-MDMB-PINACA

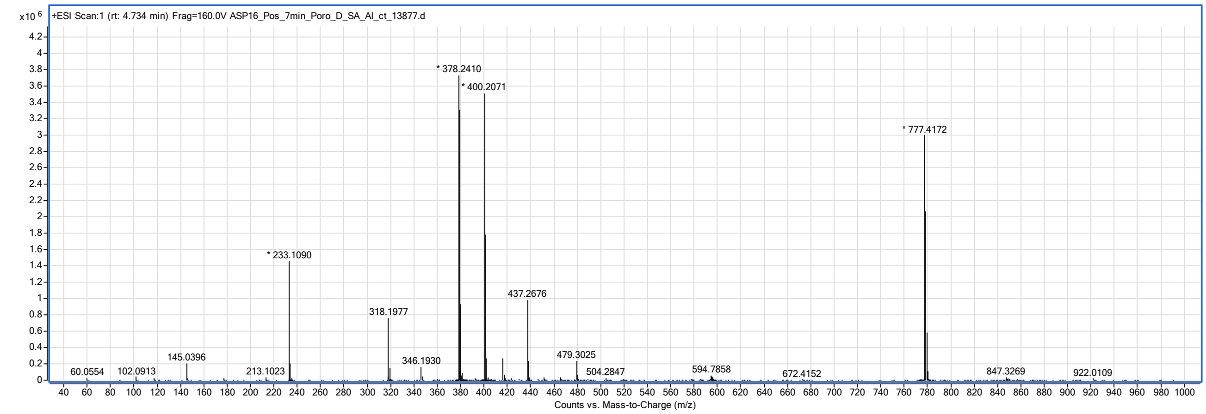

Figure S59. Extracted Mass Spectra from LC-MS Analysis for ASP16.

ASP17: 5F-MDMB-PINACA

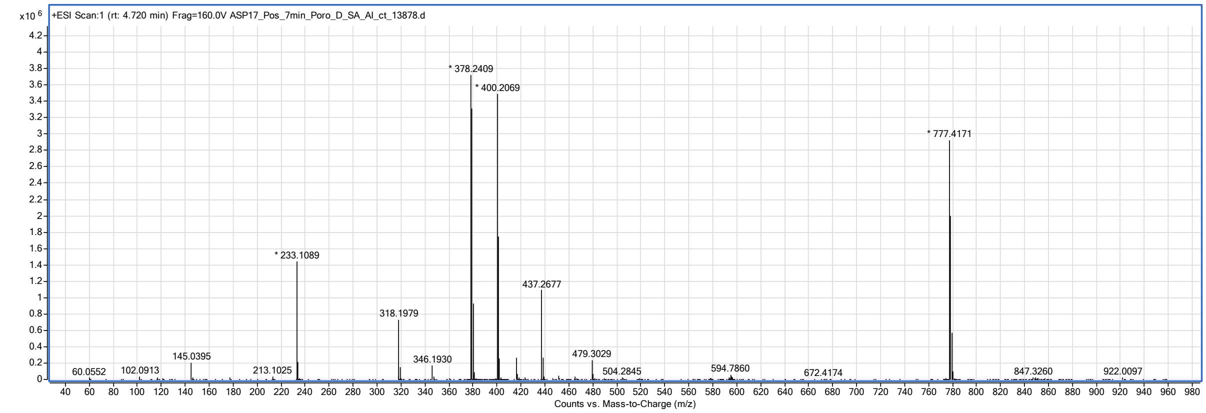

Figure S60. Extracted Mass Spectra from LC-MS Analysis for ASP17.

ASP18: 5F-PB-22

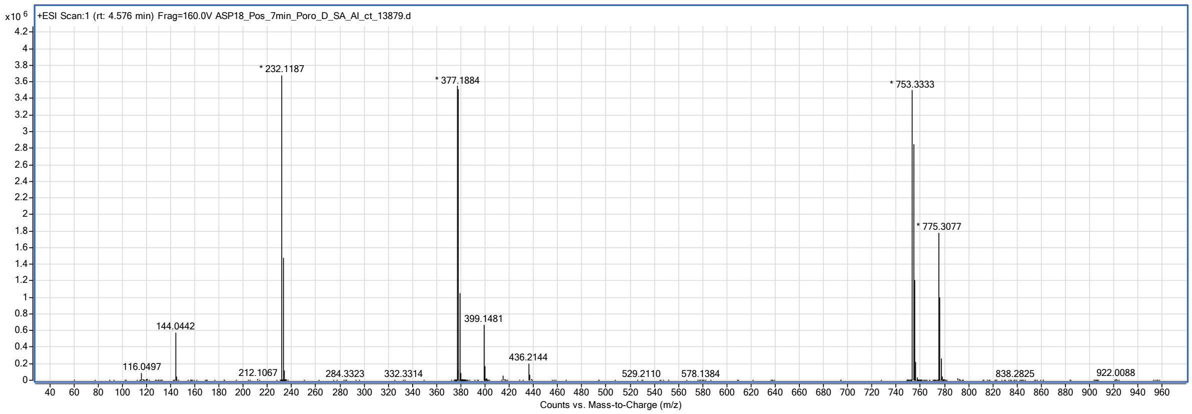

5F-AKB-48

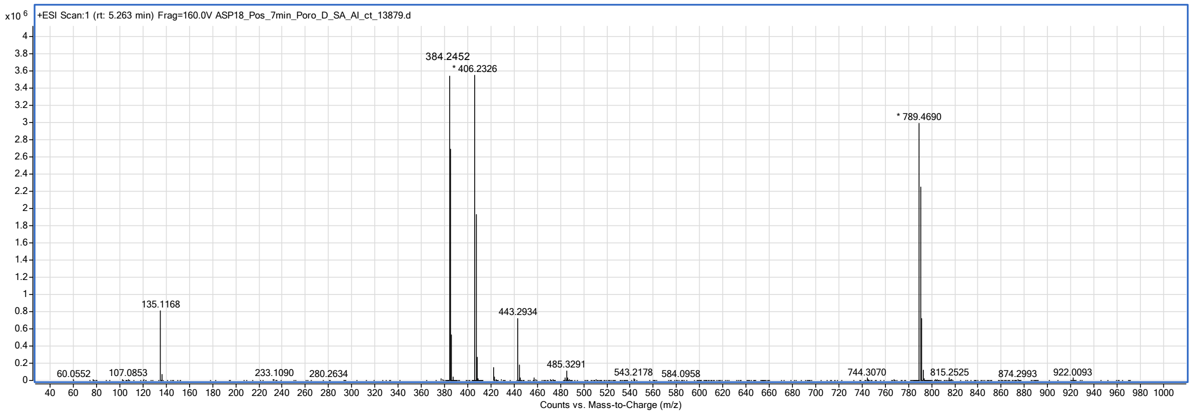

Figure S61. Extracted Mass Spectra from LC-MS Analysis for ASP18.

ASP19: 5F-MDMB-PICA

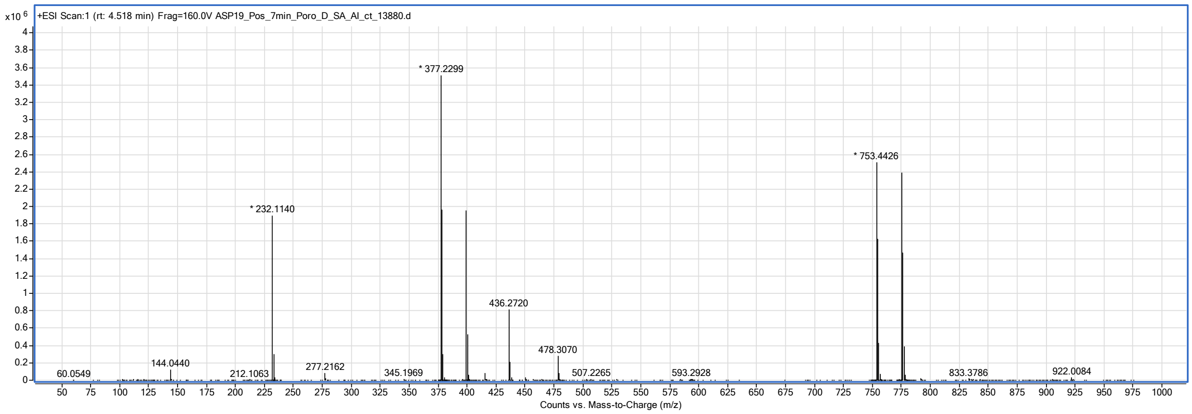

5F-AMB-PINACA

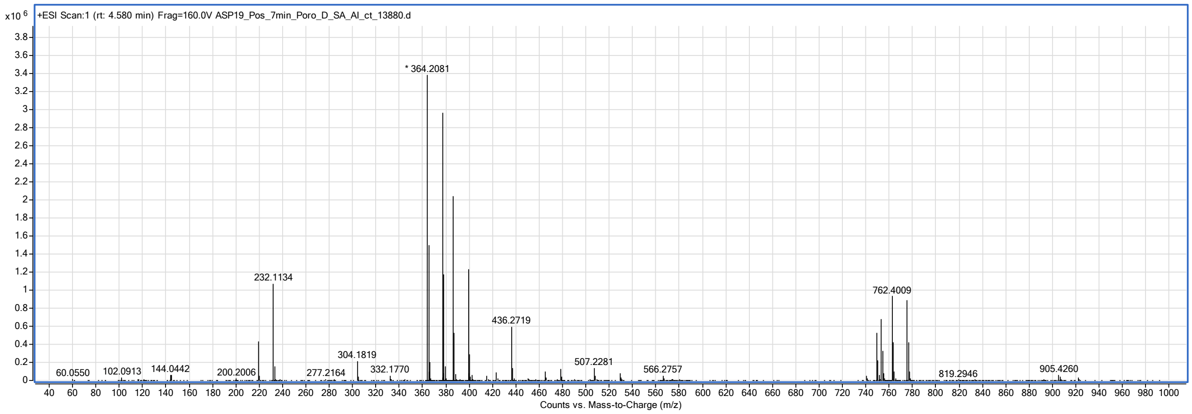

MDMB-4en-PINACA

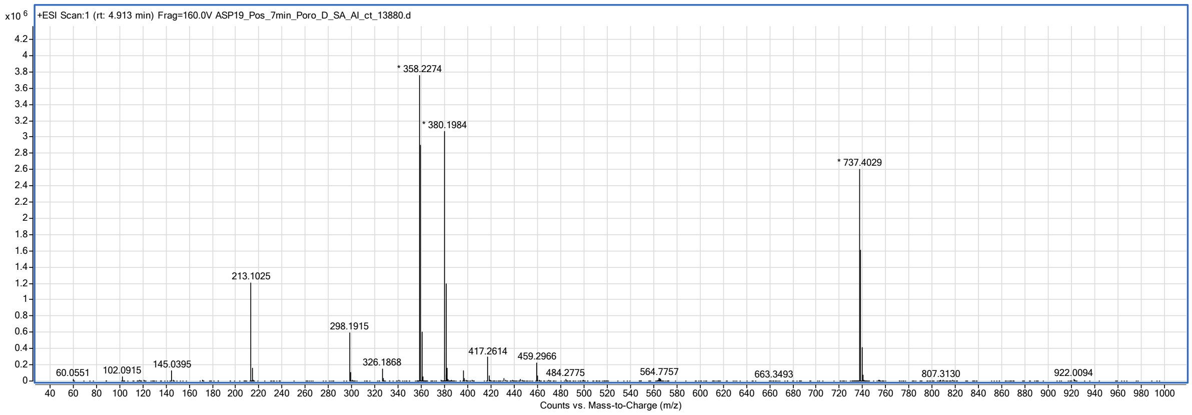

Figure S62. Extracted Mass Spectra from LC-MS Analysis for ASP19.

ASP22: MDMB-4en-PINACA

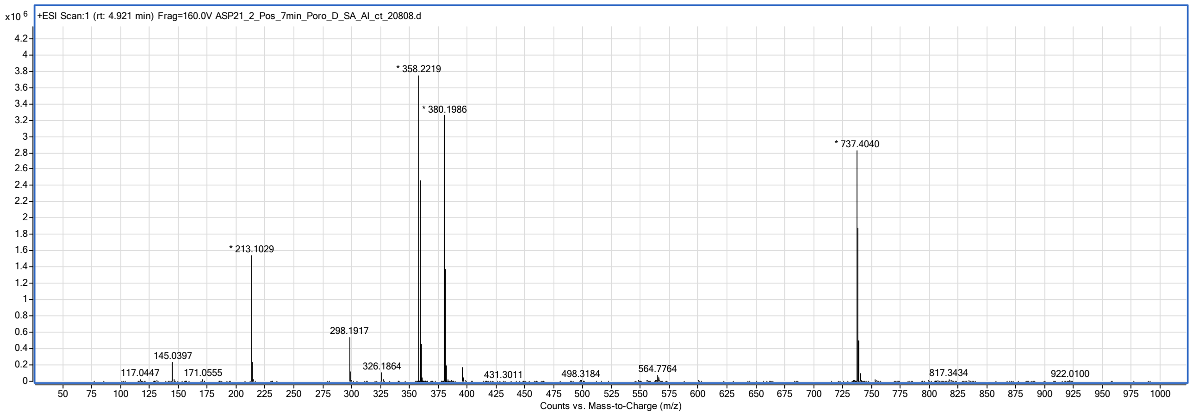

Figure S63. Extracted Mass Spectra from LC-MS Analysis for ASP22.

ASP23: MDMB-4en-PINACA

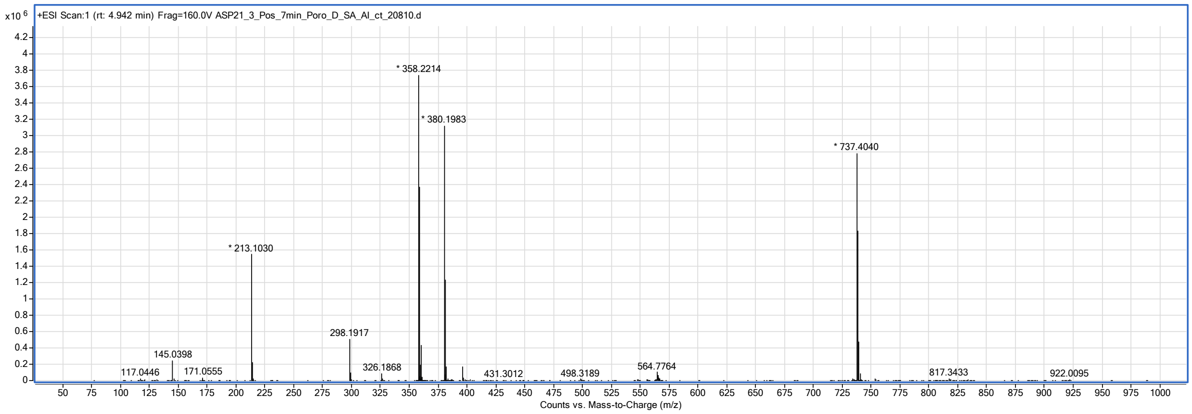

Figure S64. Extracted Mass Spectra from LC-MS Analysis for ASP23.

ASP24: MDMB-4en-PINACA

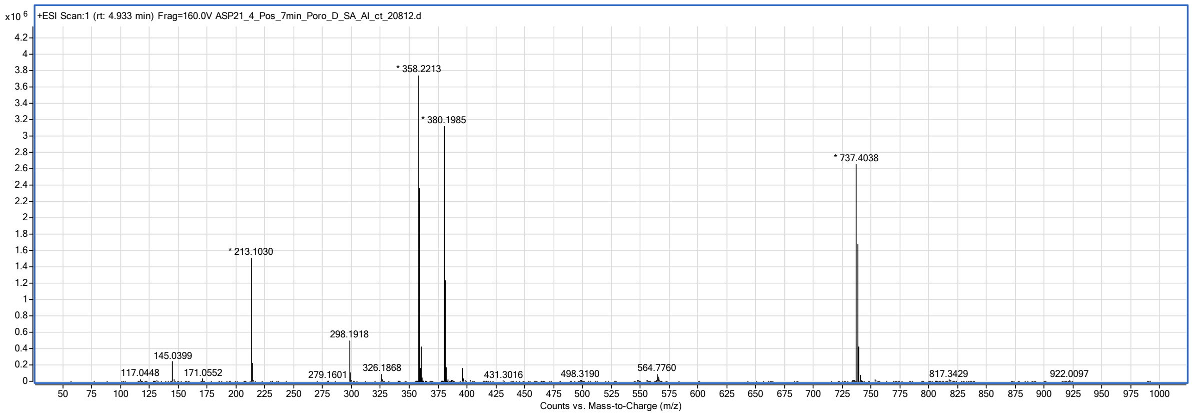

Figure S65. Extracted Mass Spectra from LC-MS Analysis for ASP24.

ASP25: 5F-MDMB-PICA

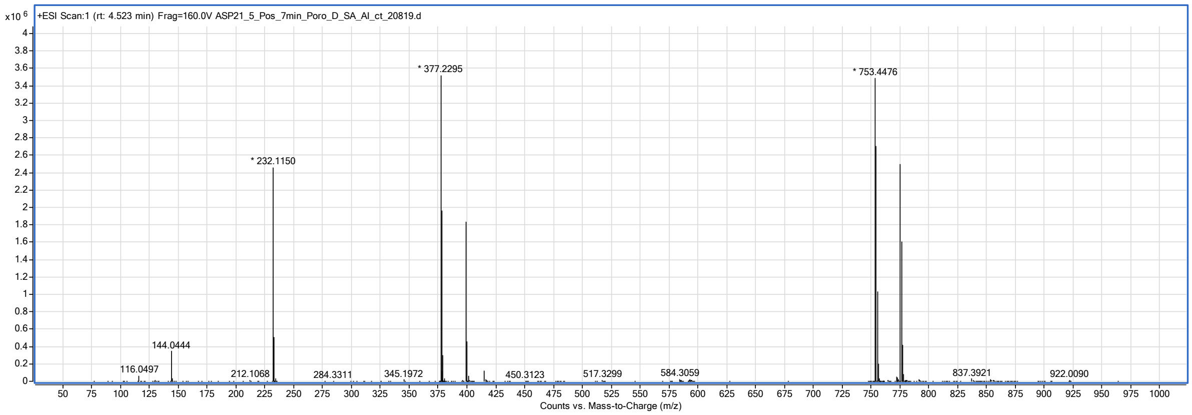

4F-MDMB-BINACA

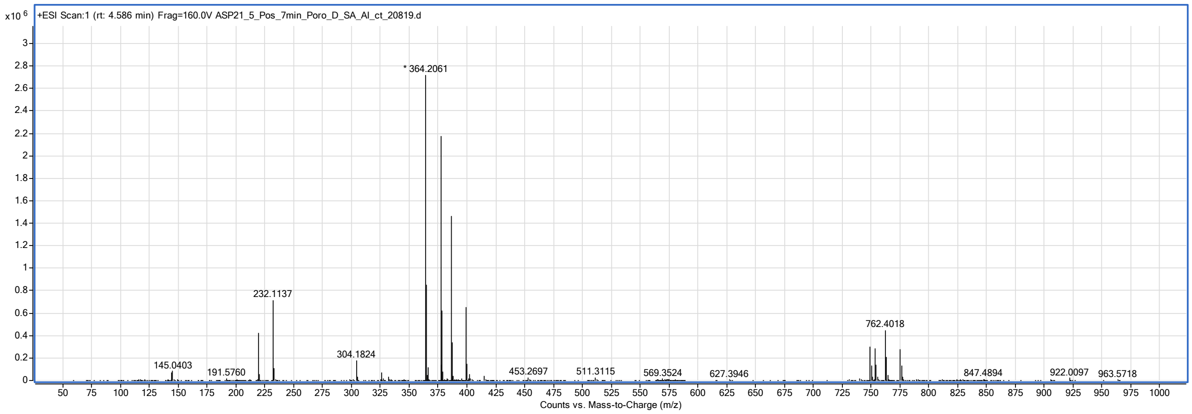

Figure S66. Extracted Mass Spectra from LC-MS Analysis for ASP25.

ASP26: MDMB-4en-PINACA

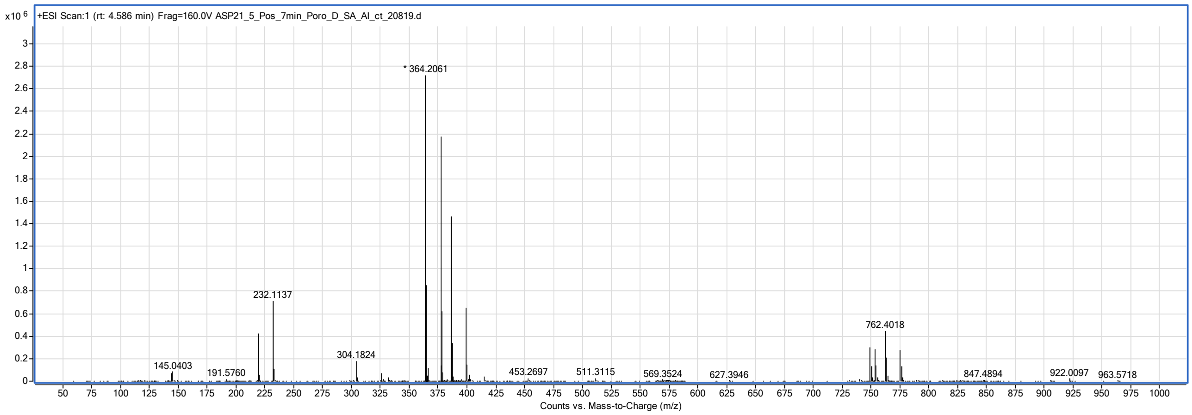

Figure S67. Extracted Mass Spectra from LC-MS Analysis for ASP26.

ASP28: 5F-MDMB-PICA

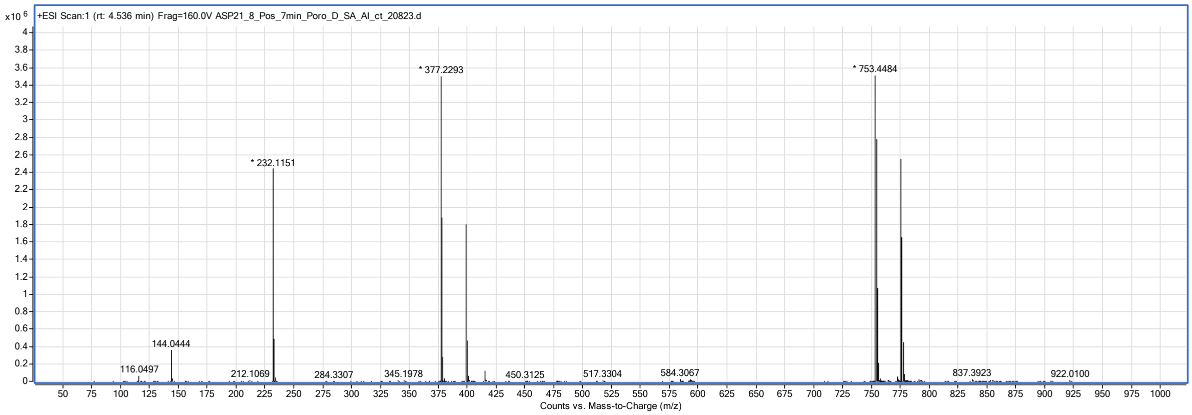

5F-MDMB-PINACA

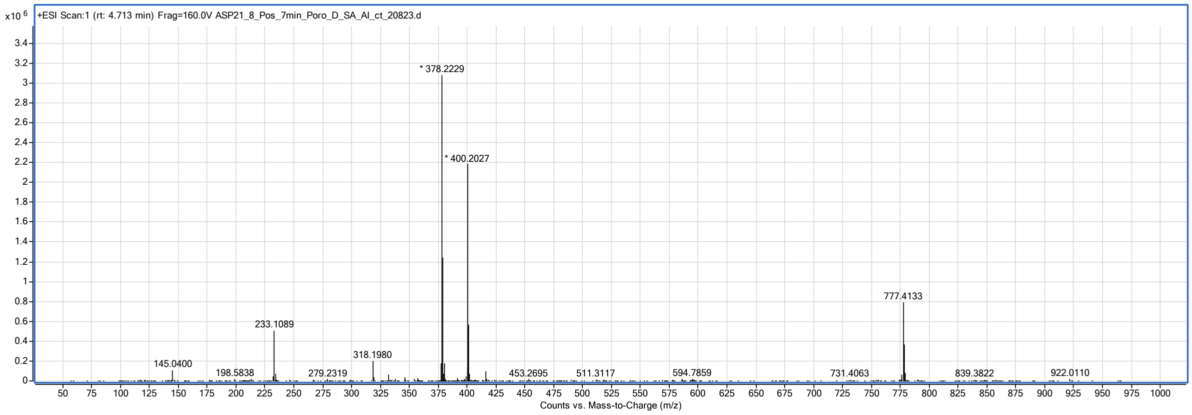

FUB-144

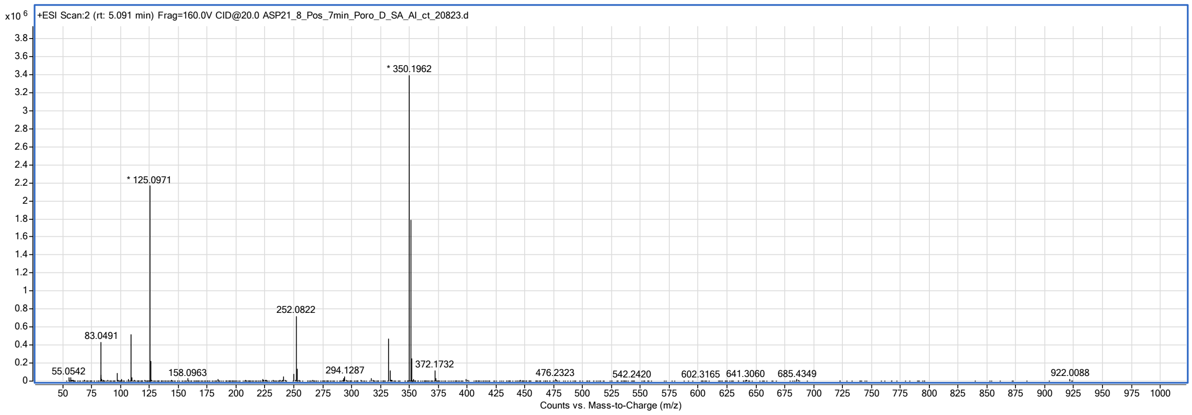

Figure S68. Extracted Mass Spectra from LC-MS Analysis for ASP28.

ASP29: MDMB-4en-PINACA

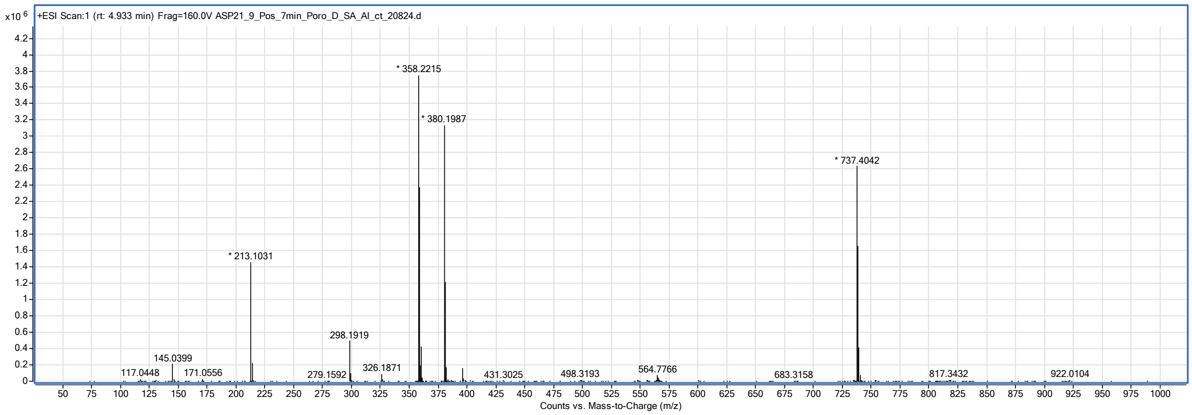

Figure S69. Extracted Mass Spectra from LC-MS Analysis for ASP29.

ASP30: 5F-MDMB-PICA

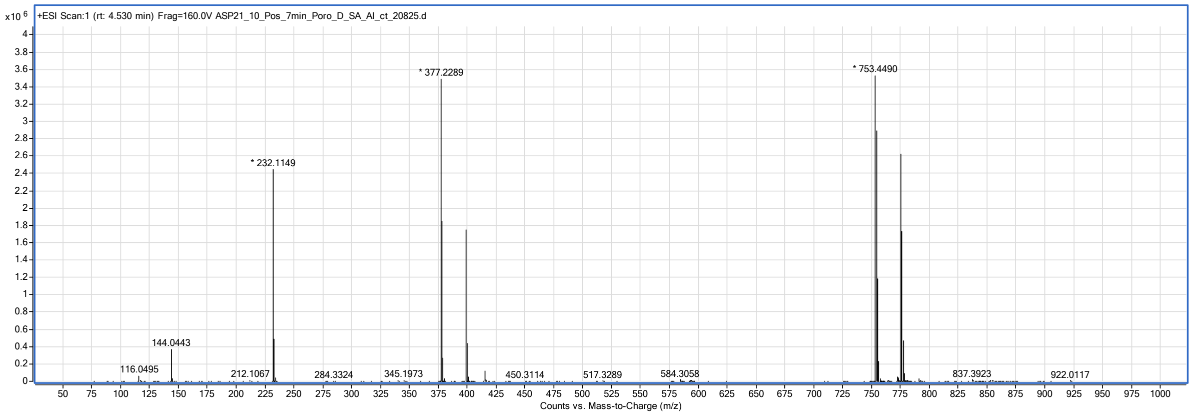

5F-MDMB-PINACA

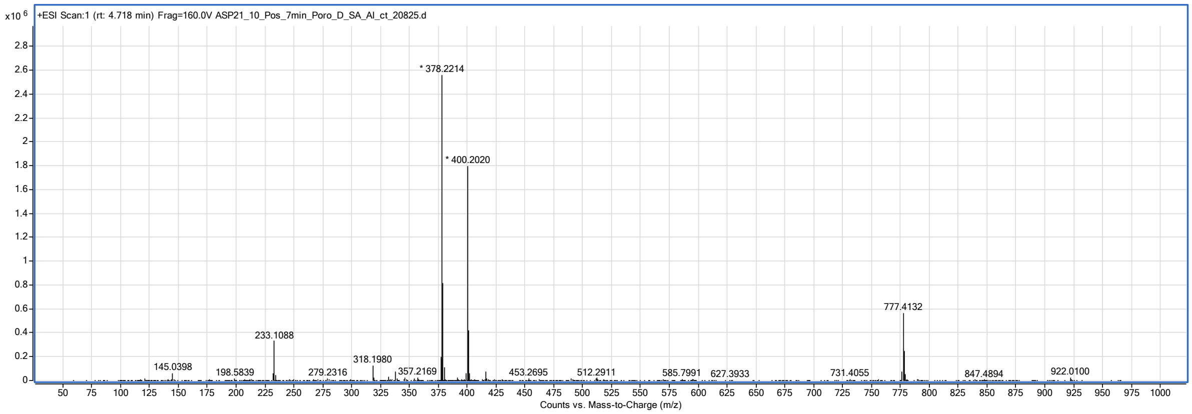

FUB-144

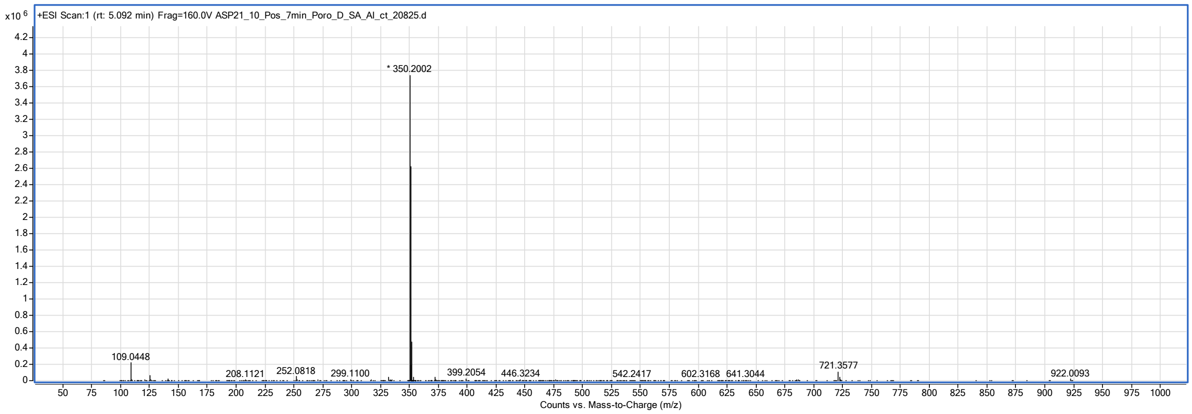

Figure S70. Extracted Mass Spectra from LC-MS Analysis for ASP30.

Tables

Table S1. Summary results for the confirmatory analysis using the combined TLC, NMR and LC-MS workflow. Only controlled substances are listed. Compounds that were detected in LC-MS but not identified in the NMR have been indicated with an Asterix (*).

Sample	Workflow Results
ASP 1	--
ASP 2	5F-MDMB-PINACA
ASP 3	5F-MDMB-PINACA, AMB-FUBINACA
ASP 4	MDMB-4en-PINACA
ASP 5	5F-MDMB-PICA
ASP 6	MDMB-4en-PINACA
ASP 7	--
ASP 8	4F-MDMB-BINACA
ASP 9	Cannabis*
ASP 10	--
ASP 11	MDMB-4en-PINACA
ASP 12	MDMB-4en-PINACA
ASP 13	MDMB-4en-PINACA
ASP 14	Cannabis*, 5F-MDMB-PINACA*
ASP 15	5F-MDMB-PINACA
ASP 16	5F-MDMB-PINACA
ASP 17	5F-MDMB-PINACA
ASP 18	5F-PB-22, 5F-AKB48
ASP 19	MDMB-4en-PINACA, 5F-MDMB-PICA, 5F-AMB-PINACA*
ASP 20	--
ASP 21	--
ASP 22	MDMB-4en-PINACA
ASP 23	MDMB-4en-PINACA
ASP 24	MDMB-4en-PINACA
ASP 25	5F-MDMB-PICA, 4F-MDMB-BINACA
ASP 26	MDMB-4en-PINACA

**ASP 27**	--
**ASP 28**	5F-MDMB-PICA, FUB-144, 5F-MDMB-PINACA*
**ASP 29**	MDMB-4en-PINACA
**ASP 30**	5F-MDMB-PICA, FUB-144, 5F-MDMB-PINACA*
**SP066**	5F-ADB
**SP067**	5F-PB-22
**SP068**	MDMB-CHMICA
**ASP 31**	--
**ASP 32**	--
**ASP 33**	--
**ASP 34**	MDMB-4en-PINACA
**ASP 35**	MDMB-4en-PINACA
**ASP 36**	--
**ASP 38**	--
**ASP 39**	--

Table S2. Results of trial of 181 paper (letters, cards etc.) samples seized from prisons highlighting the effect of changing detection threshold.

	High Sensitivity Threshold (~12% false positive threshold)	High Specificity Threshold (~6% false positive threshold)
Positive	50	47
False Negative	14	17
Negative	103	110
False Positive	14	7
